# Exploring causality from observational data: An example assessing whether religiosity promotes cooperation

**DOI:** 10.1017/ehs.2023.17

**Published:** 2023-06-27

**Authors:** Daniel Major-Smith

**Affiliations:** Centre for Academic Child Health, Population Health Sciences, Bristol Medical School, University of Bristol, Bristol BS8 2BN, UK

**Keywords:** Avon Longitudinal Study of Parents and Children, Religion, Cooperation, Causal Inference, Sensitivity Analysis

## Abstract

Causal inference from observational data is notoriously difficult, and relies upon many unverifiable assumptions, including no confounding or selection bias. Here, we demonstrate how to apply a range of sensitivity analyses to examine whether a causal interpretation from observational data may be justified. These methods include: testing different confounding structures (as the assumed confounding model may be incorrect), exploring potential residual confounding and assessing the impact of selection bias due to missing data. We aim to answer the causal question ‘Does religiosity promote cooperative behaviour?’ as a motivating example of how these methods can be applied. We use data from the parental generation of a large-scale (*n* = approximately 14,000) prospective UK birth cohort (the Avon Longitudinal Study of Parents and Children), which has detailed information on religiosity and potential confounding variables, while cooperation was measured via self-reported history of blood donation. In this study, there was no association between religious belief or affiliation and blood donation. Religious attendance was positively associated with blood donation, but could plausibly be explained by unmeasured confounding. In this population, evidence that religiosity causes blood donation is suggestive, but rather weak. These analyses illustrate how sensitivity analyses can aid causal inference from observational research.

## Graphical abstract

**Figure:**
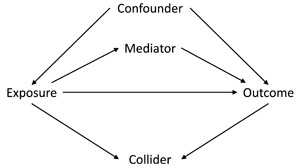

**Social media summary** (haiku):

Confounding misleads
& selection collides; Our
Inferences blurred

Are results casual?
Sensitivity tests can
Explore assumptions

## Introduction

Causal inference is a key goal of science. Studies which randomly (or quasi-randomly) allocate participants to conditions – such as randomised-controlled trials, instrumental variable analysis, difference-in-differences approaches and regression discontinuity designs – are traditionally viewed as providing stronger evidence for causality than observational studies that do not include such (quasi-)random elements (Hernán & Robins, 2020; Lawlor et al., 2016; Marinescu et al., 2018). While such designs undoubtedly provide stronger evidence for causality, much research – including in the field of evolutionary human sciences – has the aim of causal inference, yet in many cases (quasi-)random designs are difficult or impossible to implement. To use examples relevant to this study, it is not possible, or ethical, to randomly allocate individuals to different religious beliefs or behaviours in a randomised-controlled trial, while (quasi-)random sources of variation required for instrumental variable analyses may be difficult to identify for factors such as religiosity. While designs which provide greater evidence for causality should of course be considered, these issues mean that researchers often have to rely on observational data to try and answer these causal questions.

Inferring causality from observational data is nonetheless possible, if various assumptions are met (Hernán & Robins, 2020; Pearl et al., 2016; Pearl & Mackenzie, 2019). These assumptions include: (i) the set of confounding variables being correctly specified and no residual confounding (i.e. no confounder bias); and (ii) no bias due to selection into the study (i.e. no selection bias). These are strong, and often largely unverifiable, assumptions, but if they are met then a causal interpretation from observational data may be warranted. Various sensitivity analyses to explore these assumptions, and potentially correct for any resulting bias, have been developed and can be employed to assess whether these assumptions have plausibly been met, and therefore whether a causal interpretation from observational data may be justifiable. These sensitivity analyses are frequently employed in epidemiology (Lee et al., 2021; Tompsett et al., 2018; VanderWeele & Ding, 2017; White et al., 2011). In this paper, we demonstrate how such sensitivity analyses to try and infer causality from observational data can be used in the field of evolutionary human sciences, and related disciplines, where such approaches are currently less common. Throughout this paper we aim to answer the causal question ‘Does religiosity promote cooperative behaviour?’ as a motivating example of how these methods can be applied.

### Assumptions required for causal inference from observational data

We will first describe some of the assumptions required for causal inference from observational data in more detail, and approaches that can be used to explore, and potentially overcome, these issues. The first two problems concern confounding, regarding the assumed confounding structure of measured variables and the possibility of residual confounding due to unmeasured or imperfectly measured covariates, while the third problem concerns selection bias due to missing data. We also note that measurement error and reverse causality are other sources of bias to be considered when assessing causality (Hernán & Robins, 2020; Lash et al., 2021; VanderWeele et al., 2016), but we predominantly focus on confounding and selection bias in this paper. Directed acyclic graphs (also known as causal graphs; Greenland et al., 1999; Hernán & Robins, 2020) are a useful tool for presenting the hypothesised causal relations between variables, identifying confounders, mediators and colliders, and informing statistical analyses (e.g. choice of covariates, implications of missing data), and will be used throughout this paper. Scripts simulating these assumptions, how violating them can lead to bias, and methods to explore and overcome them, are available for readers on GitHub (https://github.com/djsmith-90/AnalysisCode_BloodDonation_B4030).

#### (i) Mis-specified confounding model

**Figure 1.**
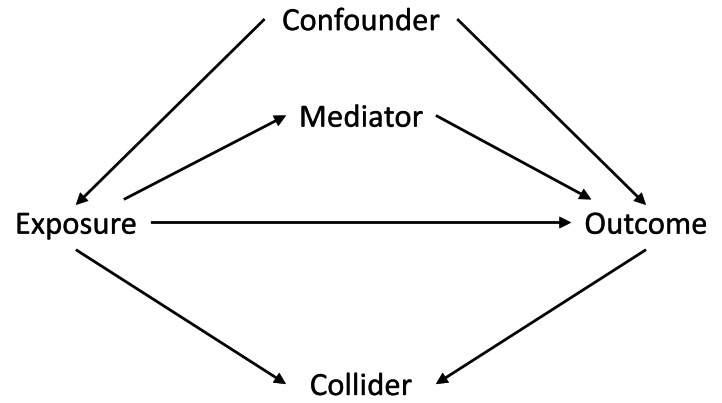
Directed acyclic graph describing different causal structures and how they can be encoded in such causal graphs. The arrows represent the direction of causality; for instance, the arrow from ‘Exposure’ to ‘Outcome’ indicates that the exposure causes the outcome. A confounder is a variable which causes both the exposure and the outcome, as indicated by arrows from ‘Confounder’ to both ‘Exposure’ and ‘Outcome; as information can ‘flow’ from the exposure to the outcome via confounders, it is necessary to adjust for all confounders – which blocks these back-door paths – in order to obtain an unbiased causal estimate of the exposure–outcome association. A mediator is a variable which is caused by the exposure (arrow from ‘Exposure’ to ‘Mediator’), which in turn causes the outcome (arrow from ‘Mediator’ to ‘Outcome’); as mediators are part of the pathway by which the exposure causes the outcome, adjusting for a mediator will result in a biased estimate of the exposure–outcome association. A collider is a variable which is caused by both the exposure and the outcome (arrows from both ‘Exposure’ and ‘Outcome’ to ‘Collider’). It is not necessary to adjust for a collider, as colliders ‘block’ the flow of information between other variables; adjusting for a collider, however, opens these pathways, potentially resulting in biased associations. Using directed acyclic graphs to represent the assumed causal structure of the data can help identify whether covariates are confounders, mediators or colliders, and therefore which variables to statistically adjust for to return an unbiased causal estimate, given the assumptions embedded in the causal graph.

The first assumption concerns the confounding structure of the observed variables and whether this has been specified correctly. A confounder is a variable which causes both the exposure and the outcome, meaning that statistical adjustment for all confounders is required for unbiased causal inferences. However, in a given study the causal relations between all covariates, exposures and outcomes is often not known with certainty. As such, it can be difficult to know which potential covariates are confounders (causing both the exposure and the outcome), mediators (caused by the exposure which in turn cause the outcome) or colliders (caused by both the exposure and the outcome); see Figure 1 for how these different data structures can be encoded in directed acyclic graphs. This is especially difficult to know when all variables were collected at approximately the same time. Nonetheless, understanding the causal structure of the data – or making assumptions about the underlying causal structure of the data, at least – is a necessary step when trying to estimate causal effects, as not adjusting for a confounder, adjusting for a mediator, or adjusting for a collider can all result in biased causal estimates (VanderWeele et al., 2021).

**Figure 2.**
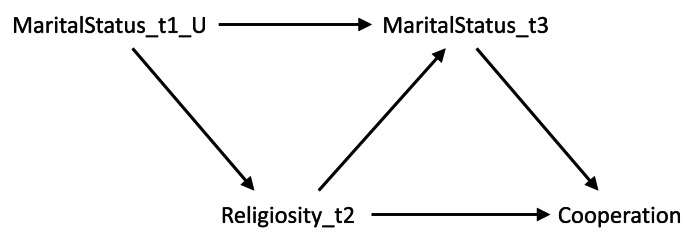
Directed acyclic graph showing potential reciprocal causation between religiosity and marital status, and how marital status can be both a confounder (at time 1) and a mediator (at time 3) of the religiosity–cooperation association. The ‘_U’ after MaritalStatus_t1 means this variable is unobserved (as in our real-word data example), resulting in an inability to estimate an unbiased causal estimate of the association between Religiosity_t2 and Cooperation.

It is also possible that some covariates may be both confounders and mediators (i.e. bidirectional causation), meaning that – in the absence of longitudinal, repeated, data – calculating causal estimates may be even more difficult as both adjusting and not adjusting for the covariate will result in bias. To use an example relevant to this study, imagine that our exposure is religiosity, our outcome is cooperation and marital status is a covariate. Marital status at time 1 may cause both religiosity at time 2 (with marriage causing an increase in religiosity) and marital status at time 3 (with being married at time 1 causing being married at time 3); religiosity at time 2 may in turn cause marital status at time 3 as well (with religious individuals being more likely to get or remain married). In this example marital status is both a confounder (at time 1) and a mediator (at time 3) of the religiosity–cooperation relationship (Figure 2). If we only had marital status observed at time 3, then we would not be able to estimate an unbiased causal effect of religiosity on cooperation using standard regression-based approaches, as both adjusting and not adjusting for marital status at time 3 will result in bias. However, if we had both measures of marital status we could adjust for marital status at time 1 – the confounder – but not marital status at time 3 – the mediator – to obtain an unbiased causal estimate (Major-Smith et al., 2022).

In situations such as this – and in the absence of additional data – it can appear difficult, if not impossible, to overcome these issues and select the correct confounders necessary to estimate unbiased causal effects using standard regression-based approaches. But making our assumptions clear and noting the limitations inherent in any such analysis is a useful first step when assessing the evidence for such causal claims. We can also run sensitivity analyses to explore the extent to which changing the assumptions regarding confounding impacts results. For instance, we can first run a model which only includes assumed confounders (and excludes variables which may be both confounders and mediators) and then run another model which includes both assumed confounders and assumed confounders/mediators. Assuming no other sources of bias, these two scenarios can be thought of as bracketing the minimum and maximum plausible effect estimates. By removing assumed confounders/mediators, the model with only assumed confounders is likely to ‘under-control’ for confounding; in contrast, the model with both assumed confounders and assumed confounders/mediators is likely to ‘over-control’ for confounding. The true causal effect is likely to fall between these two values. If both analyses provide similar results, then we can have greater confidence that bias due to mis-specified confounding may be minimal; if the analyses provide divergent results, then we may not be able to specify a precise causal effect, but may be able to indicate a range of plausible values in which the true effect falls. The choice of confounders, and whether they may also be mediators, is a qualitative judgment call informed by prior literature, theory, expert subject knowledge, logical considerations, or simply best guesses (if no other information is available).

#### (ii) Residual confounding

A second assumption required for an unbiased causal interpretation is no residual confounding. For instance, perhaps some unmeasured confounder causes both the exposure and outcome and was not included in our analysis model (either because it was overlooked or simply not measured), or the measured confounders included contain measurement error so were not sufficient to remove all sources of confounding (Greenland, 1980; Hernán & Robins, 2020). The presence of residual confounding will alter the exposure–outcome association, resulting in biased causal inferences. Various quantitative bias analyses have been developed to assess the impact of residual confounding (Kawabata et al., 2022), which often rely on estimating the structure and magnitude of residual confounding (Lash, 2021; Lash et al., 2014). However, the extent of residual confounding (if any) is unfortunately often difficult to know with any degree of certainty. Rather than modelling the assumed residual confounding structure, alternative – and much simpler – sensitivity analyses have been developed which estimate the strength of unmeasured confounding necessary to alter a study's conclusions (Harada, 2013; VanderWeele & Ding, 2017), and will be used in this paper. If the size of this effect is larger than what would be considered reasonable – itself a subjective judgement which must be justified – then we can have confidence that our results are unlikely to be substantially biased by residual confounding. Alternatively, if the amount of residual confounding necessary to alter the previous result is minor, then small levels of residual confounding can dramatically alter conclusions, and we would have less confidence that these results are robust to residual confounding. While these methods cannot tell us whether our observed effect estimate is correct, they can say whether the direction of said effect is plausible, even in the presence of residual confounding.

#### (iii) Selection bias

A third assumption required for unbiased causal inference is no bias due to selection. Selection bias can occur when either selection into the study sample (e.g. study recruitment), or selection into the analytic sample (e.g. loss to follow-up), or both, is non-random with respect to the target population (Hernán & Robins, 2020; Lu et al., 2022). This can result in bias when both the exposure and the outcome are associated with selection. This occurs because subsequent analyses implicitly condition on a collider (i.e. selection into the study), which can bias exposure–outcome associations (Griffith et al., 2020; Munafò et al., 2018). Although study recruitment is a key source of selection bias (Lu et al., 2022; Stamatakis et al., 2021), in this paper we focus on potential selection bias due to missing data after study recruitment for two main reasons: (i) recruitment into the cohort study used here (the Avon Longitudinal Study of Parents and Children) is known to be largely representative of the target population (Boyd et al., 2013; Fraser et al., 2013), meaning that selection bias due to study recruitment is likely to be relatively small; and (ii) missing data due to loss to follow-up is known to be present in this study, and methods such as multiple imputation – which will be used here – can be employed in these situations to try and overcome selection bias and return unbiased estimates (although multiple imputation itself rests on various assumptions, as discussed in more detail in the Methods section; Hughes et al., 2019; van Buuren, 2018; White et al., 2011).

### Motivating example: Religion and cooperation

The evolution of religious beliefs and behaviours has been explored for well over a century since the origins of anthropology (Bowie, 2006), but the adaptive function(s) – if any – underpinning this suite of behaviours is a matter of considerable debate. One prominent theory suggests that religions are culturally evolved institutions adapted to promote cooperation among members of the cultural group (Henrich et al., 2010; Lang et al., 2019; Norenzayan et al., 2016; Purzycki et al., 2016; Wright, 2010). Here, we define ‘cooperation’ as a behaviour which evolved to provide a fitness benefit to others, and so encompasses both altruistic and mutually beneficial behaviour (West et al., 2007). While the specific mechanisms are debated – for instance, it is not clear whether religion may promote cooperation due to fear of supernatural punishment (Norenzayan et al., 2016), as a signal of trustworthiness (Power, 2017), other reputational considerations (Ge et al., 2019), as a result of cultural group selection (Richerson et al., 2016), or a combination of these theories – overall there is a broad prediction that religious individuals are likely to be more cooperative than non-religious (or less-religious) individuals.

This prediction appears to be well supported, with numerous observational (Galen, 2012; Ge et al., 2019; Henrich et al., 2010; Lang et al., 2019; Power, 2017; Purzycki et al., 2016; Schulz et al., 2019) and experimental (Shariff et al., 2016) studies finding that religiosity is associated with greater levels of cooperation. However, many of these observational studies are subject to the potential biases described above (Billingsley et al., 2018; Hernán & Robins, 2020) such as residual confounding, or factors such as social desirability bias (where religious individuals may profess to be more cooperative in self-report measures, but not in actual behaviour). Additionally, not all observational studies find this positive association between religiosity and cooperation, or the relationship may be more complicated than often assumed (Vardy & Atkinson, 2022). As an example, while some studies have reported a positive association between religiosity and blood donation (Beyerlein, 2016) – the measure of cooperation used in the present study – others found no relationship (Zucoloto et al., 2022), or no relationship once adjusting for relevant sociodemographic confounders (Gillum & Masters, 2010).

While experimental studies should be free of these confounding biases, they pose their own problems, such as concerns regarding external validity and the real-world interpretation of such interventions. Publication bias may also temper the conclusions of previously published meta-analyses of experimental studies, which indicated a small but reliable positive association between religious priming and cooperation (Shariff et al., 2016); subsequent large-scale pre-registered studies have failed to replicate these religious priming effects (Billingsley et al., 2018; Gomes & Mccullough, 2015), while more recent meta-analyses suggest that the conclusions of the previous meta-analysis by Shariff and colleagues (Shariff et al., 2016) may have been largely a result of publication bias (van Elk et al., 2015).

Although there does appear to be a general positive association between religiosity and cooperation, questions nonetheless remain regarding whether this reflects a true causal effect. Here, we aim to explore this question using data from a large-scale UK population-based cohort study with detailed information on religious/spiritual beliefs and behaviours and potential confounders of the religiosity–cooperation association, using ‘blood donation’ as the measure of cooperation. This paper aims to answer the causal question ‘Does religiosity promote cooperative behaviour?’ Of course, given the known difficulties of inferring causation from observational studies discussed above, this does not mean that the causal effect will be estimated correctly, but being clear about the aims of the research removes potential ambiguity over the research question and can help facilitate discussion regarding the interpretation of results (Hernán, 2018). By conducting an extensive battery of sensitivity analyses intended to explore the assumptions required to infer causality from observational data, it is hoped that this paper can provide an example of how researchers can attempt to assess causality when working with messy, real-world, observational data, while also contributing to the literature on religion and cooperation.

## Methods

This paper is a Registered Report, and the registered analysis plan can be found at https://osf.io/z5gcm/. The research question, methods and analyses reported below are identical to those specified in the analysis plan, with only a few superficial updates and corrections (see Section S1 of the supplementary information for full details).

### The Avon Longitudinal Study of Parents and Children – study description

Pregnant women resident in Bristol and surrounding areas in Southwest England with expected dates of delivery between 1 April 1991 and 31 December 1992 were invited to take part in the study. The initial number of pregnancies enrolled was 14,541, of which there were a total of 14,676 foetuses, resulting in 14,062 live births and 13,988 children who were alive at 1 year of age (Boyd et al., 2013; Fraser et al., 2013).

For each mother, we also included their associated partner, usually the father of the study child. Partners/fathers (hereafter ‘partners’) were not formally enrolled into the study, but were given partner-based questionnaires by the mother (if she had a partner and chose to invite them). This means that partner-based questionnaires may not have been completed by the same partner over time. However, as all partner-based data used in the present study were collected over two questionnaires conducted during pregnancy, the number of such cases is relatively small (27 cases in total; less than 0.2% of the total sample). While this is unlikely to result in any meaningful bias, we have excluded all partners where the identity is known to have changed over the time-frame of this study. Furthermore, although approximately 2000 partners never participated in the study, all potential partners have been included here to show levels of missing data, and because many of these partners have data from questionnaires completed by the mother about the partner which can be used to provide information on these partners (e.g. in multiple imputation analyses). The full partners sample therefore includes all potential partners, regardless of missing data status, other than the small number where the identity is known to have changed over the course of the study period assessed here (in addition to the further exclusions detailed below; for more information on this partners cohort, see Northstone et al., 2023).

For all analyses, we have only included pregnancies resulting in a live birth, removed one pregnancy if the mother had two pregnancies enrolled in the study (to avoid repeated data from the same parent), and dropped observations for participants who withdrew consent for their data to be used. As blood donors have to be 17 years of age or older, all mothers and partners younger than 17 when becoming pregnant were also excluded from analyses. Additionally, as certain religions, such as Jehovah's Witnesses and Rastafarians, may have proscriptions prohibiting blood donation, we have removed these individuals from analyses (the number of such individuals is small, *ca*. 60 in total, <0.5% of the total sample). After all exclusions, a total of 13,477 mothers and 13,424 partners were included in the final datasets for analysis. Please note that the study website contains details of all the data that is available through a fully searchable data dictionary and variable search tool: http://www.bristol.ac.uk/alspac/researchers/our-data/.

Ethical approval for the study was obtained from the Avon Longitudinal Study of Parents and Children Ethics and Law Committee and the Local Research Ethics Committees. Informed consent for the use of data collected via questionnaires and clinics was obtained from participants following the recommendations of the Avon Longitudinal Study of Parents and Children Ethics and Law Committee at the time.

### Exposures

Given the variation in definitions of religiosity, which may affect associations with cooperation (Galen, 2012), here we will explore three broad facets of religiosity (Table 1): religious belief (belief in God/a divine power), religious affiliation (self-reported membership of a religious group) and religious attendance (frequency of attendance at a place of worship). Even though these questions originally had more than two response options, in this paper we have coded each of these religiosity exposures as binary variables (e.g. for the religious belief exposure combining ‘no’ and ‘not sure’ together to indicate a lack of belief, as compared against ‘yes’). While this results in a loss of information, many of the sensitivity analyses applied here require either continuous or binary data; we believe that the benefits of being able to use these methods compensates for this loss of information. Supporting this decision, previous research in this population has suggested that although individuals who answered ‘no’ to religious belief differ somewhat from those who answered ‘not sure’ with respect to sociodemographic factors, this difference is smaller than both compared against those who responded ‘yes’ (Major-Smith et al., 2022). The majority of these religiosity exposures were measured in pregnancy, with the rest – approximately 10% – measured around 4 months after delivery (whether religiosity differs by whether the questionnaire was completed during or after pregnancy was also assessed).

**Table 1.** Summary of religious/spiritual beliefs and behaviours exposure variables used in this study. Sample sizes are 13,477 for mothers and 13,424 for partners.

Exposure		Mother, *N* (%)	Partner, *N* (%)
Belief in God/a divine power (variable *d810* for mothers; variable *pb150* for partners)	Yes	5932 (49.8%)	3456 (36.8%)
	No^a^	5977 (50.2%)	5944 (63.2%)
	Total	11,909	9400
Missing data		1568 (11.6%)	4024 (30.0%)

Religious affiliation (variable *d813* for mothers; variable *pb153* for partners)	Yes^b^	9969 (84.7%)	6869 (74.2%)
	None	1795 (15.3%)	2383 (25.8%)
	Total	11,764	9252
Missing data		1713 (12.7%)	4172 (31.1%)

Frequency of attendance at a place of worship (variable *d816* for mothers; variable *pb155* for partners)	Regular attendance^c^	1634 (14.0%)	935 (10.2%)
	Occasional attendance/never^d^	10,009 (86.0%)	8271 (89.8%)
	Total	11,643	9206
Missing data		1834 (13.6%)	4218 (31.4%)

a This ‘no’ coding includes both ‘no’ and ‘not sure’ responses. Of 5977 mothers coded as ‘no’, 1755 answered ‘no’ (29%) and 4222 answered ‘not sure’ (71%). Of 5944 partners coded as ‘no’, 2688 answered ‘no’ (45%) and 3256 answered ‘not sure’ (55%).

b This ‘yes’ coding is predominantly Christian (~95%), but also includes small numbers of other faiths/beliefs, including Jewish, Muslim, Sikh, Hindu and Buddhist, among others.

c ‘Regular attendance’ includes responses of ‘at least once a week’ or ‘at least once a month’. Of 1634 mothers coded as ‘Regular attendance’, 833 answered ‘at least once a week’ (51%) and 801 answered ‘at least once a month’ (49%). Of 935 partners coded as ‘Regular attendance’, 538 answered ‘at least once a week’ (57%) and 397 answered ‘at least once a month’ (43%).

d ‘Occasional/never’ includes responses of ‘at least once a year’ or ‘not at all’. Of 10,009 mothers coded as ‘occasional attendance/never’, 3438 answered ‘at least once a year’ (34%) and 6571 answered ‘not at all’ (66%). Of 8271 partners coded as ‘occasional attendance/never’, 2434 answered ‘at least once a year’ (29%) and 5837 answered ‘not at all’ (71%).

### Outcomes

To assess cooperation, we will use ‘blood donation’ as our outcome, coded as a binary yes/no variable (variable *d290* for mothers; variable *pa300* for partners). During pregnancy, mothers and partners were asked whether they had ever donated blood, providing a real-world measure of cooperative behaviour. All UK blood donations are voluntary, and donors are not compensated for their donation. Guidelines for UK blood transfusion services (https://www.transfusionguidelines.org/red-book), first published in 1990, state that donors must be aged between 17 and 65 years and appear healthy, with various exclusions, including certain long-term health conditions, recent infectious disease, taking medications and pregnancy.

One concern with this outcome is that it is a self-reported measure of cooperation, and therefore may be biased by factors such as social desirability. That is, people may over-report having given blood. This may be an issue, as measurement error can result in bias, the direction and magnitude of which depends on the structure of the measurement error (Hernán & Robins, 2020; Lash et al., 2021). If measurement error is non-differential and independent – i.e. only affects blood donation and is unrelated to other variables – then the bias is likely to be towards the null, meaning that any estimate is likely to under-report the true effect. However, if measurement error is differential – e.g. if religious individuals are more likely to say they donated blood than non-religious individuals – then this is likely to bias the religiosity–cooperation estimate upwards. Alternatively, if a social desirability effect exists and affects religiosity in addition to blood donation – say, some individuals are more likely to incorrectly report being both religious and having given blood – then this may result in additional bias, with social desirability acting as a source of residual confounding between religiosity and blood donation. In this latter scenario, the extent of this residual confounding necessary to alter the study's conclusions can be estimated, using the methods described above. Without a gold standard measure to validate against, methods to correct for measurement error can be difficult to apply (Innes et al., 2021); we therefore acknowledge this potential measurement error as a limitation of our study, and return to it when interpreting our results, but predominantly focus on methods for exploring confounding and selection bias. Despite this potential measurement error due to self-reported data collection, prior work in the Avon Longitudinal Study of Parents and Children has shown that self-reported measures of medical history and mental health – which may also be subject to social desirability bias – are comparable with ‘gold standard’ measures, such as medical records and clinical interviews (Golding et al., 2001). While this validation work did not involve measures of religiosity or blood donations, it does suggest that this questionnaire-based data collection may be broadly accurate and not strongly biased by social desirability.

### Confounders

**Figure 3.**
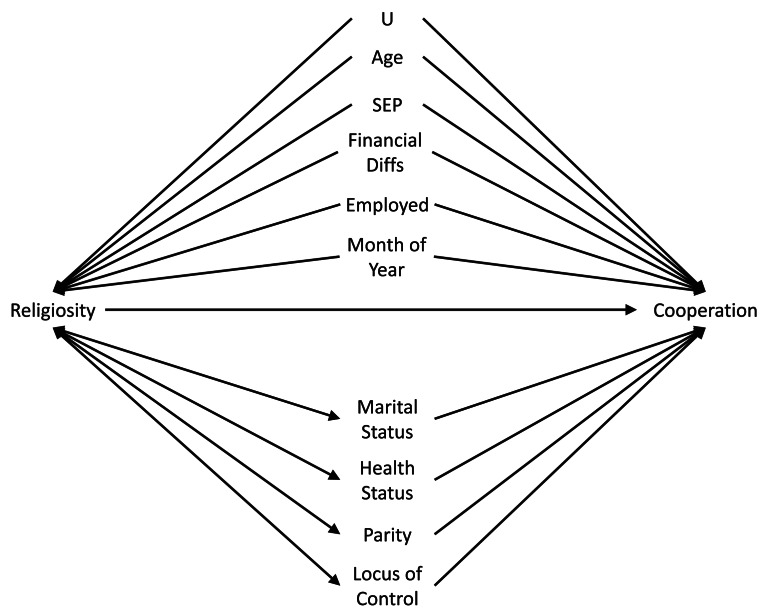
Causal graph encoding the assumptions made in Table 2 regarding confounding variables. Note that, for simplicity, hypothesised causal relations between covariates have not been displayed here. Presumed confounders are above the horizonal exposure–outcome arrow, while possible confounders/mediators with bidirectional causation between the exposure and covariate are below the horizontal arrow (and have bidirectional arrows between themselves and the exposure). The node ‘U’ denotes potential residual/unmeasured confounding.

As mentioned above, a confounder is a variable which causes both the exposure and the outcome. Given that we do not know with certainty which variables are confounders and which are mediators (and some may be both), some assumptions have to be made. A list of potential covariates included in the present study is provided in Table 2, along with justification for whether they are treated as a confounder, or treated as a confounder and/or mediator (summarised in Figure 3). The majority of these covariates are sociodemographic variables familiar to most readers; the only exception is ‘locus of control’, a psychological construct measuring the extent to which individuals believe outcomes of their behaviour are determined by themselves vs. external factors such as luck, fate or powerful others (Rotter, 1966), and has been associated with both religiosity (Iles-Caven et al., 2020) and cooperation (Boone et al., 1999).

**Table 2.** Details of covariates and whether they are assumed to be either confounders, or both confounders and mediators. For each causal path with the exposure (religiosity) and the outcome (blood donation/cooperation), we have coded as ‘no’, ‘unlikely’, ‘possibly’ or ‘yes’ depending on certainty of effect (these are qualitative judgment calls based on expert knowledge, existing literature, logical deduction or simply best guesses if no additional information was available). See the ‘Confounders’ sub-section of the Methods for additional justification and relevant literature regarding these decisions. Note that in some cases, due to a lack of data from the partners, we are using the mother's data as a proxy.

Covariate	Cohort (variable name)	Cause of exposure?	Caused by exposure?	Cause of outcome?	Caused by outcome?	Assignment	Justification
*Age* *^a^*	Mother (*mz028b*); partner (*partner_age*)	Yes	No	Possibly	No	Confounder	Impossible to be caused by religiosity or cooperation, so can only be a possible confounder
*Ethnicity*	Mother (*c800*); partner (*pb440*)	Yes	No	Possibly	No	Confounder	Impossible to be caused by religiosity or cooperation, so can only be a possible confounder
*Socio-economic position*	*Highest education:* mother (*c645*a); partner (*pb325*a) *Home ownership status:* mother and partner (*a006*) *Index of multiple deprivation:* Mother and partner (*jan1993imd2010q5_M*)	Yes	Unlikely	Yes	Unlikely	Confounder	Unlikely to be caused by religiosity or cooperation, so is a possible confounder
*Urban/rural status*	Mother and partner (*jan1993ur01ind_M*)	Possibly	Unlikely	Possibly	Unlikely	Confounder	Unlikely to be caused by religiosity or cooperation, so is a possible confounder
*Marital status*	Mother (*a525*); partner (*pa065*)	Possibly	Possibly	Possibly	Unlikely	Confounder/mediator	Unclear whether is a confounder or mediator (or both)
*Parity*	Mother and partner (*b032*)	Possibly	Possibly	Possibly	Unlikely	Confounder/mediator	Unclear whether is a confounder or mediator (or both)
*Locus of control*	Mother (*d842*); partner (p*a782*)	Possibly	Possibly	Possibly	Unlikely	Confounder/mediator	Unclear whether is a confounder or mediator (or both)
*Recent financial difficulties*	Mother (*b594*); partner (*pb184*)	Possibly	Unlikely	Possibly	Unlikely	Confounder	Unlikely to be caused by religiosity or cooperation, so is a possible confounder
*Employ-ment*	Mother (*c710*; *c711*; *c712*; *c713*); partner (*pb380*; *pb381*; *pb382*; *pb383*)	Possibly	Unlikely	Possibly	Unlikely	Confounder	Unlikely to be caused by religiosity or cooperation, so is a possible confounder
*Health status*	Mother (*b040*); partner (*a524*)	Possibly	Possibly	Possibly	Unlikely	Confounder/mediator	Unclear whether is a confounder or mediator (or both)
*Month of year* *^b^*	Mother (*d992*); partner (p*a902*)	Possibly	Unlikely	Possibly	Unlikely	Confounder	Unlikely to be caused by religiosity or cooperation, so is a possible confounder

a Note that technically these variables are ‘age at birth’ for mothers and partners. While it is possible that age at birth may also be caused by religiosity (e.g. religious norms prohibiting contraception or encouraging traditional gender roles (resulting in earlier pregnancy) and/or promoting sexual abstinence until marriage (delaying pregnancy)), this is unlikely to result in bias of the religiosity–cooperation/blood donation association. This is because cooperation/blood donation is the outcome in this analysis, and providing that the outcome does not also cause, or relate to, selection into the study (here, pregnancy), this will not result in bias, as it is unlikely that cooperation causes pregnancy; while many factors which potentially relate to both cooperation and pregnancy are already included as covariates in the analysis model (e.g. age, religiosity, socioeconomic position) and therefore adjusted for, we believe this assumption is likely to be valid. As such, we will refer to ‘age’ rather than ‘age at birth’ throughout this manuscript.

b Note that for mothers the blood donation and religiosity questions were taken from the same questionnaire, and therefore have the same month of completion. For partners the exposure and outcome were asked in separate questionnaires completed a few months apart; for partners, month of completion of the questionnaire containing the outcome (blood donation) will therefore be used here.

As discussed above, the decision of whether to code a covariate as a confounder or a confounder and/or mediator was a qualitative judgment based on expert knowledge, existing literature, logical deduction or simply best guesses (where no additional information was available). For instance, it is impossible for anything to cause age or ethnicity, meaning that they can only be possible confounders. As another example, we feel it is unlikely that religion or blood donation cause socioeconomic position to any great extent, while the reverse – socioeconomic position causing religion and blood donation – is more plausible (Gillum & Masters, 2010; Major-Smith et al., 2022; Schwadel, 2015), hence the assignment of socioeconomic position as a confounder. Similar considerations explain why urban/rural status, recent financial difficulties and employment status have been assigned as confounders. As there is a possibility that seasonal differences may impact how individuals respond to the religion and blood donation questions (reporting greater religiosity and cooperation at Christmastime, perhaps), month of questionnaire completion will also be included as a potential confounder. The remaining variables – marital status, parity, locus of control and health status – have been assigned as confounders and/or mediators because they may plausibly cause blood donation, but may also both cause and be caused by religiosity (or the causal relations are simply not known with any certainty; Iles-Caven et al., 2020; Koenig et al., 2012; Li, Okereke, et al., 2016; Li, Stampfer, et al., 2016; Shaver et al., 2020). We will refer to the full set of potential covariates in Table 2 as the ‘confounders and/or mediators’ scenario, and the smaller set of confounders, excluding potential confounders/mediators, as the ‘confounders only’ scenario. These are, of course, somewhat subjective – but hopefully plausible – assumptions regarding the confounding structure; needless to say, if these assumptions are incorrect (e.g. assuming that a variable is a confounder when in fact it is a mediator), this will introduce bias.

### Analysis

For all analyses, the basic model was a complete-case analysis logistic regression with religiosity as the exposure and blood donation as the outcome, both unadjusted and adjusted for confounders. This was repeated for each of the exposures (religious belief, affiliation and attendance), in both the mothers and partners separately. Given differences in data collection between mothers and partners – e.g. more detailed data collection for mothers, more missing partner data, and partners not being formally enrolled into the study – it was decided to analyse mothers and partners separately. Additionally, due to these differences, the selection pressures may vary between mothers and partners; performing multiple imputation and subsequent analyses separately in each cohort therefore allows the imputation model to vary and permits a different set of auxiliary variables for mothers and partners (see below). The point estimates and confidence intervals of the mother and partner models will be compared to assess whether similar associations are found in both sets of analyses, with formal comparisons conducted using post-estimation hypothesis testing.

However, there are several sources of bias which could result in the adjusted models providing biased causal estimates, as detailed in the introduction. These can be summarised as ‘mis-specified confounding model’, ‘residual confounding’ and ‘selection bias’; methods to explore these potential sources of bias will be discussed in turn below.

#### Mis-specified confounding model

As described above, it is not clear whether some of the covariates are confounders, mediators or both (e.g. marital status, health status, locus of control and parity; Table 2). As such, for the adjusted models we will perform these both with and without these covariates (‘confounders and/or mediators’ vs. ‘confounders only’ scenarios) to explore if/how results vary to these different assumptions regarding confounding. Assuming no other sources of bias, these two scenarios should bracket the minimum and maximum plausible causal estimates.

#### Residual confounding

To explore potential residual confounding, we will use both ‘*E*-values’ (from the ‘EValue’ R package; Mathur et al., 2018; VanderWeele & Ding, 2017) and ‘generalised sensitivity analysis’ (from the ‘gsa’ package in Stata; Harada, 2013) approaches as quantitative bias analyses. We will repeat these methods for each religiosity exposure in both mothers and partners, and for each of the ‘confounders only’ and ‘confounders and/or mediators’ scenarios.

Briefly, the *E*-value approach provides a single measure of association, on the risk ratio scale, between an unmeasured binary confounder and both the exposure and the outcome sufficient to remove an observed exposure–outcome association (although *E*-values can be generalised to non-binary and multiple unmeasured confounders; Ding & VanderWeele, 2016). For instance, an *E*-value of ‘2’ indicates that an unmeasured confounder which doubles the risk of both the exposure and the outcome would be required to reduce the observed effect to the null. *E*-values can also be used to assess the strength of unmeasured confounding required to alter an association so that the null is included in the confidence interval (i.e. so the effect is no longer ‘statistically significant’ at a given alpha level), or at any given effect size of interest.

The generalised sensitivity analysis approach is conceptually similar to an *E*-value, in that it estimates the association, using partial *R*^2^ or correlation coefficients, between an unmeasured confounder and both the exposure and outcome needed to remove or reduce the observed effect. Unlike the *E*-value approach, generalised sensitivity analysis relies on repeatedly generating an unobserved confounder, either continuous or binary, to evaluate the strength of these associations necessary to alter the observed exposure–outcome association. One benefit of this approach is that it is applied to individual-level data, and is therefore specific to the analysis model, allowing a range of different exposure and outcome variable types (e.g. linear model with a binary exposure, logistic model with a continuous exposure, etc.). In contrast, the *E*-value approach is based on summary-level data and is calculated on the risk ratio scale; while transformations can be applied for linear and logistic models, these are inflexible and rest on specific assumptions (VanderWeele & Ding, 2017). Generalised sensitivity analysis also allows one to ‘benchmark’ the strength of the hypothesised unmeasured confounder against observed confounders, facilitating interpretation as to whether this effect size may be reasonable. A limitation of this generalised sensitivity analysis approach is that it can be quite computationally intensive, and is currently only available in Stata, not in open-source software such as R (there are quantitative bias analysis packages in R which are similar to generalised sensitivity analysis (e.g. Carnegie et al., 2016; Cinelli & Hazlett, 2020), but currently these packages require continuous outcomes, rather than the binary outcomes used here; Kawabata et al., 2022).

These types of sensitivity analysis have received criticism, largely based on the fact that these methods only assume a single unmeasured confounder and therefore ignore associations and interactions with other covariates (Greenland, 2020). However, despite these issues, *E*-values, generalised sensitivity analysis and similar approaches which assume a single unmeasured confounder are often a good place to start when thinking about sensitivity analyses for residual confounding, as they at least attempt to assess this bias (rather than ignore it), are relatively intuitive, and can be performed in standard statistical software (VanderWeele, 2022; Vanderweele & Mathur, 2020). These simple approaches are also useful because the structure and magnitude of unmeasured confounding is often difficult, if not impossible, to estimate with certainty, meaning they provide an accessible way of exploring residual confounding without having to make numerous assumptions about unknown variables (VanderWeele, 2022). In the context of this paper, although we may expect some residual confounding – perhaps from social desirability bias, or personality measures which may cause both religiosity (Saroglou, 2002) and cooperation (Volk et al., 2011) – the structure and magnitude of this are largely unknowable; having a relatively simple, albeit imperfect, metric with which to assess the extent of residual confounding necessary to alter the study's conclusions is therefore of great value when assessing whether a causal interpretation may be warranted or not. Additionally, even though these approaches only assume a single unmeasured confounder, this could be considered as composite bias from multiple unmeasured confounders, so does not imply a single unmeasured confounder in reality (VanderWeele et al., 2021).

#### Selection bias

As this study uses data collected predominantly during pregnancy, the amount of missing data for individual questions in the mothers is relatively low (approximately 10–20%; although the amount of missing data for partners is much larger, at around 30–40%); however, even small amounts of missing data in individual variables can result in a much-reduced sample size (making analyses less efficient), while selection may still bias results.

**Figure 4.**
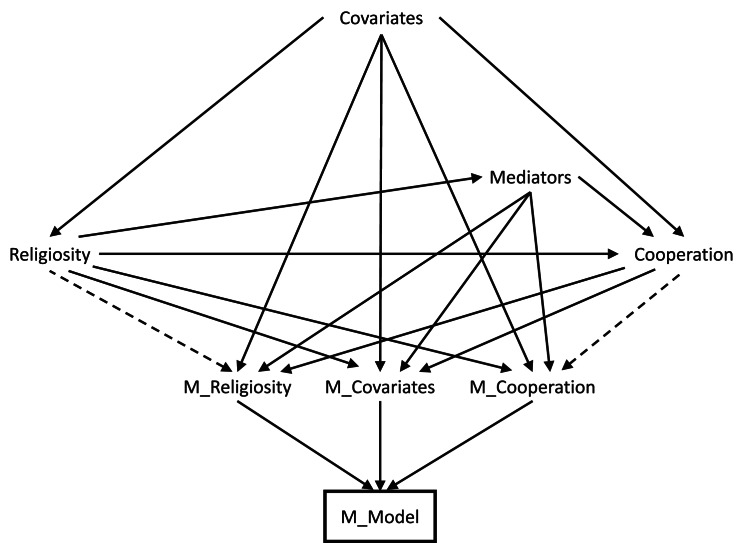
Assumed directed acyclic graph for missing data. Missingness markers for individual variables have been indicated with a prefix ‘M_’, while ‘M_Model’ denotes overall missingness in the complete-case analysis model (with a box around it, indicating that the complete-case analysis is conditional on this). For simplicity, all covariates in the substantive analysis model from Table 2 have been grouped together, as have the mediating variables of alcohol use, smoking and depression. Missing exposure data which may depend on the exposure (religiosity), and missing outcome data which may depend on the outcome (cooperation/blood donation), have been represented via dashed arrows. If this causal graph *excluding the dashed arrows* represents the true causal structure of the missing data, then multiple imputation with the mediators as auxiliary variables in the imputation model (in addition to the exposures, outcomes and covariates, plus other auxiliary variables (not displayed here) to predict missing data in Table 3) ought to meet the Missing-At-Random assumption, meaning that analyses using the imputed data may not be biased. However, if this causal graph *including the dashed arrows* represents the true causal structure of the missing data, then multiple imputation using all variables mentioned above will *not* meet the Missing-At-Random assumption, meaning that analyses using data imputed via standard multiple imputation will still be biased. This is because both the exposure and the outcome are associated with their own missingness. In this scenario, we can use further sensitivity analyses to explore how different Missing-Not-At-Random assumptions regarding the missing data of the exposure and outcome impact our conclusions.

Many of the covariates included here, such as age, ethnicity and socioeconomic position, are known to predict continued participation in the Avon Longitudinal Study of Parents and Children (Boyd et al., 2013; Cornish et al., 2020; Fernández-Sanlés et al., 2021; Fraser et al., 2013); inclusion of these covariates in the analysis model may reduce potential bias by making the Missing-At-Random assumption more plausible (i.e. that differences between missing and observed data can be explained by observed data). However, other variables not included in the analysis model because they may be mediators on the causal pathway between religiosity and cooperation may still result in selection bias if they also relate to missing data (e.g. smoking, alcohol use and depression/mental health). For these variables, we can try to make the Missing-At-Random assumption more plausible by conducting multiple imputations to impute this missing data (Lee et al., 2021; van Buuren, 2018; White et al., 2011) and including these mediators in the imputation model as auxiliary variables (Cornish et al., 2015; Hughes et al., 2019). We therefore conducted multiple imputation analyses, using smoking, alcohol intake and depression as auxiliary variables to try and satisfy the Missing-At-Random assumption (plus using additional auxiliary variables to help estimate other missing data; Table 3). The imputation models include all religiosity exposures, the blood donation outcome, all covariates in Table 2, and all auxiliary variables in Table 3 (with separate imputation for mothers and partners). A directed acyclic graph displaying these assumptions regarding missing data is given in Figure 4. We imputed to the full dataset, performing 50 imputations with a burn-in period of 10 (this was checked to ensure convergence), using the ‘mice’ package in R (van Buuren, 2018). Results from the imputed datasets were analysed using Rubin's rules.

**Table 3.** Details of auxiliary variables used for multiple imputation to impute missing data. All imputation models included the exposures, outcomes and confounders, in addition to the auxiliary variables detailed below. Note also that for partners, references to ‘mother’ refer to the study mother (i.e. the partner's partner), and not the partner's mother. As the partners have considerably more missing data, and there are a number of auxiliary variables that may provide information about this missing data, the list of auxiliary variables is much longer for partners compared with mothers.

Variable	Rationale for auxiliary variable
*Mothers’ auxiliary variables*	
Mother's depression in pregnancy (using Edinburgh Postnatal Depression Scale; *b371*)	Likely to be related to study participation (higher depression = greater drop-out), and potential mediator of exposure–outcome relationship
Mother's smoking status (*b650*; *b665*; *b667*)	Likely to be related to study participation (more smoking = greater drop-out), and potential mediator of exposure–outcome relationship
Mother's alcohol intake (in pregnancy: *b722*; before pregnancy: *b720*)	Likely to be related to study participation (higher alcohol intake = greater drop-out), and potential mediator of exposure–outcome relationship
Mother's occupational social class (*c755*)	Help estimate missing socioeconomic position confounder data
Household access to a car (*a053*)	Help estimate missing socioeconomic position confounder data
*Partners’ auxiliary variables*	
Partner's depression in pregnancy (using Edinburgh Postnatal Depression Scale; *pb260*)	Likely to be related to study participation (higher depression = greater drop-out), and potential mediator of exposure–outcome relationship
Partner's smoking status (*pb071*; *pb077*)	Likely to be related to study participation (more smoking = greater drop-out), and potential mediator of exposure–outcome relationship
Partner's alcohol intake (in past 3 months; *pb100*)	Likely to be related to study participation (higher alcohol intake = greater drop-out), and potential mediator of exposure–outcome relationship
Partner's occupational social class (*c765*)	Help estimate missing socioeconomic position confounder data
Household access to a car (*a053*)	Help estimate missing socioeconomic position confounder data
Mother's religiosity (*d810*; *d813*; *d816*)	Help estimate missing religiosity exposure data (on the assumption that partner's and mother's data will be correlated)
Mother's blood donation (*d290*)	Help estimate missing blood donation outcome data (on the assumption that partner's and mother's data will be correlated)
Mother's confounder data – age at birth (*mz028b*), ethnicity (*c800*), education (*c645*a), marital status (*a525*), locus of control (*d842*), financial difficulties (*b594*), employment (*c710*; *c711*; *c712*; *c713*) and health status (*b040*)	Help estimate missing confounder data (on the assumption that partner's and mother's data will be correlated)
Partner's education (*c666a*), ethnicity (*c801*) and employment (*c730*; *c731*; *c732*; *c733*) as reported by the mother	Help estimate missing education, ethnicity and employment data

Despite this, if the exposure and outcome cause their own missingness, then data will still be Missing-Not-At-Random (i.e. differences between missing and observed data *cannot* be explained by observed data) and results from the imputed data will still be biased. This may be plausible, as in a previous paper we found that religious attendance (although not necessarily religious belief or affiliation) was associated with continued study participation (Morgan et al., 2022), meaning that the exposure may cause missing data. Additionally, the outcome (blood donation) may also plausibly cause missing data, as cooperative individuals may be more likely to continue participating in longitudinal studies (continued participation being a measure of cooperation). As such, those with missing data may be more likely to be both non-religious and less cooperative (summarised using the dashed arrows between ‘Religiosity’ and ‘M_Religiosity’, and ‘Cooperation’ and ‘M_Cooperation’, respectively, in Figure 4). To explore this potential bias caused by exposure and outcome data being possibly Missing-Not-At-Random, we performed a series of Not-At-Random multiple imputation analyses (Lee et al., 2021; Tompsett et al., 2018). These methods allow one to vary the standard Missing-At-Random assumption of multiple imputation, by explicitly incorporating a bias parameter when imputing the missing data (e.g. if missing religiosity, can say that these individuals are less likely to be religious than predicted under a standard Missing-At-Random multiple imputation model).

As religious belief and affiliation were not independently associated with continued study participation, we have good reason to believe that these variables probably satisfy the Missing-At-Random assumption (Morgan et al., 2022); on the other hand, as religious attendance was associated with continued study participation, even after adjustment for multiple potential confounders, this variable may be Missing-Not-At-Random. We therefore focused these sensitivity analyses on religious attendance. Because we are unsure whether the data are Missing-At-Random or Missing-Not-At-Random, we performed a deterministic ‘tipping-point’ sensitivity analysis (Tompsett et al., 2018), where we varied the magnitude of the bias parameter to assess the extent of the Missing-Not-At-Random bias required to alter our original conclusions. As religious attendance and/or cooperation may be associated with increased study participation, we focused on scenarios where those with missing data were less likely to attend a place of worship and/or less likely to donate blood. We performed three sets of Not-At-Random multiple imputation sensitivity analyses: (i) performing Not-At-Random multiple imputation on religious attendance only, using a range of bias parameters (on the log-odds scale from 0 to −2, in steps of 0.25; blood donation will be imputed as per standard Missing-At-Random multiple imputation); (ii) performing Not-At-Random multiple imputation on blood donation/cooperation only, using a range of bias parameters (on the log-odds scale from 0 to −2, in steps of 0.25; religious attendance will be imputed as per standard Missing-At-Random multiple imputation); and (iii) combining (i) and (ii) (i.e. Not-At-Random multiple imputation sensitivity analysis on both religious attendance and blood donation simultaneously, using the same range of bias parameters as above). These bias parameters – known as ‘conditional sensitivity parameters’ (Tompsett et al., 2018) – denote the log-odds difference between those with vs. without observed data, conditional on all other variables in the imputation model; as they are not particularly intuitive to understand, we will convert these to marginal sensitivity parameters (the unadjusted difference between those with vs. without observed data) and associated prevalence estimates to facilitate comprehension of the effect sizes involved and assess whether the bias needed to alter the study's conclusions is reasonable or not. As with standard multiple imputation, we generated 50 imputed datasets per bias parameter, with a burn-in period of 10 iterations.

#### Summary of analyses

These analyses are by no means exhaustive, but are intended to illustrate how sensitivity analyses can be used to aid observational research with the explicit aim of causal inference. Hopefully these analyses will provide some indication as to how robust these results are to confounder mis-specification, residual confounding and selection bias, and whether we can have confidence that our results reflect a true causal estimate. A table of all the analyses is detailed below (Table 4). While all these sensitivity analyses may seem over-the-top – and perhaps are! – hopefully it highlights some of complications when trying to estimate causal effects from messy, real-world, observational data. All analyses were conducted in R 4.0.4 (R Development Core Team, 2021), other than the generalised sensitivity analysis which was conducted in Stata v.17.

**Table 4.** Summary of analyses. Where appropriate, all quantitative bias analyses (*E*-value, generalised sensitivity analysis, multiple imputation and Not-At-Random multiple imputation) were repeated using both ‘confounders only’ and ‘confounders and/or mediators’ adjusted models (see Table 2). All analyses were repeated for mothers and partners.

Method	Rationale	Exposure(s)	Outcome
Unadjusted complete-case analysis	Unadjusted exposure–outcome associations	Religious belief, affiliation and attendance	Blood donation
Adjusted complete-case analysis (adjusted for confounders only)	Exposure–outcome estimate adjusting for a reduced range of potential confounders (excluding some that may be confounders and/or mediators)	Religious belief, affiliation and attendance	Blood donation
Adjusted complete-case analysis (adjusted for confounders and/or mediators)	Exposure–outcome estimate adjusting for full range of potential confounders (including some that may also be mediators)	Religious belief, affiliation and attendance	Blood donation
*E*-value	Explore potential bias due to residual confounding	Religious belief, affiliation and attendance	Blood donation
Generalised sensitivity analysis	Explore potential bias due to residual confounding	Religious belief, affiliation and attendance	Blood donation
Multiple imputation	Explore potential bias due to selection (assuming imputed data is Missing-At-Random)	Religious belief, affiliation and attendance	Blood donation
Not-At-Random multiple imputation tipping point analysis on exposure, outcome, and exposure and outcome together	Explore potential bias due to selection (assuming data is Missing-Not-At-Random for exposure and/or outcome)	Religious attendance (as religious belief and affiliation assumed to be Missing-At-Random)	Blood donation

## Results

Of 11,870 mothers with observed blood donation data (1569 (11.6%) missing), 3554 reported previously donating blood (29.8%). Rates were slightly higher among partners, with 2783 of 8129 (34.2%) partners with observed data having donated blood (5295 (39.4%) missing). Descriptive statistics for the religiosity exposures are provided in Table 1. Most mothers had a religious affiliation (84.7%) and half believed in God/a divine power (49.8%), while fewer regularly attended a place of worship (14.0%). Consistent with previous research (Voas et al., 2013), levels of religiosity were lower among partners (religious affiliation = 74.2%; religious belief = 36.8%; regular religious attendance = 10.2%). Full descriptive statistics for covariates and auxiliary variables are provided in Tables S1 (for mothers) and S2 (for partners) of the supplementary information. As completion of the questionnaire after delivery was associated with both religious attendance and blood donations among mothers, we decided to include this variable in all mother-based models to account for this potential confounding (Table S3; no such association was reported for partners; Table S4).

Cross-tabulation of the religiosity exposures by the outcome for mothers indicated little association between religious affiliation and blood donation, a 4 percentage-point increase in blood donation among religious believers and a 10 percentage-point increase among those who attended a place of worship regularly (Table S5). Comparable results were observed for partners (Table S6).

**Figure 5.**
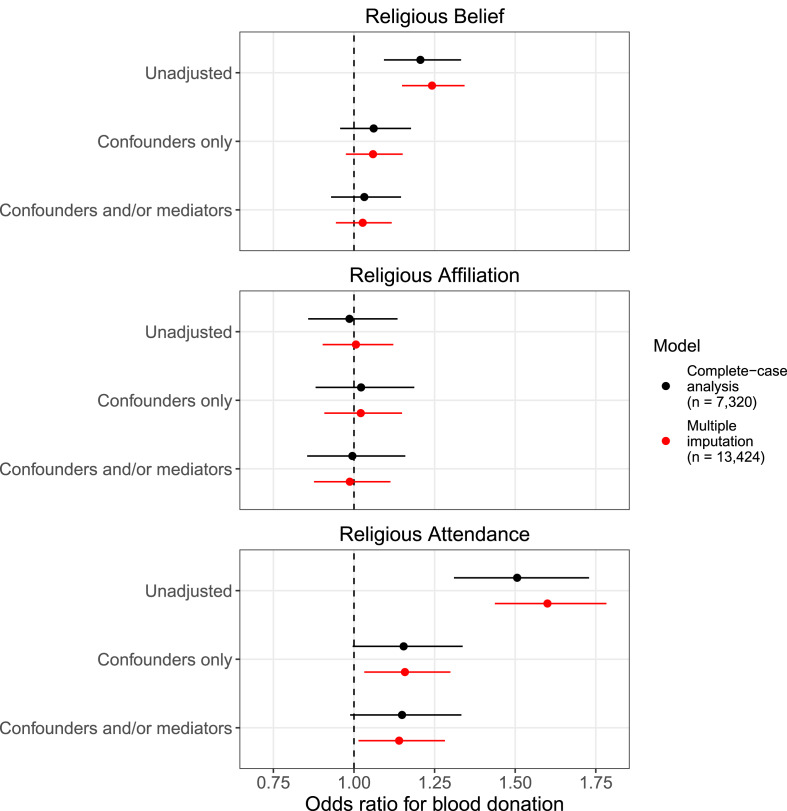
Results of the complete-case (black) and multiple imputation (red) analyses for each religiosity exposure with blood donation as the outcome for mothers. The ‘confounders only’ scenario adjusts only for assumed confounders, while the ‘confounders and/or mediators’ scenario adjusts for both assumed confounders and variables which may be both confounders and mediators (see Table 2 and Figure 3). For differences in the probabilities of donating blood based on these models, see Figure S1.

Complete-case analysis results for each exposure, comparing unadjusted, ‘confounder only’-adjusted and ‘confounder and/or mediator’-adjusted models for mothers are displayed in Figure 5 (full results in Table S7). In the adjusted models, there was little evidence that either religious belief or religious affiliation was associated with blood donation. In contrast, there was a positive association between regular religious attendance and having donated blood, although the 95% confidence interval crossed the null (confounder only adjusted odds ratio = 1.154, 95% confidence interval = 0.996 to 1.336, *p* = 0.0563). This corresponds to a 2.9 percentage-point increase in the probability of donating blood (95% confidence interval = −0.1 to 5.9%; Figure S1). For all exposures, the ‘confounders only’ and ‘confounders and/or mediators’ models produced very similar results, suggesting that bias due to mis-specified confounding may be minimal. Results were comparable for partners, but with a more pronounced association for religious attendance (confounder only adjusted odds ratio = 1.498, 95% confidence interval = 1.240 to 1.809, *p* < 0.0001; Figure S2 and Table S8), corresponding to a 9.1 percentage-point increase in the probability of donating blood (95% confidence interval = 4.7 to 13.5%; Figure S3). A post-estimation hypothesis test provided strong evidence that the partner's coefficient differed from that of the mothers (*p* = 0.0066).

Given the lack of evidence for an adjusted association with religious belief and affiliation, and that the ‘confounders only’ and ‘confounders and/or mediators’ models were so similar, we will focus the quantitative bias analysis for residual confounding on religious attendance in the ‘confounders only’ scenario. Taking mothers first, the *E*-value to reduce the observed odds ratio of 1.154 to null was 1.36. This means that an unmeasured confounder which increased the risk of both the exposure (religious attendance) and outcome (blood donation) by 36% could fully explain away the observed association. To help contextualise these results, the *E*-value to reduce the unadjusted observed odds ratio (1.506) to the adjusted odds ratio (1.154) was 1.55, which is larger than the above *E*-value for shifting the adjusted odds ratio to the null. Compared with the change from the unadjusted to adjusted results, only a relatively small amount of residual confounding is necessary to produce a null association from the adjusted model. Our confidence that this reflects a true positive causal relationship between religious attendance and blood donation may therefore be quite weak. The results of the generalised sensitivity analysis were comparable (see Figure S4 for more details and interpretation). For partners, the *E*-value to reduce the observed odds ratio of 1.498 to null was 1.75, meaning that a greater degree of unmeasured confounding, relative to mothers, is necessary to explain away this association; reducing the observed association so that it is no longer ‘statistically significant’ (based on the lower 95% confidence interval of the odds ratio; 1.240) would require an unmeasured confounder to increase the risk of the exposure and outcome by approximately 50% (see Figures S5 and S6 for the partners generalised sensitivity analysis).

We next turn to the results of the multiple imputation analyses, which attempt to remove selection bias due to missing data. Using standard multiple imputation, which assumes data are Missing-At-Random, there is little difference in the imputed results compared to those of the complete-case analyses for mothers (Figure 5; although the confidence intervals are narrower, as multiple imputation makes use of all the available information and so is more efficient). Imputed results were similar for partners, although for the religious attendance exposure the imputed analyses were slightly closer to the null (confounder only adjusted odds ratio = 1.367, 95% confidence interval = 1.172 to 1.594, *p* < 0.0001), potentially suggesting that the complete-case estimates may have been slightly biased upwards by selection (Figure S2). Using these estimates from the partner's imputed data, the *E*-values are correspondingly smaller compared with those of the complete-case analysis: an *E*-value of 1.61 to observe a null association, and an *E*-value of 1.38 to make the association no longer ‘statistically significant’ at a 0.05 alpha level.

Finally, we report results using the Not-At-Random multiple imputation approach, which assumes that the religious attendance exposure and/or blood donation outcome are Missing-Not-At-Random, with participants who do not regularly attend a place of worship or donate blood more likely to have missing data. For both mothers and partners, if either the exposure or outcome by itself is Missing-Not-At-Random, then there is little difference in the results. However, if the exposure and outcome are both Missing-Not-At-Random, then greater selection in both results in a larger exposure–outcome association (Figures S7–S12, and Tables S9–S14); this suggests that, if both religious attendance and blood donation are Missing-Not-At-Random, the true effect may be larger than that observed in the complete-case and the standard multiple imputation analyses. Note also that, contrary to the recommendations given in Tompsett et al. (2018), we did not include missingness indicators for each variable with missing data in the imputation models. This is because many of these indicators were highly collinear, and their inclusion was found to produce implausible results; for example, those with missing religious attendance data were imputed as *more* likely to regularly attendance a place of worship, with this bias stronger for partners compared with mothers. This is implausible because, based on previous research, participants with missing data were *less* likely to attend a place of worship (Morgan et al., 2022). Furthermore, in the standard multiple imputation analyses – which did not include these missingness markers – participants with missing data were imputed as being less likely to attend a place of worship, as expected. Removing these missingness indicators in the Not-At-Random multiple imputation analyses resulted in more plausible imputations where those with missing religious attendance data were more likely to be imputed as not attending (consistent with expectations and the standard multiple imputation results).

## Discussion

In this paper we have aimed to show how sensitivity analyses can be applied to assess whether a causal interpretation from observational data may be warranted, using religion and blood donation as a motivating example. We observed no association between religious belief or affiliation and blood donation when adjusting for relevant confounders, suggesting that a causal relationship is unlikely. There was stronger evidence for an association between religious attendance and blood donation, especially among partners. The analyses above suggest that, although selection bias is unlikely to alter these conclusions, unmeasured confounding is a greater threat to a causal interpretation, especially as factors known to potentially cause both religiosity and cooperation (e.g. personality; Saroglou, 2002; Volk et al., 2011) could not be assessed here. While much uncertainty undoubtedly remains, these results are suggestive – albeit rather weak – evidence for a potential causal relationship between regular religious attendance and higher rates of blood donation.

Few differences between the ‘confounders only’ and ‘confounders and/or mediators’ models were observed. This indicates that the covariates in the ‘confounders only’ scenario were sufficient to account for much of the confounding explained by observed covariates, and that the additional variables in the ‘confounders and/or mediators’ scenario were unlikely to strongly confound or mediate the religion–blood donation relationship. Overall, the results of the complete-case analyses were similar to those of the standard multiple imputation analyses, suggesting that either there was little bias due to selection in the complete-case analyses, or that imputation was not sufficient to remove bias; from these results it is impossible to tell between these alternatives. The Not-At-Random multiple imputation analyses aimed to explore this possibility, finding that plausible patterns of selection – i.e. participants who did not regularly attend a place of worship or donate blood being more likely to have missing data – did not alter this interpretation; indeed, given these likely patterns of selection any such bias may lead to an *under*-estimate of the true causal effect. This occurs because both the exposure and outcome are negatively associated with selection, which results in a reduction of the effect estimate in the selected sample. From the data available it is impossible to tell which is the true selection mechanism, but it is reassuring that these conclusions are robust to a range of plausible selection scenarios. While these different confounding assumptions and approaches to overcome selection bias did not greatly alter the conclusions of complete-case analyses here, this cannot be assumed for all studies and must be explored on a case-by-case basis.

Overall, these results provide some support for the idea that religion may promote cooperation (Galen, 2012; Purzycki et al., 2016; Schulz et al., 2019), although perhaps only among highly religious individuals who regularly attend a place of worship. Other research has reported similar associations, with religious attendance having stronger associations with health and behaviours than religious belief or identity (VanderWeele, 2017a, 2017b). The mechanisms remain unclear, but potentially include religious attendance enhancing social support and promoting religious norms and values (VanderWeele, 2017b); similar mechanisms may also promote cooperation and blood donation. A more prosaic explanation is that temporary community-based blood donation venues in the UK sometimes occur in religious buildings (https://www.blood.co.uk/the-donation-process/about-our-donation-venues/), so are more visible and accessible to those who attend religious services. If this religious attendance association is causal, understanding the mediators underlying this relationship is an important topic for future research.

The stronger association among partners is more surprising. If this reflects a true causal effect, this suggests that religious attendance may have a greater impact on cooperative behaviour in men compared with women. Alternatively, perhaps this gender difference in cooperation by religiosity is specific to blood donation, and not cooperation more broadly. This difference could also have a non-causal explanation; religious men may be more likely to claim they had donated blood to project a positive image, compared with women, for instance. These potential explanations remain speculative, and further research is required to assess whether this result replicates and the reason(s) for this gender difference.

There are many strengths of this study, including the sample being both largely representative of the target population (i.e. pregnant women and their partners in Bristol, UK), and with detailed information on both religious exposures and multiple potential confounders. However, these data also possess many limitations which pose a threat to both causal inference and generalisability. First, as mentioned in the Methods, there is the possibility of measurement error biasing these results. For instance, if the true association between religious attendance and blood donation was null, the observed positive relationship could be explained if individuals who regularly attend a place of worship were more likely to claim they donated blood. Given the lack of association with both religious belief and affiliation and blood donation, it is unlikely that this type of differential measurement error applies to religious individuals as a whole; otherwise, to explain the null association observed here the true association would have to be negative, which is probably rather implausible. However, we cannot rule out this measurement bias applying to religious attendance specifically. One method to explore this possibility would be to use exposure and outcome data from different sources, for instance, comparing self-reported religious attendance with a more objective or behavioural measure of cooperation. Indeed, recent work has shown that those who attended a place of worship more frequently were more likely to continue participating in this study (Morgan et al., 2022); this is a more objective measure of cooperation which corroborates the results above.

A further potential limitation is that perhaps blood donation may not have been the optimal measure of cooperation. Much work on the cultural evolution of religion suggests that religion may have evolved to foster large-scale cooperation among co-religionists (Lang et al., 2019; Purzycki et al., 2016; Richerson et al., 2016), yet donating blood is a public good which could theoretically benefit anyone in society, regardless of their religious background. Perhaps a design in which cooperation was directed specifically towards co-religionists would find stronger associations (although see Pasek et al., 2023). Alternatively, research has suggested that religious individuals may have a greater disgust response (Yu et al., 2022), potentially reflecting concerns over purity and contamination, which may deter them from donating blood (which involves needles, potential perceived risks of contamination, etc.). This may counteract any potential positive effects of religiosity promoting cooperation.

Third, despite coming from a longitudinal study, the data here are predominantly cross-sectional, with variables measured from questionnaires during pregnancy. This is because the outcome blood donation was only asked once in this study. To provide greater evidence for causality, and rule out any potential reverse causation – between both the exposures and the outcome, and with the covariates – longitudinal data with repeated exposure, outcome and confounder data are needed (VanderWeele, 2021; VanderWeele et al., 2016). While sensitivity analyses can help provide stronger evidence for a causal effect from imperfect observational data, as they rest on various additional assumptions (which may not be met) they are no replacement for research using better study designs; as always, prevention is better than cure.

Additionally, given that this is a Western, industrialised, and largely Christian population, we acknowledge that these findings may not be generalisable beyond this cultural, social and religious context; while these results may help shed some light on the function of religion in large-scale Western societies, given the cross-cultural variability in religious beliefs, behaviours and experiences, particularly in smaller-scale societies (Peoples et al., 2016; Purzycki et al., 2016; Wright, 2010), these results should not be taken as a universal feature of human societies without further exploration and replication. Relatedly, as the sample consists of pregnant mothers and their partners, it is unclear how generalisable these results are to the wider UK adult population.

Finally, while the focus of this paper has been on causal inference, we stress that a single study is insufficient to prove causality. A ‘triangulation’ approach is necessary for this, in which findings are replicated and corroborated using a range of study designs, each with different assumptions and biases regarding causal inferences (Lawlor et al., 2016). For instance, instrumental variable, observational and negative control studies all rest on different assumptions and have different sources of bias, but if all approaches provide similar conclusions, then we can have greater confidence in claiming a causal effect. Importantly, this goes beyond mere replication, as the same study designs may have similar sources of bias (Munafò & Davey Smith, 2018). In its own small way, it is hoped that this paper contributes to this growing knowledge base.

In conclusion, although causal inferences from observational data are difficult and rest on many untestable assumptions, sensitivity analyses can be used to explore these assumptions and assess whether a causal interpretation may be plausible. This study has demonstrated how to apply these methods, with example code and synthetic data available to help readers implement and understand these approaches. As hopefully this paper has shown, causal inference from observational data can be messy and rather complicated, and often lots of doubt still remains as to whether an effect is causal or not, but making our research aims and assumptions clear – and interrogating these assumptions – is an important step towards improving our scientific inferences.

## Research transparency and reproducibility

Access to the Avon Longitudinal Study of Parents and Children data is through a system of managed open access. Information about access to this data is given on the study website (http://www.bristol.ac.uk/alspac/researchers/access/) and in the data management plan (http://www.bristol.ac.uk/alspac/researchers/data-access/documents/alspac-data-management-plan.pdf). Data used for this submission will be made available on request to the Executive (alspac-exec@bristol.ac.uk). The datasets presented in this article are linked to the Avon Longitudinal Study of Parents and Children project number B4030; please quote this project number during your application.

Example analysis code to explore and test the different methods presented in the paper, as well as formal analysis scripts and synthetic datasets (created using the ‘synthpop’ R package; Nowok et al., 2016), are openly available on DM-S's GitHub page: https://github.com/djsmith-90/AnalysisCode_BloodDonation_B4030. As raw study data cannot be released, these synthesised datasets are modelled on the original data, thus maintaining variable distributions and relations among variables (albeit not perfectly), while at the same time preserving participant anonymity and confidentiality. Please note that while these synthetic datasets can be used to follow the analysis scripts associated with this paper, as data are simulated they should *not* be used for research purposes.

## Supplementary material

For supplementary material accompanying this paper visit https://doi.org/10.1017/ehs.2023.17. click here to view supplementary material
