# Supplementary material for: Exploring causality from observational data: An example assessing whether religiosity promotes cooperation
Source: Evol Hum Sci. 2023 Jun 27;5:e22. doi: 10.1017/ehs.2023.17 (PMC10426067; doi:10.1017/ehs.2023.17)

Supplementary Material for 'Exploring causality from observational data: An example assessing whether religiosity promotes cooperation'

Section S1: Differences from Registered Analysis Plan (https://osf.io/z5gcm/)

Overall, the registered analysis plan was followed as specified, with only minor changes, as detailed below. None of these changes materially alter either the research question or the analysis methods described in the analysis plan.

A full breakdown of the changes compared to the registered analysis plan is as follows:

- Table 1 has been updated to include the descriptive statistics of the religiosity exposures.
- In table 2, the partner's month of questionnaire completion has been updated from 'pa902' to 'pb902', to correspond to the time-point at which the blood donation outcome was asked.
- In table 3, the mother's religious attendance variable was incorrectly written as 'd815'; this has now been updated to 'd816'. Similarly, the mother's pre-pregnancy alcohol intake variable should have been 'b720', not 'b370' as specified in the analysis plan.
- For mother's alcohol intake during pregnancy, previously we planned to use variable 'c373', but after inspecting this variable it was found to contain a large proportion of missing data. We therefore used variable 'b722' instead, which also measures alcohol intake during pregnancy, but with considerably less missing data.
- Since submitting the analysis plan, new partner's data has been made available (see Northstone et al., 2023 citation in the main text), including a new variable providing the partner's age at delivery ('partner_age'). This variable has been used instead of the previously specified variable 'pb910', which gives the partner's age at completion of the questionnaire containing the religion questions. This new variable contains less missing data than the original 'pb910' variable, and also means that mother and partner ages now both measure age at delivery.
- In the analysis plan, we referred to the set of covariates which contains only assumed confounders as the 'reduced-confounders' scenario, and the set of covariates including both assumed confounders and potential confounders and/or mediators as the 'full-confounders' scenario. In the full Registered Report we have referred to these as the 'confounders only' and 'confounders and/or mediators' scenarios, respectively, as we believe that this improves clarity.
- In the analysis plan, we said that "We will compare the point estimates and confidence intervals of the mother and partner models to assess whether similar associations are found in both sets of analyses". In the full Registered Report we have also added in post-estimation hypothesis tests to formally compare whether the mother and partner results differ.
- In the analysis plan, for the quantitative bias analyses to explore potential unmeasured confounding we said that "We will repeat these methods for each religiosity exposure in both mothers and partners, and for each of the 'full-confounders' ['confounders only'] and 'reduced-confounders' ['confounders and/or mediators'] scenarios.". As detailed in the results section, as there was no association between religious belief or affiliation and blood donation, in either mothers or partners, we did not perform these bias analyses for either of these exposures (because there was no association to explain away by unmeasured confounding). Likewise, as the results of the 'confounders only' and 'confounders and/or mediators' scenario were so similar, we decided to focus these analyses on the 'confounder only' scenario, as the 'confounder and or/mediators' scenario would produce largely identical results.

Table S1: Descriptive statistics of covariates and auxiliary variables for mother's data (*n* = 13,477). N (%) for categorical variables; mean (SD) for continuous variables.

Variable	Response	N (%) or mean (SD)
**Age at birth (years; range = 17-43)**		28.1 (4.85)
Missing data		4 (0.03%)
**Ethnicity**	*White*	11,589 (97.4%)
	Other than white	305 (2.6%)
	Total	11,894
Missing data		1,583 (11.7%)
**Highest educational qualification (for a comparison of different UK educational qualifications, see: https://www.gov.uk/what-different-qualification-levels-mean/list-of-qualification-levels: for more information about the Certificate of Secondary Education, see: https://en.wikipedia.org/wiki/Certificate_of_Secondary_Education)**	*Certificate of Secondary Education/None*	2,369 (19.8%)
	Vocational qualification	1,179 (9.8%)
	O-level	4,169 (34.8%)
	A-level	2,710 (22.6%)
	Degree or higher	1,561 (13.0%)
	Total	11,991
Missing data		1,486 (11.0%)
**Home ownership status**	*Owned/Mortgaged*	9,285 (73.8%)
	Rented	901 (7.2%)
	Council/Housing association	1,971 (15.7%)
	Other	430 (3.4%)
	Total	12,588
Missing data		889 (6.6%)
**Area-level index of multiple deprivation (quintiles)**	*Quintile 1 (least deprived)*	3,112 (25.2%)
	Quintile 2	2,671 (21.6%)
	Quintile 3	2,162 (17.5%)
	Quintile 4	2,401 (19.4%)
	Quintile 5 (most deprived)	2,028 (16.4%)
	Total	12,385
Missing data		1,091 (8.1%)
**Urban/rural location**	*Urban (population > 10,000)*	11,190 (90.1%)
	Rural (town, village or hamlet)	1,225 (9.9%)
	Total	12,415
Missing data		1,062 (7.9%)
**Employment status**	*Not employed*	7,362 (61.1%)
	Employed	4,694 (38.9%)
	Total	12,056
Missing data		1,421 (10.5%)
**Marital status**	*Currently married*	9,547 (75.5%)

Variable	Response	N (%) or mean (SD)
	Never married	2,326 (18.4%)
	Separated/Widowed/Divorced	767 (6.1%)
	Total	12,640
Missing data		837 (6.2%)
**Parity**	*0*	5,515 (44.2%)
	1	4,423 (35.4%)
	2 or more	2,548 (20.4%)
	Total	12,486
Missing data		991 (7.4%)
**Locus of control (range = 0-12; higher scores indicate a more external locus of control)**		4.35 (2.16)
Missing data		3,286 (24.4%)
**Recent financial difficulties**	*No*	9,966 (86.5%)
	Yes	1,551 (13.5%)
	Total	11,517
Missing data		1,960 (14.5%)
**Self-reported health status**	*Always well*	3,705 (32.0%)
	Usually well	6,986 (60.2%)
	Often/Sometimes/Always unwell	907 (7.8%)
	Total	11,598
Missing data		1,879 (13.9%)
**Month of questionnaire completion (for blood donation outcome question)**	*January*	1,546 (12.9%)
	February	942 (7.9%)
	March	868 (7.3%)
	April	557 (4.7%)
	May	1,123 (9.4%)
	June	1,402 (11.7%)
	July	1,291 (10.8%)
	August	971 (8.1%)
	September	961 (8.0%)
	October	961 (8.0%)
	November	904 (7.6%)
	December	429 (3.6%)
	Total	11,955
Missing data		1,522 (11.3%)
**Questionnaire containing religion questions completed after delivery**	*No*	10,311 (88.3%)
	Yes	1,371 (11.7%)
	Total	11,682
Missing data		1,795 (13.3%)
**Depression score (Edinburgh post-natal depression score; range = 0-30; higher scores indicate more depressive symptoms)**		6.96 (4.83)

**Variable**	**Response**	**N (%) or mean (SD)**
Missing data		1,737 (12.9%)
**Ever smoked**	*No*	6,223 (49.4%)
	Yes	6,377 (50.6%)
	Total	12,600
Missing data		877 (6.5%)
**Smoked during pregnancy**	*No*	9,432 (74.3%)
	Yes	3,263 (25.7%)
	Total	12,695
Missing data		782 (5.8%)
**Alcohol intake pre-pregnancy**	*Never*	1,042 (8.3%)
	Less than 1 glass per week	4,717 (37.5%)
	1 or more glasses per week	5,414 (43.0%)
	1 or more glasses per day	1,411 (11.2%)
	Total	12,584
Missing data		893 (6.6%)
**Alcohol intake during pregnancy**	*Never*	6,197 (50.3%)
	Less than 1 glass per week	4,271 (34.7%)
	1 or more glasses per week	1,677 (13.6%)
	1 or more glasses per day	170 (1.4%)
	Total	12,315
Missing data		1,162 (8.6%)
**Occupational social class (for more information on these social classes, see: https://sru.soc.surrey.ac.uk/SRU9.html**	*I (Professional)*	577 (5.9%)
	II (Managerial and technical)	3,087 (31.6%)
	III Non-manual (Skilled)	4,171 (42.7%)
	III Manual (Skilled)	766 (7.9%)
	IV/V (Partly-skilled/unskilled)	1,156 (11.8%)
	Total	9,757
Missing data		3,720 (27.6%)
**Household access to car**	*No*	1,297 (10.3%)
	Yes	11,297 (89.7%)
	Total	12,594
Missing data		883 (6.6%)

Table S2: Descriptive statistics of covariates and auxiliary variables for partner's data (*n* = 13,424). Note that references to 'mother' refer to the study mother (i.e., the partner's partner), and not the partner's mother. N (%) for categorical variables; mean (SD) for continuous variables.

**Variable**	**Response**	**N (%) or mean (SD)**
**Age at birth (years; range = 17-55)**		30.7 (5.70)
Missing data		2,333 (17.4%)
**Ethnicity**	*White*	9,117 (97.1%)
	Other than white	271 (2.9%)
	Total	9,388
Missing data		4,036 (30.1%)
**Highest educational qualification (for a comparison of different UK educational qualifications, see: https://www.gov.uk/what-different-qualification-levels-mean/list-of-qualification-levels: for more information about the Certificate of Secondary Education, see: https://en.wikipedia.org/wiki/Certificate_of_Secondary_Education)**	*Certificate of Secondary Education/None*	2,027 (21.5%)
	Vocational qualification	780 (8.3%)
	O-level	2,116 (22.4%)
	A-level	2,648 (28.0%)
	Degree or higher	1,870 (19.8%)
	Total	9,441
Missing data		3,983 (29.7%)
**Home ownership status**	*Owned/Mortgaged*	9,259 (73.8%)
	Rented	898 (7.2%)
	Council/Housing association	1,956 (15.6%)
	Other	427 (3.4%)
	Total	12,540
Missing data		884 (6.6%)
**Area-level index of multiple deprivation (quintiles)**	*Quintile 1 (least deprived)*	3,116 (25.3%)
	Quintile 2	2,663 (21.6%)
	Quintile 3	2,155 (17.5%)
	Quintile 4	2,387 (19.4%)
	Quintile 5 (most deprived)	2,014 (16.3%)
	Total	12,335
Missing data		1,089 (8.1%)
**Urban/rural location**	*Urban (population > 10,000)*	11,141 (90.1%)
	Rural (town, village or hamlet)	1,224 (9.9%)
	Total	12,365
Missing data		1,059 (7.9%)
**Employment status**	*Not employed*	1,202 (12.5%)
	Employed	8,380 (87.5%)
	Total	9,582
Missing data		3,842 (28.6%)

Variable	Response	N (%) or mean (SD)
**Marital status**	*Currently married*	6,753 (82.3%)
	Never married	1,087 (13.3%)
	Separated/Widowed/Divorced	362 (4.4%)
	Total	8,202
Missing data		5,222 (38.9%)
**Mother's parity**	*0*	5,488 (44.1%)
	1	4,413 (35.5%)
	2 or more	2,538 (20.4%)
	Total	12,439
Missing data		985 (7.3%)
**Locus of control (range = 0-11; higher scores indicate a more external locus of control)**		3.78 (2.29)
Missing data		6,322 (47.1%)
**Recent financial difficulties**	*No*	7,860 (86.1%)
	Yes	1,264 (13.9%)
	Total	9,124
Missing data		4,300 (32.0%)
**Health status (as reported by mother)**	*Always well*	5,735 (46.8%)
	Usually well	5,997 (48.9%)
	Often/Sometimes/Always unwell	523 (4.3%)
	Total	12,255
Missing data		1,169 (8.7%)
**Month of questionnaire completion (for blood donation outcome question)**	*January*	1,038 (12.6%)
	February	664 (8.0%)
	March	664 (8.0%)
	April	434 (5.3%)
	May	706 (8.5%)
	June	960 (11.6%)
	July	877 (10.6%)
	August	692 (8.4%)
	September	629 (7.6%)
	October	645 (7.8%)
	November	627 (7.6%)
	December	329 (4.0%)
	Total	8,265
Missing data		5,159 (38.4%)
**Questionnaire containing religion questions completed after delivery**	*No*	8,984 (96.9%)
	Yes	286 (3.1%)
	Total	9,270
Missing data		4,154 (30.9%)

Variable	Response	N (%) or mean (SD)
**Depression score (Edinburgh post-natal depression score; range = 0-27; higher scores indicate more depressive symptoms)**		4.20 (3.90)
Missing data		4,059 (30.2%)
**Smoking status**	*Never smoked*	4,280 (46.0%)
	Former smoker	1,644 (17.7%)
	Current smoker	3,378 (36.3%)
	Total	9,302
Missing data		4,122 (30.7%)
**Alcohol intake**	*Never*	448 (4.8%)
	Less than 1 glass per week	2,270 (24.3%)
	1 or more glasses per week	4,732 (50.6%)
	1 or more glasses per day	1,908 (20.4%)
	Total	9,358
Missing data		4,066 (30.3%)
**Occupational social class (for more information on these social classes, see: https://sru.soc.surrey.ac.uk/SRU9.html**	*I (Professional)*	1,167 (11.1%)
	II (Managerial and technical)	3,608 (34.2%)
	III Non-manual (Skilled)	1,145 (10.8%)
	III Manual (Skilled)	3,317 (31.4%)
	IV/V (Partly-skilled/unskilled)	1,321 (12.5%)
	Total	10,558
Missing data		2,866 (21.3%)
**Household access to car**	*No*	1,281 (10.2%)
	Yes	11,263 (89.8%)
	Total	12,544
Missing data		880 (6.6%)
**Mother's age at birth (years; range = 17-43)**		28.1 (4.84)
Missing data		4 (0.03%)
**Mother's ethnicity**	*White*	11,547 (97.5%)
	Other than white	302 (2.5%)
	Total	11,849
Missing data		1,575 (11.7%)
**Mother's highest educational qualification (for a comparison of different UK educational qualifications, see: https://www.gov.uk/what-different-qualification-levels-mean/list-of-qualification-levels: for more information about the Certificate of Secondary Education, see: https://en.wikipedia.org/wiki/Certificate_of_Secondary_Education)**	*Certificate of Secondary Education/None*	2,348 (19.7%)
	Vocational qualification	1,169 (9.8%)
	O-level	4,159 (34.8%)
	A-level	2,708 (22.7%)
	Degree or higher	1,561 (13.1%)
	Total	11,945

Variable	Response	N (%) or mean (SD)
Missing data		1,479 (11.0%)
**Mother's employment status**	*Not employed*	7,327 (61.0%)
	Employed	4,682 (39.0%)
	Total	12,009
Missing data		1,415 (10.5%)
**Mother's marital status**	*Currently married*	9,522 (75.6%)
	Never married	2,307 (18.3%)
	Separated/Widowed/Divorced	763 (6.1%)
	Total	12,592
Missing data		832 (6.2%)
**Mother's locus of control (range = 0-12; higher scores indicate a more external locus of control)**		4.35 (2.16)
Missing data		3,271 (24.4%)
**Mother's recent financial difficulties**	*No*	9,930 (86.6%)
	Yes	1,540 (13.4%)
	Total	11,470
Missing data		1,954 (14.6%)
**Mother's self-reported health status**	*Always well*	3,692 (32.0%)
	Usually well	6,960 (60.2%)
	Often/Sometimes/Always unwell	901 (7.8%)
	Total	11,553
Missing data		1,871 (13.9%)
**Mother's belief in God/a divine power**	*No*	5,952 (50.2%)
	Yes	5,912 (49.8%)
	Total	11,864
Missing data		1,560 (11.6%)
**Mother's religious affiliation**	*None*	1,789 (15.3%)
	Yes	9,931 (84.7%)
	Total	11,720
Missing data		1,704 (12.7%)
**Mother's frequency of attendance at a place of worship**	*Occasional attendance/never*	9,967 (85.9%)
	Regular attendance (minimum once a month)	1,632 (14.1%)
	Total	11,599
Missing data		1,825 (13.6%)
**Mother ever donated blood**	*No*	8,313 (70.1%)
	Yes	3,549 (29.9%)
	Total	11,862
Missing data		1,562 (11.6%)

Variable	Response	N (%) or mean (SD)
**Ethnicity (as reported by mother)**	*White*	11,163 (96.1%)
	Other than white	456 (3.9%)
	Total	11,619
Missing data		1,805 (13.4%)
**Highest educational qualification (as reported by mother; for a comparison of different UK educational qualifications, see: https://www.gov.uk/what-different-qualification-levels-mean/list-of-qualification-levels: for more information about the Certificate of Secondary Education, see: https://en.wikipedia.org/wiki/Certificate_of_Secondary_Education)**	*Certificate of Secondary Education/None*	2,955 (25.7%)
	Vocational qualification	966 (8.4%)
	O-level	2,434 (21.2%)
	A-level	3,015 (26.2%)
	Degree or higher	2,117 (18.4%)
	Total	11,487
Missing data		1,937 (14.4%)
**Employment status (as reported by mother)**	*Not employed*	2,007 (16.7%)
	Employed	10,002 (83.3%)
	Total	12,009
Missing data		1,415 (10.5%)

Table S3: Cross-tabulations comparing each of the religiosity exposures and the blood donation outcome with whether the questionnaire containing the religion questions was completed either during or after pregnancy for mothers. Note that, for mothers, both the religion and blood donation questions were asked in the same questionnaire. Both the religious attendance and blood donation associations are robust when including a range of sociodemographic confounders (including age, ethnicity, education, area-level index of multiple deprivation, urban/rural status, home ownership status, employment status, recent financial difficulties and month of questionnaire completion) in a logistic regression model: religious attendance odds ratio = 1.30, 95% confidence interval = 1.04 to 1.63, $p = 0.021$; blood donation odds ratio = 0.83, 95% confidence interval = 0.69 to 1.00, $p = 0.049$.

	**Questionnaire completed during or after pregnancy**	
**Belief in God/a divine power**	*During*	*After*
No	5,135 (88.3%)	682 (11.7%)
Yes	5,114 (88.3%)	680 (11.7%)
		χ^2(1, N = 11,611) = 0.000, p = 0.984
**Religious affiliation**	*During*	*After*
None	1,556 (88.6%)	201 (11.4%)
Yes	8,562 (88.2%)	1,148 (11.8%)
		χ^2(1, N = 11,467) = 0.210, p = 0.647
**Frequency of attendance at a place of worship**	*During*	*After*
Occasional attendance/never	8,643 (88.6%)	1,113 (11.4%)
Regular attendance (minimum once a month)	1,382 (86.3%)	220 (13.7%)
		χ^2(1, N = 11,358) = 7.177, p = 0.007
**Donated blood**	*During*	*After*
No	7,128 (87.9%)	982 (12.1%)
Yes	3,103 (89.2%)	376 (10.8%)
		χ^2(1, N = 11,589) = 3.982, p = 0.046

Table S4: Cross-tabulations comparing each of the religiosity exposures and the blood donation outcome with whether the questionnaire containing the religion questions was completed either during or after pregnancy for partners. Note that, for partners, the religion and blood donation questions were asked in separate questionnaires, so would not expect an association between time of questionnaire completion and blood donation among partners.

	**Questionnaire completed during or after pregnancy**	
**Belief in God/a divine power**	*During*	*After*
No	5,607 (96.9%)	182 (3.1%)
Yes	3,257 (97.0%)	100 (3.0%)
χ^2(1, N = 9,146) = 0.194, p = 0.660		
**Religious affiliation**	*During*	*After*
None	2,251 (97.1%)	68 (2.9%)
Yes	6,473 (96.8%)	212 (3.2%)
χ^2(1, N = 9,004) = 0.326, p = 0.568		
**Frequency of attendance at a place of worship**	*During*	*After*
Occasional attendance/never	7,801 (96.9%)	251 (3.1%)
Regular attendance (minimum once a month)	885 (97.3%)	25 (2.7%)
χ^2(1, N = 8,962) = 0.375, p = 0.540		
**Donated blood**	*During*	*After*
No	4,634 (97.4%)	125 (2.6%)
Yes	2,533 (97.4%)	67 (2.6%)
χ^2(1, N = 7,359) = 0.016, p = 0.898		

Table S5: Cross-tabulations comparing each of the religiosity exposures with the blood donation outcome for mothers.

	**Donated blood**	
**Belief in God/a divine power**	*No*	*Yes*
No	4,282 (72.2%)	1,646 (27.8%)
Yes	3,996 (67.9%)	1,892 (32.1%)
**Religious affiliation**	*No*	*Yes*
None	1,244 (69.8%)	538 (30.2%)
Yes	6,922 (70.0%)	2,969 (30.0%)
**Frequency of attendance at a place of worship**	*No*	*Yes*
Occasional attendance/never	7,049 (71.2%)	2,861 (28.8%)
Regular attendance (minimum once a month)	996 (61.3%)	628 (38.7%)

Table S6: Cross-tabulations comparing each of the religiosity exposures with the blood donation outcome for partners.

	**Donated blood**	
**Belief in God/a divine power**	*No*	*Yes*
No	3,128 (65.9%)	1,618 (34.1%)
Yes	1,721 (62.9%)	1,014 (37.1%)
**Religious affiliation**	*No*	*Yes*
None	1,271 (64.6%)	695 (35.4%)
Yes	3,505 (65.0%)	1,889 (35.0%)
**Frequency of attendance at a place of worship**	*No*	*Yes*
Occasional attendance/never	4,333 (66.1%)	2,226 (33.9%)
Regular attendance (minimum once a month)	406 (52.4%)	369 (47.6%)

Table S7*:* Results of the complete-case and multiple imputation analyses for each religiosity exposure with blood donation as the outcome for mothers. The 'confounders only' scenario adjusts only for assumed confounders, while the 'confounders and/or mediators' scenario adjusts for both assumed confounders and variables which may be both confounders and mediators (see table 2 and figure 3). This table also presents the predicted difference in the probability of donating blood based on each model. CI = Confidence interval.

Model	Exposure	Adjustment set	Odds ratio	Lower 95% CI	Upper 95% CI	*p*-value	Diff. in blood donation (%)	Lower 95% CI of diff.	Upper 95% CI of diff.
Complete-case analysis (*n* = 7,341)	Religious belief	Unadjusted	1.207	1.094	1.332	0.0002	4.05	1.93	6.17
		Confounders only	1.062	0.958	1.178	0.2529	1.20	-0.86	3.26
		Confounders and/or mediators	1.033	0.930	1.147	0.5466	0.64	-1.43	2.70
	Religious affiliation	Unadjusted	0.988	0.859	1.137	0.8625	-0.27	-3.29	2.76
		Confounders only	1.023	0.882	1.189	0.7607	0.46	-2.49	3.41
		Confounders and/or mediators	0.998	0.858	1.162	0.9748	-0.05	-3.04	2.94
	Religious attendance	Unadjusted	1.506	1.311	1.729	<0.0001	9.26	6.00	12.51
		Confounders only	1.154	0.996	1.336	0.0563	2.90	-0.13	5.92
		Confounders and/or mediators	1.148	0.988	1.332	0.0711	2.76	-0.28	5.80
Multiple imputation analysis (*n* = 13,477)	Religious belief	Unadjusted	1.247	1.151	1.350	0.0000	4.52	2.89	6.15
		Confounders only	1.062	0.976	1.157	0.1643	1.12	-0.46	2.69
		Confounders and/or mediators	1.029	0.943	1.122	0.5217	0.52	-1.07	2.10
	Religious affiliation	Unadjusted	1.013	0.908	1.131	0.8142	0.27	-1.97	2.51
		Confounders only	1.028	0.914	1.157	0.6446	0.51	-1.66	2.68
		Confounders and/or mediators	0.993	0.881	1.120	0.9132	-0.12	-2.32	2.07
	Religious attendance	Unadjusted	1.598	1.432	1.784	<0.0001	10.23	7.71	12.76
		Confounders only	1.156	1.027	1.301	0.0163	2.73	0.46	5.00
		Confounders and/or mediators	1.135	1.007	1.280	0.0387	2.35	0.09	4.62

Table S8: Results of the complete-case and multiple imputation analyses for each religiosity exposure with blood donation as the outcome for partners. The 'confounders only' scenario adjusts only for assumed confounders, while the 'confounders and/or mediators' scenario adjusts for both assumed confounders and variables which may be both confounders and mediators (see table 2 and figure 3). This table also presents the predicted difference in the probability of donating blood based on each model. CI = Confidence interval.

**Model**	**Exposure**	**Adjustment set**	**Odds ratio**	**Lower 95% CI**	**Upper 95% CI**	***p*-value**	**Diff. in blood donation (%)**	**Lower 95% CI of diff.**	**Upper 95% CI of diff.**
Complete-case analysis (*n* = 5,305)	Religious belief	Unadjusted	1.159	1.032	1.302	0.0126	3.41	0.72	6.09
		Confounders only	1.091	0.967	1.230	0.1562	1.89	-0.73	4.51
		Confounders and/or mediators	1.067	0.946	1.204	0.2901	1.41	-1.21	4.03
	Religious affiliation	Unadjusted	1.015	0.893	1.154	0.8227	0.34	-2.60	3.27
		Confounders only	1.059	0.927	1.210	0.4000	1.23	-1.63	4.09
		Confounders and/or mediators	1.043	0.912	1.192	0.5400	0.90	-1.97	3.77
	Religious attendance	Unadjusted	1.891	1.577	2.267	<0.0001	15.36	10.88	19.84
		Confounders only	1.498	1.240	1.809	<0.0001	9.09	4.73	13.46
		Confounders and/or mediators	1.437	1.188	1.737	0.0002	8.08	3.72	12.44
Multiple imputation analysis (*n* = 13,424)	Religious belief	Unadjusted	1.119	1.007	1.242	0.0367	2.44	0.16	4.72
		Confounders only	1.060	0.952	1.180	0.2859	1.18	-0.98	3.34
		Confounders and/or mediators	1.034	0.927	1.152	0.5499	0.66	-1.50	2.83
	Religious affiliation	Unadjusted	0.987	0.886	1.099	0.8114	-0.28	-2.61	2.04
		Confounders only	1.039	0.928	1.163	0.5083	0.76	-1.49	3.02
		Confounders and/or mediators	1.010	0.902	1.131	0.8609	0.20	-2.06	2.46
	Religious attendance	Unadjusted	1.781	1.535	2.067	<0.0001	13.37	9.78	16.97
		Confounders only	1.367	1.172	1.594	0.0001	6.55	3.23	9.87
		Confounders and/or mediators	1.292	1.105	1.510	0.0014	5.29	1.98	8.60

Table S9: Results of the Not-At-Random Multiple Imputation analyses for mothers, assuming that just the exposure 'religious attendance' is Missing-Not-At-Random (that is, individuals with missing religious attendance data were less likely to attend a place of worship regularly; *n* = 13,477). This Not-At-Random Multiple Imputation sensitivity analysis compares the results of different 'Conditional Sensitivity Parameters', which are the extent to which those with missing data differ to those with observed data, conditional on all other variables in the imputation model. This is on the log-odds scale, which ranged from -2 to 0 in steps of 0.25. To aid interpretation, these conditional sensitivity parameters have been converted to 'marginal sensitivity parameters' (that is, the marginal difference between those with vs without data, on the log-odds scale), 'ignorable missingness odds ratios' (that is, the marginal difference between those with vs without missing data, on the odds ratio scale) and prevalence estimates in the sample. For each conditional sensitivity parameter, we generated 50 imputations with a burn-in period of 10 iterations. Other than the addition of the sensitivity parameter, the imputation model was identical to that of the standard multiple imputation analysis. Results are repeated for the unadjusted model, the model adjusting for potential confounders only, and the model adjusting for potential confounders and/or mediators. In this analysis, there is little variation in the effect estimates, suggesting that results are unlikely to be biased by selection if the exposure is Missing-Not-At-Random. For a clearer presentation of these results for the confounders only adjustment set, see figure S7. CSP = Conditional Sensitivity Parameter; MSP = Marginal Sensitivity Parameter; IMOR = Ignorable Missingness Odds Ratio; Prev. = Prevalence; OR = Odds Ratio; CI = Confidence Interval.

CSP	*MSP*	*IMOR*	*Prev.*	**Unadjusted**				**Confounders only**				**Confounders and/or mediators**			
				OR	*Lower 95% CI*	*Upper 95% CI*	*p-value*	*OR*	*Lower 95% CI*	*Upper 95% CI*	*p-value*	*OR*	*Lower 95% CI*	*Upper 95% CI*	*p-value*
-2	-2.252	0.105	0.124	1.657	1.486	1.846	<0.001	1.163	1.034	1.307	0.012	1.141	1.013	1.285	0.029
-1.75	-2.044	0.129	0.124	1.652	1.483	1.842	<0.001	1.163	1.036	1.306	0.011	1.142	1.015	1.284	0.027
-1.5	-1.791	0.167	0.125	1.649	1.480	1.839	<0.001	1.163	1.035	1.306	0.011	1.142	1.014	1.287	0.028
-1.25	-1.554	0.211	0.126	1.645	1.477	1.833	<0.001	1.163	1.036	1.306	0.011	1.143	1.016	1.287	0.026
-1	-1.312	0.269	0.127	1.642	1.474	1.829	<0.001	1.164	1.037	1.306	0.010	1.142	1.016	1.284	0.026
-0.75	-1.124	0.325	0.128	1.629	1.461	1.817	<0.001	1.157	1.029	1.301	0.014	1.137	1.009	1.280	0.035
-0.5	-0.845	0.430	0.130	1.624	1.455	1.811	<0.001	1.163	1.035	1.307	0.012	1.142	1.014	1.288	0.028
-0.25	-0.637	0.529	0.132	1.610	1.445	1.793	<0.001	1.156	1.028	1.298	0.015	1.135	1.010	1.278	0.034
0	-0.401	0.670	0.135	1.598	1.432	1.786	<0.001	1.157	1.027	1.305	0.016	1.138	1.008	1.284	0.037

Table S10: Results of the Not-At-Random Multiple Imputation analyses for mothers, assuming that just the outcome ‘blood donation’ is Missing-Not-At-Random (that is, individuals with missing blood donation data were less likely to donate blood; *n* = 13,477). This Not-At-Random Multiple Imputation sensitivity analysis compares the results of different ‘Conditional Sensitivity Parameters’, which are the extent to which those with missing data differ to those with observed data, conditional on all other variables in the imputation model. This is on the log-odds scale, which ranged from -2 to 0 in steps of 0.25. To aid interpretation, these conditional sensitivity parameters have been converted to ‘marginal sensitivity parameters’ (that is, the marginal difference between those with vs without data, on the log-odds scale), ‘ignorable missingness odds ratios’ (that is, the marginal difference between those with vs without missing data, on the odds ratio scale) and prevalence estimates in the sample. For each conditional sensitivity parameter, we generated 50 imputations with a burn-in period of 10 iterations. Other than the addition of the sensitivity parameter, the imputation model was identical to that of the standard multiple imputation analysis. Results are repeated for the unadjusted model, the model adjusting for potential confounders only, and the model adjusting for potential confounders and/or mediators. In this analysis, there is little variation in the effect estimates, suggesting that results are unlikely to be biased by selection if the outcome is Missing-Not-At-Random. For a clearer presentation of these results for the confounders only adjustment set, see figure S8. CSP = Conditional Sensitivity Parameter; MSP = Marginal Sensitivity Parameter; IMOR = Ignorable Missingness Odds Ratio; Prev. = Prevalence; OR = Odds Ratio; CI = Confidence Interval.

CSP	*MSP*	*IMOR*	*Prev.*	**Unadjusted**				**Confounders only**				**Confounders and/or mediators**			
				OR	*Lower 95% CI*	*Upper 95% CI*	*p-value*	*OR*	*Lower 95% CI*	*Upper 95% CI*	*p-value*	*OR*	*Lower 95% CI*	*Upper 95% CI*	*p-value*
-2	-2.362	0.094	0.268	1.616	1.452	1.799	<0.001	1.150	1.024	1.292	0.018	1.127	1.002	1.269	0.046
-1.75	-2.124	0.120	0.269	1.614	1.451	1.799	<0.001	1.151	1.025	1.293	0.017	1.130	1.004	1.270	0.043
-1.5	-1.869	0.154	0.271	1.618	1.452	1.804	<0.001	1.156	1.028	1.300	0.015	1.131	1.004	1.275	0.042
-1.25	-1.616	0.199	0.273	1.611	1.445	1.795	<0.001	1.150	1.024	1.293	0.018	1.127	1.002	1.270	0.046
-1	-1.383	0.251	0.275	1.611	1.446	1.795	<0.001	1.150	1.025	1.292	0.017	1.127	1.003	1.269	0.045
-0.75	-1.144	0.319	0.278	1.613	1.446	1.799	<0.001	1.155	1.029	1.296	0.014	1.132	1.008	1.273	0.037
-0.5	-0.905	0.405	0.281	1.605	1.439	1.790	<0.001	1.154	1.027	1.297	0.016	1.132	1.006	1.275	0.040
-0.25	-0.680	0.507	0.284	1.594	1.428	1.779	<0.001	1.148	1.021	1.290	0.021	1.127	1.002	1.270	0.047
0	-0.461	0.630	0.288	1.598	1.435	1.781	<0.001	1.155	1.029	1.296	0.014	1.135	1.010	1.276	0.034

Table S11: Results of the Not-At-Random Multiple Imputation analyses for mothers, assuming that both the exposure 'religious attendance' and the outcome 'blood donation' are Missing-Not-At-Random (that is, individuals with missing religious attendance data were less likely to attend a place of worship regularly *and* individuals with missing blood donation data were less likely to donate blood; *n* = 13,477). This Not-At-Random Multiple Imputation sensitivity analysis compares the results of different 'Conditional Sensitivity Parameters', which are the extent to which those with missing data differ to those with observed data, conditional on all other variables in the imputation model. This is on the log-odds scale, which ranged from -2 to 0 in steps of 0.25, looping over both the exposure and outcome. To aid interpretation, these conditional sensitivity parameters have been converted to 'marginal sensitivity parameters' (that is, the marginal difference between those with vs without data, on the log-odds scale), 'ignorable missingness odds ratios' (that is, the marginal difference between those with vs without missing data, on the odds ratio scale) and prevalence estimates in the sample. For each conditional sensitivity parameter, we generated 50 imputations with a burn-in period of 10 iterations. Other than the addition of the sensitivity parameters, the imputation model was identical to that of the standard multiple imputation analysis. Results are repeated for the unadjusted model, the model adjusting for potential confounders only, and the model adjusting for potential confounders and/or mediators. In this analysis, if both the exposure and outcome are Missing-Not-At-Random, then the true effect estimate is larger than the observed effect estimate in the complete-case and standard multiple imputation analyses; this is because, if both the exposure and outcome are negatively associated with selection, then this will bias the effect estimate downwards, so accounting for this results in a larger effect estimate here. These differences are relatively minor however, likely because the amount of missing data in these variables is quite small (approx. 12% in both); for a clearer presentation of these results for the confounders only adjustment set, see figure S9. CSP = Conditional Sensitivity Parameter; MSP = Marginal Sensitivity Parameter; IMOR = Ignorable Missingness Odds Ratio; Prev. = Prevalence; OR = Odds Ratio; CI = Confidence Interval; Out. = Outcome (blood donation); Exp. = Exposure (religious attendance).

CSP out.	*MSP out.*	*IMOR out.*	*Prev. out.*	*CSP exp.*	*MSP exp.*	*IMOR exp.*	*Prev. exp.*	**Unadjusted**				**Confounders only**				**Confounders and/or mediators**			
								OR	*Lower 95% CI*	*Upper 95% CI*	*p-value*	*OR*	*Lower 95% CI*	*Upper 95% CI*	*p-value*	*OR*	*Lower 95% CI*	*Upper 95% CI*	*p-value*
-2	-2.358	0.095	0.268	-2	-2.330	0.097	0.123	1.811	1.624	2.018	<0.001	1.265	1.126	1.422	<0.001	1.239	1.101	1.395	<0.001
-1.75	-2.102	0.122	0.269	-2	-2.328	0.097	0.123	1.802	1.616	2.008	<0.001	1.260	1.122	1.416	<0.001	1.235	1.096	1.391	0.001
-1.5	-1.874	0.154	0.271	-2	-2.388	0.092	0.123	1.791	1.608	1.998	<0.001	1.252	1.114	1.406	<0.001	1.226	1.089	1.380	0.001
-1.25	-1.624	0.197	0.273	-2	-2.360	0.094	0.123	1.777	1.594	1.980	<0.001	1.242	1.105	1.395	<0.001	1.218	1.081	1.372	0.001
-1	-1.402	0.246	0.275	-2	-2.343	0.096	0.123	1.763	1.581	1.964	<0.001	1.230	1.094	1.383	0.001	1.206	1.070	1.358	0.002
-0.75	-1.162	0.313	0.277	-2	-2.319	0.098	0.123	1.738	1.559	1.937	<0.001	1.217	1.083	1.368	0.001	1.194	1.061	1.344	0.003
-0.5	-0.933	0.393	0.280	-2	-2.273	0.103	0.124	1.711	1.534	1.906	<0.001	1.196	1.065	1.343	0.003	1.174	1.043	1.320	0.008
-0.25	-0.700	0.497	0.284	-2	-2.290	0.101	0.124	1.685	1.510	1.881	<0.001	1.183	1.052	1.330	0.005	1.162	1.031	1.309	0.014
0	-0.458	0.632	0.288	-2	-2.299	0.100	0.123	1.652	1.483	1.840	<0.001	1.160	1.033	1.302	0.012	1.140	1.013	1.283	0.030
-2	-2.347	0.096	0.268	-1.75	-2.044	0.130	0.124	1.799	1.614	2.006	<0.001	1.260	1.121	1.415	<0.001	1.234	1.095	1.388	0.001
-1.75	-2.106	0.122	0.269	-1.75	-2.048	0.129	0.124	1.788	1.603	1.994	<0.001	1.250	1.112	1.405	<0.001	1.224	1.087	1.379	0.001
-1.5	-1.863	0.155	0.271	-1.75	-2.118	0.120	0.124	1.782	1.600	1.988	<0.001	1.246	1.110	1.401	<0.001	1.223	1.085	1.376	0.001
-1.25	-1.646	0.193	0.273	-1.75	-2.059	0.128	0.124	1.766	1.584	1.970	<0.001	1.235	1.099	1.390	<0.001	1.210	1.075	1.365	0.002
-1	-1.395	0.248	0.275	-1.75	-2.093	0.123	0.124	1.751	1.571	1.950	<0.001	1.226	1.092	1.379	0.001	1.202	1.067	1.353	0.002

-0.75	-1.151	0.316	0.278	-1.75	-2.050	0.129	0.124	1.728	1.550	1.925	<0.001	1.209	1.076	1.359	0.001	1.185	1.052	1.335	0.005
-0.5	-0.918	0.399	0.281	-1.75	-2.037	0.130	0.124	1.707	1.531	1.904	<0.001	1.197	1.065	1.347	0.003	1.175	1.042	1.323	0.008
-0.25	-0.703	0.495	0.284	-1.75	-2.086	0.124	0.124	1.679	1.505	1.872	<0.001	1.179	1.049	1.326	0.006	1.157	1.028	1.302	0.015
0	-0.481	0.618	0.288	-1.75	-2.025	0.132	0.124	1.654	1.484	1.844	<0.001	1.163	1.036	1.306	0.011	1.143	1.015	1.288	0.027
-2	-2.362	0.094	0.268	-1.5	-1.882	0.152	0.125	1.788	1.605	1.994	<0.001	1.255	1.116	1.409	<0.001	1.230	1.092	1.385	0.001
-1.75	-2.113	0.121	0.269	-1.5	-1.893	0.151	0.125	1.782	1.600	1.986	<0.001	1.247	1.111	1.402	<0.001	1.223	1.085	1.376	0.001
-1.5	-1.856	0.156	0.271	-1.5	-1.830	0.160	0.125	1.766	1.586	1.970	<0.001	1.240	1.104	1.392	<0.001	1.214	1.078	1.366	0.001
-1.25	-1.616	0.199	0.273	-1.5	-1.841	0.159	0.125	1.754	1.573	1.954	<0.001	1.228	1.092	1.380	0.001	1.201	1.066	1.353	0.003
-1	-1.399	0.247	0.275	-1.5	-1.857	0.156	0.125	1.740	1.560	1.939	<0.001	1.220	1.085	1.372	0.001	1.196	1.063	1.347	0.003
-0.75	-1.159	0.314	0.277	-1.5	-1.837	0.159	0.125	1.721	1.543	1.917	<0.001	1.208	1.075	1.357	0.001	1.184	1.052	1.334	0.005
-0.5	-0.943	0.389	0.280	-1.5	-1.776	0.169	0.125	1.702	1.527	1.898	<0.001	1.196	1.064	1.344	0.003	1.174	1.042	1.322	0.008
-0.25	-0.701	0.496	0.284	-1.5	-1.784	0.168	0.125	1.677	1.504	1.870	<0.001	1.179	1.049	1.326	0.006	1.157	1.028	1.303	0.016
0	-0.493	0.611	0.288	-1.5	-1.824	0.161	0.125	1.652	1.483	1.842	<0.001	1.165	1.037	1.309	0.010	1.142	1.015	1.287	0.027
-2	-2.376	0.093	0.268	-1.25	-1.635	0.195	0.125	1.772	1.589	1.976	<0.001	1.245	1.107	1.399	<0.001	1.220	1.083	1.374	0.001
-1.75	-2.132	0.119	0.269	-1.25	-1.585	0.205	0.126	1.763	1.581	1.964	<0.001	1.237	1.102	1.390	<0.001	1.212	1.077	1.363	0.001
-1.5	-1.863	0.155	0.271	-1.25	-1.597	0.202	0.126	1.749	1.570	1.950	<0.001	1.229	1.093	1.380	0.001	1.203	1.069	1.355	0.002
-1.25	-1.636	0.195	0.273	-1.25	-1.575	0.207	0.126	1.738	1.560	1.939	<0.001	1.221	1.087	1.372	0.001	1.196	1.062	1.346	0.003
-1	-1.406	0.245	0.275	-1.25	-1.616	0.199	0.126	1.726	1.550	1.923	<0.001	1.212	1.079	1.361	0.001	1.188	1.057	1.336	0.004
-0.75	-1.145	0.318	0.278	-1.25	-1.563	0.210	0.126	1.707	1.533	1.904	<0.001	1.201	1.068	1.350	0.002	1.178	1.047	1.326	0.007
-0.5	-0.932	0.394	0.280	-1.25	-1.549	0.212	0.126	1.687	1.513	1.881	<0.001	1.186	1.057	1.334	0.004	1.164	1.034	1.311	0.012
-0.25	-0.681	0.506	0.284	-1.25	-1.557	0.211	0.126	1.664	1.490	1.857	<0.001	1.172	1.043	1.318	0.008	1.153	1.022	1.298	0.020
0	-0.463	0.629	0.288	-1.25	-1.530	0.216	0.126	1.644	1.476	1.831	<0.001	1.164	1.036	1.309	0.011	1.143	1.015	1.287	0.027
-2	-2.369	0.094	0.268	-1	-1.391	0.249	0.127	1.752	1.573	1.954	<0.001	1.232	1.096	1.384	<0.001	1.207	1.073	1.358	0.002
-1.75	-2.108	0.121	0.269	-1	-1.335	0.263	0.127	1.740	1.562	1.941	<0.001	1.224	1.089	1.374	0.001	1.198	1.065	1.350	0.003
-1.5	-1.877	0.153	0.271	-1	-1.369	0.254	0.127	1.735	1.557	1.933	<0.001	1.221	1.088	1.372	0.001	1.197	1.064	1.346	0.003
-1.25	-1.601	0.202	0.273	-1	-1.355	0.258	0.127	1.719	1.543	1.917	<0.001	1.210	1.078	1.359	0.001	1.186	1.053	1.335	0.005
-1	-1.393	0.248	0.275	-1	-1.338	0.262	0.127	1.711	1.536	1.904	<0.001	1.204	1.073	1.353	0.002	1.181	1.049	1.328	0.006
-0.75	-1.157	0.314	0.277	-1	-1.333	0.264	0.127	1.696	1.522	1.889	<0.001	1.195	1.064	1.343	0.003	1.171	1.041	1.318	0.009
-0.5	-0.934	0.393	0.280	-1	-1.353	0.258	0.127	1.682	1.507	1.878	<0.001	1.188	1.057	1.336	0.004	1.165	1.035	1.314	0.012
-0.25	-0.692	0.500	0.284	-1	-1.346	0.260	0.127	1.657	1.486	1.848	<0.001	1.172	1.043	1.317	0.008	1.149	1.020	1.293	0.022
0	-0.467	0.627	0.288	-1	-1.307	0.271	0.127	1.636	1.468	1.824	<0.001	1.160	1.031	1.302	0.013	1.140	1.013	1.283	0.030
-2	-2.341	0.096	0.268	-0.75	-1.127	0.324	0.128	1.721	1.545	1.917	<0.001	1.214	1.081	1.363	0.001	1.189	1.057	1.339	0.004
-1.75	-2.076	0.125	0.270	-0.75	-1.138	0.320	0.128	1.716	1.537	1.914	<0.001	1.210	1.077	1.361	0.001	1.186	1.052	1.336	0.005
-1.5	-1.869	0.154	0.271	-0.75	-1.158	0.314	0.128	1.711	1.534	1.908	<0.001	1.204	1.073	1.354	0.002	1.179	1.047	1.327	0.006
-1.25	-1.639	0.194	0.273	-0.75	-1.149	0.317	0.128	1.706	1.528	1.902	<0.001	1.203	1.069	1.353	0.002	1.177	1.045	1.326	0.007
-1	-1.395	0.248	0.275	-0.75	-1.136	0.321	0.128	1.699	1.525	1.895	<0.001	1.200	1.067	1.349	0.002	1.176	1.045	1.323	0.007
-0.75	-1.147	0.318	0.278	-0.75	-1.127	0.324	0.128	1.680	1.507	1.872	<0.001	1.188	1.057	1.335	0.004	1.166	1.036	1.313	0.011
-0.5	-0.925	0.397	0.281	-0.75	-1.118	0.327	0.128	1.660	1.490	1.852	<0.001	1.176	1.047	1.320	0.006	1.154	1.024	1.298	0.018
-0.25	-0.691	0.501	0.284	-0.75	-1.100	0.333	0.128	1.645	1.477	1.835	<0.001	1.168	1.040	1.311	0.009	1.147	1.019	1.290	0.023

0	-0.477	0.620	0.288	-0.75	-1.086	0.338	0.128	1.626	1.458	1.813	<0.001	1.156	1.028	1.301	0.016	1.134	1.007	1.278	0.039
-2	-2.411	0.090	0.268	-0.5	-0.891	0.410	0.130	1.692	1.517	1.885	<0.001	1.197	1.064	1.346	0.003	1.171	1.040	1.320	0.009
-1.75	-2.098	0.123	0.270	-0.5	-0.913	0.401	0.130	1.690	1.516	1.883	<0.001	1.197	1.066	1.346	0.002	1.174	1.042	1.320	0.008
-1.5	-1.835	0.160	0.271	-0.5	-0.902	0.406	0.130	1.680	1.508	1.874	<0.001	1.190	1.060	1.336	0.003	1.166	1.037	1.313	0.011
-1.25	-1.629	0.196	0.273	-0.5	-0.892	0.410	0.130	1.682	1.510	1.874	<0.001	1.192	1.061	1.339	0.003	1.168	1.038	1.314	0.010
-1	-1.401	0.246	0.275	-0.5	-0.874	0.417	0.130	1.670	1.499	1.861	<0.001	1.184	1.054	1.330	0.004	1.162	1.033	1.307	0.013
-0.75	-1.150	0.317	0.278	-0.5	-0.881	0.415	0.130	1.662	1.489	1.853	<0.001	1.177	1.046	1.324	0.007	1.155	1.024	1.302	0.019
-0.5	-0.927	0.396	0.280	-0.5	-0.868	0.420	0.130	1.647	1.476	1.839	<0.001	1.171	1.042	1.317	0.008	1.149	1.020	1.294	0.022
-0.25	-0.674	0.510	0.284	-0.5	-0.872	0.418	0.130	1.637	1.468	1.826	<0.001	1.163	1.036	1.307	0.011	1.142	1.014	1.287	0.028
0	-0.469	0.626	0.288	-0.5	-0.844	0.430	0.130	1.614	1.449	1.800	<0.001	1.154	1.026	1.297	0.016	1.133	1.006	1.276	0.039
-2	-2.341	0.096	0.268	-0.25	-0.670	0.512	0.132	1.655	1.486	1.842	<0.001	1.175	1.047	1.318	0.006	1.150	1.022	1.293	0.020
-1.75	-2.092	0.123	0.270	-0.25	-0.686	0.503	0.132	1.655	1.486	1.846	<0.001	1.174	1.045	1.319	0.007	1.150	1.022	1.296	0.020
-1.5	-1.880	0.153	0.271	-0.25	-0.666	0.514	0.132	1.654	1.484	1.842	<0.001	1.175	1.045	1.320	0.007	1.151	1.022	1.297	0.020
-1.25	-1.609	0.200	0.273	-0.25	-0.667	0.513	0.132	1.647	1.478	1.835	<0.001	1.170	1.041	1.315	0.009	1.148	1.019	1.293	0.023
-1	-1.374	0.253	0.275	-0.25	-0.657	0.518	0.132	1.644	1.477	1.829	<0.001	1.171	1.043	1.315	0.008	1.148	1.019	1.292	0.022
-0.75	-1.137	0.321	0.278	-0.25	-0.636	0.530	0.132	1.632	1.465	1.820	<0.001	1.163	1.035	1.307	0.011	1.139	1.012	1.281	0.031
-0.5	-0.924	0.397	0.281	-0.25	-0.639	0.528	0.132	1.627	1.462	1.809	<0.001	1.160	1.034	1.301	0.012	1.139	1.013	1.280	0.030
-0.25	-0.685	0.504	0.284	-0.25	-0.645	0.525	0.132	1.619	1.454	1.804	<0.001	1.158	1.030	1.301	0.013	1.138	1.010	1.280	0.033
0	-0.459	0.632	0.288	-0.25	-0.640	0.527	0.132	1.606	1.443	1.788	<0.001	1.154	1.028	1.294	0.015	1.133	1.008	1.275	0.037
-2	-2.377	0.093	0.268	0	-0.437	0.646	0.134	1.616	1.449	1.802	<0.001	1.154	1.026	1.298	0.017	1.131	1.003	1.274	0.044
-1.75	-2.105	0.122	0.269	0	-0.443	0.642	0.134	1.616	1.448	1.804	<0.001	1.154	1.026	1.297	0.016	1.131	1.004	1.273	0.042
-1.5	-1.863	0.155	0.271	0	-0.445	0.641	0.134	1.613	1.446	1.799	<0.001	1.153	1.026	1.296	0.017	1.130	1.003	1.273	0.044
-1.25	-1.614	0.199	0.273	0	-0.445	0.641	0.134	1.613	1.449	1.797	<0.001	1.154	1.027	1.296	0.015	1.131	1.005	1.273	0.041
-1	-1.389	0.249	0.275	0	-0.439	0.645	0.134	1.616	1.451	1.800	<0.001	1.156	1.029	1.298	0.014	1.134	1.008	1.276	0.037
-0.75	-1.133	0.322	0.278	0	-0.418	0.658	0.134	1.614	1.449	1.799	<0.001	1.157	1.030	1.300	0.014	1.135	1.009	1.276	0.035
-0.5	-0.929	0.395	0.280	0	-0.439	0.644	0.134	1.608	1.443	1.791	<0.001	1.155	1.028	1.297	0.015	1.132	1.005	1.275	0.041
-0.25	-0.685	0.504	0.284	0	-0.435	0.647	0.134	1.605	1.441	1.790	<0.001	1.157	1.029	1.300	0.014	1.135	1.008	1.279	0.036
0	-0.455	0.634	0.288	0	-0.401	0.669	0.135	1.600	1.438	1.781	<0.001	1.156	1.031	1.296	0.013	1.135	1.011	1.275	0.032

Table S12: Results of the Not-At-Random Multiple Imputation analyses for partners, assuming that just the exposure ‘religious attendance’ is Missing-Not-At-Random (that is, individuals with missing religious attendance data were less likely to attend a place of worship regularly; *n* = 13,424). This Not-At-Random Multiple Imputation sensitivity analysis compares the results of different ‘Conditional Sensitivity Parameters’, which are the extent to which those with missing data differ to those with observed data, conditional on all other variables in the imputation model. This is on the log-odds scale, which ranged from -2 to 0 in steps of 0.25. To aid interpretation, these conditional sensitivity parameters have been converted to ‘marginal sensitivity parameters’ (that is, the marginal difference between those with vs without data, on the log-odds scale), ‘ignorable missingness odds ratios’ (that is, the marginal difference between those with vs without missing data, on the odds ratio scale) and prevalence estimates in the sample. For each conditional sensitivity parameter, we generated 50 imputations with a burn-in period of 10 iterations. Other than the addition of the sensitivity parameter, the imputation model was identical to that of the standard multiple imputation analysis. Results are repeated for the unadjusted model, the model adjusting for potential confounders only, and the model adjusting for potential confounders and/or mediators. In this analysis, there is little variation in the effect estimates, suggesting that results are unlikely to be biased by selection if the exposure is Missing-Not-At-Random. For a clearer presentation of these results for the confounders only adjustment set, see figure S10. CSP = Conditional Sensitivity Parameter; MSP = Marginal Sensitivity Parameter; IMOR = Ignorable Missingness Odds Ratio; Prev. = Prevalence; OR = Odds Ratio; CI = Confidence Interval.

				**Unadjusted**				**Confounders only**				**Confounders and/or mediators**			
CSP	*MSP*	*IMOR*	*Prev.*	*OR*	*Lower 95% CI*	*Upper 95% CI*	*p-value*	*OR*	*Lower 95% CI*	*Upper 95% CI*	*p-value*	*OR*	*Lower 95% CI*	*Upper 95% CI*	*p-value*
-2	-1.748	0.174	0.076	1.935	1.665	2.250	<0.001	1.412	1.207	1.652	<0.001	1.327	1.133	1.554	<0.001
-1.75	-1.547	0.213	0.077	1.921	1.664	2.219	<0.001	1.402	1.202	1.637	<0.001	1.320	1.127	1.545	0.001
-1.5	-1.349	0.259	0.079	1.917	1.660	2.217	<0.001	1.408	1.206	1.644	<0.001	1.323	1.131	1.548	0.001
-1.25	-1.136	0.321	0.081	1.893	1.631	2.197	<0.001	1.390	1.183	1.634	<0.001	1.307	1.111	1.539	0.001
-1	-0.948	0.388	0.083	1.865	1.614	2.153	<0.001	1.385	1.191	1.610	<0.001	1.303	1.120	1.519	0.001
-0.75	-0.764	0.466	0.085	1.853	1.598	2.149	<0.001	1.384	1.181	1.621	<0.001	1.300	1.107	1.525	0.001
-0.5	-0.590	0.554	0.088	1.817	1.567	2.106	<0.001	1.361	1.164	1.592	<0.001	1.284	1.099	1.502	0.002
-0.25	-0.407	0.666	0.092	1.782	1.530	2.077	<0.001	1.351	1.147	1.592	<0.001	1.274	1.081	1.501	0.004
0	-0.248	0.781	0.095	1.779	1.540	2.054	<0.001	1.359	1.166	1.584	<0.001	1.283	1.099	1.496	0.002

Table S13: Results of the Not-At-Random Multiple Imputation analyses for partners, assuming that just the outcome 'blood donation' is Missing-Not-At-Random (that is, individuals with missing blood donation data were less likely to donate blood; *n* = 13,424). This Not-At-Random Multiple Imputation sensitivity analysis compares the results of different 'Conditional Sensitivity Parameters', which are the extent to which those with missing data differ to those with observed data, conditional on all other variables in the imputation model. This is on the log-odds scale, which ranged from -2 to 0 in steps of 0.25. To aid interpretation, these conditional sensitivity parameters have been converted to 'marginal sensitivity parameters' (that is, the marginal difference between those with vs without data, on the log-odds scale), 'ignorable missingness odds ratios' (that is, the marginal difference between those with vs without missing data, on the odds ratio scale) and prevalence estimates in the sample. For each conditional sensitivity parameter, we generated 50 imputations with a burn-in period of 10 iterations. Other than the addition of the sensitivity parameter, the imputation model was identical to that of the standard multiple imputation analysis. Results are repeated for the unadjusted model, the model adjusting for potential confounders only, and the model adjusting for potential confounders and/or mediators. In this analysis, there is little variation in the effect estimates, suggesting that results are unlikely to be biased by selection if the outcome is Missing-Not-At-Random. For a clearer presentation of these results for the confounders only adjustment set, see figure S11. CSP = Conditional Sensitivity Parameter; MSP = Marginal Sensitivity Parameter; IMOR = Ignorable Missingness Odds Ratio; Prev. = Prevalence; OR = Odds Ratio; CI = Confidence Interval.

CSP	*MSP*	*IMOR*	*Prev.*	**Unadjusted**				**Confounders only**				**Confounders and/or mediators**			
				OR	*Lower 95% CI*	*Upper 95% CI*	*p-value*	*OR*	*Lower 95% CI*	*Upper 95% CI*	*p-value*	*OR*	*Lower 95% CI*	*Upper 95% CI*	*p-value*
-2	-2.159	0.115	0.230	1.795	1.557	2.067	<0.001	1.357	1.168	1.576	<0.001	1.267	1.090	1.476	0.002
-1.75	-1.911	0.148	0.236	1.797	1.556	2.073	<0.001	1.354	1.164	1.575	<0.001	1.266	1.084	1.477	0.003
-1.5	-1.684	0.186	0.242	1.815	1.586	2.075	<0.001	1.376	1.194	1.587	<0.001	1.289	1.116	1.487	0.001
-1.25	-1.437	0.238	0.251	1.813	1.570	2.094	<0.001	1.368	1.172	1.594	<0.001	1.279	1.094	1.495	0.002
-1	-1.213	0.297	0.260	1.808	1.571	2.077	<0.001	1.372	1.183	1.590	<0.001	1.285	1.104	1.495	0.001
-0.75	-0.973	0.378	0.272	1.817	1.578	2.094	<0.001	1.373	1.181	1.597	<0.001	1.289	1.106	1.502	0.001
-0.5	-0.739	0.478	0.286	1.818	1.567	2.111	<0.001	1.379	1.184	1.605	<0.001	1.293	1.108	1.507	0.001
-0.25	-0.511	0.600	0.301	1.781	1.554	2.038	<0.001	1.350	1.166	1.564	<0.001	1.271	1.094	1.477	0.002
0	-0.293	0.746	0.318	1.768	1.525	2.052	<0.001	1.355	1.156	1.590	<0.001	1.278	1.089	1.499	0.003

Table S14: Results of the Not-At-Random Multiple Imputation analyses for mothers, assuming that both the exposure 'religious attendance' and the outcome 'blood donation' are Missing-Not-At-Random (that is, individuals with missing religious attendance data were less likely to attend a place of worship regularly *and* individuals with missing blood donation data were less likely to donate blood; *n* = 13,424). This Not-At-Random Multiple Imputation sensitivity analysis compares the results of different 'Conditional Sensitivity Parameters', which are the extent to which those with missing data differ to those with observed data, conditional on all other variables in the imputation model. This is on the log-odds scale, which ranged from -2 to 0 in steps of 0.25, looping over both the exposure and outcome. To aid interpretation, these conditional sensitivity parameters have been converted to 'marginal sensitivity parameters' (that is, the marginal difference between those with vs without data, on the log-odds scale), 'ignorable missingness odds ratios' (that is, the marginal difference between those with vs without missing data, on the odds ratio scale) and prevalence estimates in the sample. For each conditional sensitivity parameter, we generated 50 imputations with a burn-in period of 10 iterations. Other than the addition of the sensitivity parameters, the imputation model was identical to that of the standard multiple imputation analysis. Results are repeated for the unadjusted model, the model adjusting for potential confounders only, and the model adjusting for potential confounders and/or mediators. In this analysis, if both the exposure and outcome are Missing-Not-At-Random, then the true effect estimate is larger than the observed effect estimate in the complete-case and standard multiple imputation analyses; this is because, if both the exposure and outcome are negatively associated with selection, then this will bias the effect estimate downwards, so accounting for this results in a larger effect estimate here. Compared to the mother's results, the potential impact of data being Missing-Not-At-Random are more pronounced, because the amount of missing data in these variables is much larger (approx. 30% missing for religious attendance and 40% missing for blood donation); for a clearer presentation of these results for the confounders only adjustment set, see figure S12. CSP = Conditional Sensitivity Parameter; MSP = Marginal Sensitivity Parameter; IMOR = Ignorable Missingness Odds Ratio; Prev. = Prevalence; OR = Odds Ratio; CI = Confidence Interval; Out. = Outcome (blood donation); Exp. = Exposure (religious attendance).

CSP out.	*MSP out.*	*IMOR out.*	*Prev. out.*	*CSP exp.*	*MSP exp.*	*IMOR exp.*	*Prev. exp.*	**Unadjusted**				**Confounders only**				**Confounders and/or mediators**			
								OR	*Lower 95% CI*	*Upper 95% CI*	*p-value*	*OR*	*Lower 95% CI*	*Upper 95% CI*	*p-value*	*OR*	*Lower 95% CI*	*Upper 95% CI*	*p-value*
-2	-2.173	0.114	0.229	-2	-1.761	0.172	0.076	2.312	2.006	2.664	<0.001	1.670	1.438	1.941	<0.001	1.553	1.334	1.806	<0.001
-1.75	-1.924	0.146	0.235	-2	-1.747	0.174	0.076	2.291	1.986	2.643	<0.001	1.654	1.420	1.925	<0.001	1.540	1.320	1.797	<0.001
-1.5	-1.692	0.184	0.242	-2	-1.758	0.172	0.076	2.257	1.952	2.609	<0.001	1.632	1.399	1.904	<0.001	1.519	1.301	1.774	<0.001
-1.25	-1.442	0.236	0.251	-2	-1.795	0.166	0.075	2.217	1.917	2.560	<0.001	1.594	1.368	1.857	<0.001	1.489	1.279	1.735	<0.001
-1	-1.225	0.294	0.260	-2	-1.766	0.171	0.076	2.177	1.881	2.519	<0.001	1.570	1.344	1.833	<0.001	1.462	1.251	1.711	<0.001
-0.75	-0.979	0.376	0.272	-2	-1.762	0.172	0.076	2.125	1.846	2.450	<0.001	1.537	1.318	1.793	<0.001	1.435	1.229	1.675	<0.001
-0.5	-0.745	0.475	0.285	-2	-1.754	0.173	0.076	2.063	1.772	2.399	<0.001	1.492	1.271	1.752	<0.001	1.395	1.188	1.639	<0.001
-0.25	-0.523	0.593	0.300	-2	-1.781	0.168	0.076	1.996	1.726	2.307	<0.001	1.445	1.239	1.684	<0.001	1.354	1.158	1.582	<0.001
0	-0.294	0.745	0.318	-2	-1.731	0.177	0.076	1.916	1.645	2.230	<0.001	1.395	1.190	1.637	<0.001	1.307	1.113	1.536	0.001
-2	-2.173	0.114	0.229	-1.75	-1.557	0.211	0.077	2.264	1.964	2.612	<0.001	1.640	1.411	1.910	<0.001	1.525	1.311	1.775	<0.001
-1.75	-1.921	0.147	0.235	-1.75	-1.562	0.210	0.077	2.257	1.956	2.606	<0.001	1.629	1.394	1.904	<0.001	1.514	1.293	1.774	<0.001
-1.5	-1.691	0.184	0.242	-1.75	-1.550	0.212	0.077	2.228	1.933	2.570	<0.001	1.613	1.384	1.881	<0.001	1.502	1.287	1.754	<0.001

-1.25	-1.449	0.235	0.250	-1.75	-1.566	0.209	0.077	2.179	1.881	2.522	<0.001	1.570	1.343	1.833	<0.001	1.461	1.249	1.709	<0.001
-1	-1.213	0.297	0.260	-1.75	-1.564	0.209	0.077	2.132	1.850	2.455	<0.001	1.542	1.327	1.793	<0.001	1.441	1.236	1.677	<0.001
-0.75	-0.979	0.376	0.272	-1.75	-1.557	0.211	0.077	2.077	1.800	2.396	<0.001	1.505	1.296	1.749	<0.001	1.404	1.207	1.632	<0.001
-0.5	-0.755	0.470	0.285	-1.75	-1.578	0.206	0.077	2.048	1.775	2.363	<0.001	1.481	1.270	1.726	<0.001	1.384	1.184	1.619	<0.001
-0.25	-0.523	0.593	0.300	-1.75	-1.550	0.212	0.077	1.964	1.694	2.277	<0.001	1.425	1.218	1.667	<0.001	1.338	1.141	1.568	<0.001
0	-0.294	0.745	0.318	-1.75	-1.533	0.216	0.077	1.927	1.660	2.234	<0.001	1.409	1.202	1.652	<0.001	1.322	1.127	1.548	0.001
-2	-2.170	0.114	0.229	-1.5	-1.355	0.258	0.079	2.206	1.906	2.550	<0.001	1.602	1.373	1.870	<0.001	1.490	1.275	1.744	<0.001
-1.75	-1.908	0.148	0.236	-1.5	-1.340	0.262	0.079	2.186	1.889	2.529	<0.001	1.592	1.365	1.857	<0.001	1.483	1.269	1.732	<0.001
-1.5	-1.680	0.186	0.242	-1.5	-1.374	0.253	0.078	2.168	1.876	2.507	<0.001	1.576	1.350	1.839	<0.001	1.468	1.257	1.716	<0.001
-1.25	-1.450	0.235	0.250	-1.5	-1.363	0.256	0.079	2.155	1.865	2.492	<0.001	1.559	1.334	1.824	<0.001	1.454	1.241	1.704	<0.001
-1	-1.218	0.296	0.260	-1.5	-1.376	0.253	0.078	2.102	1.815	2.438	<0.001	1.527	1.306	1.784	<0.001	1.426	1.218	1.669	<0.001
-0.75	-0.977	0.377	0.272	-1.5	-1.357	0.257	0.079	2.069	1.788	2.394	<0.001	1.499	1.283	1.752	<0.001	1.404	1.200	1.642	<0.001
-0.5	-0.754	0.471	0.285	-1.5	-1.370	0.254	0.078	2.012	1.738	2.328	<0.001	1.459	1.249	1.706	<0.001	1.363	1.164	1.598	<0.001
-0.25	-0.533	0.587	0.300	-1.5	-1.344	0.261	0.079	1.968	1.706	2.273	<0.001	1.435	1.230	1.675	<0.001	1.346	1.150	1.575	<0.001
0	-0.298	0.742	0.317	-1.5	-1.348	0.260	0.079	1.902	1.640	2.203	<0.001	1.392	1.191	1.629	<0.001	1.307	1.114	1.533	0.001
-2	-2.155	0.116	0.230	-1.25	-1.169	0.311	0.080	2.138	1.859	2.460	<0.001	1.562	1.347	1.811	<0.001	1.455	1.252	1.690	<0.001
-1.75	-1.915	0.147	0.235	-1.25	-1.168	0.311	0.080	2.125	1.844	2.452	<0.001	1.556	1.332	1.815	<0.001	1.448	1.239	1.694	<0.001
-1.5	-1.684	0.186	0.242	-1.25	-1.143	0.319	0.081	2.113	1.826	2.447	<0.001	1.542	1.319	1.802	<0.001	1.436	1.226	1.682	<0.001
-1.25	-1.437	0.238	0.251	-1.25	-1.170	0.310	0.080	2.088	1.806	2.413	<0.001	1.519	1.302	1.772	<0.001	1.418	1.213	1.655	<0.001
-1	-1.204	0.300	0.261	-1.25	-1.141	0.319	0.081	2.061	1.786	2.377	<0.001	1.505	1.290	1.754	<0.001	1.405	1.202	1.640	<0.001
-0.75	-0.971	0.379	0.272	-1.25	-1.181	0.307	0.080	2.038	1.765	2.354	<0.001	1.489	1.275	1.740	<0.001	1.394	1.191	1.631	<0.001
-0.5	-0.746	0.474	0.285	-1.25	-1.147	0.318	0.081	1.986	1.711	2.305	<0.001	1.451	1.239	1.699	<0.001	1.359	1.161	1.592	<0.001
-0.25	-0.518	0.596	0.301	-1.25	-1.157	0.314	0.080	1.950	1.680	2.264	<0.001	1.423	1.212	1.674	<0.001	1.336	1.137	1.571	<0.001
0	-0.291	0.748	0.318	-1.25	-1.171	0.310	0.080	1.893	1.627	2.201	<0.001	1.391	1.186	1.629	<0.001	1.305	1.111	1.534	0.001
-2	-2.160	0.115	0.230	-1	-0.979	0.376	0.082	2.100	1.829	2.411	<0.001	1.539	1.326	1.786	<0.001	1.432	1.231	1.667	<0.001
-1.75	-1.924	0.146	0.235	-1	-0.981	0.375	0.082	2.083	1.791	2.425	<0.001	1.522	1.301	1.782	<0.001	1.419	1.212	1.662	<0.001
-1.5	-1.685	0.185	0.242	-1	-0.976	0.377	0.083	2.061	1.786	2.380	<0.001	1.508	1.293	1.758	<0.001	1.408	1.204	1.644	<0.001
-1.25	-1.432	0.239	0.251	-1	-0.988	0.372	0.082	2.036	1.749	2.370	<0.001	1.484	1.265	1.744	<0.001	1.387	1.178	1.631	<0.001
-1	-1.209	0.298	0.260	-1	-0.965	0.381	0.083	2.010	1.728	2.335	<0.001	1.468	1.250	1.725	<0.001	1.372	1.163	1.616	<0.001
-0.75	-0.977	0.377	0.272	-1	-0.955	0.385	0.083	1.998	1.730	2.305	<0.001	1.470	1.259	1.718	<0.001	1.374	1.176	1.606	<0.001
-0.5	-0.755	0.470	0.285	-1	-0.974	0.378	0.083	1.954	1.692	2.259	<0.001	1.438	1.235	1.674	<0.001	1.349	1.155	1.575	<0.001
-0.25	-0.524	0.592	0.300	-1	-0.981	0.375	0.082	1.935	1.660	2.252	<0.001	1.423	1.210	1.672	<0.001	1.332	1.132	1.570	0.001
0	-0.298	0.742	0.317	-1	-0.964	0.381	0.083	1.876	1.627	2.164	<0.001	1.387	1.195	1.608	<0.001	1.303	1.122	1.514	0.001
-2	-2.162	0.115	0.230	-0.75	-0.775	0.461	0.085	2.020	1.752	2.328	<0.001	1.489	1.275	1.737	<0.001	1.387	1.185	1.623	<0.001
-1.75	-1.912	0.148	0.236	-0.75	-0.777	0.460	0.085	2.008	1.745	2.309	<0.001	1.480	1.270	1.725	<0.001	1.379	1.182	1.608	<0.001
-1.5	-1.687	0.185	0.242	-0.75	-0.797	0.451	0.085	2.018	1.749	2.328	<0.001	1.490	1.284	1.732	<0.001	1.392	1.196	1.621	<0.001
-1.25	-1.450	0.235	0.250	-0.75	-0.797	0.451	0.085	1.984	1.714	2.298	<0.001	1.462	1.247	1.716	<0.001	1.368	1.164	1.606	<0.001
-1	-1.205	0.300	0.261	-0.75	-0.783	0.457	0.085	1.980	1.699	2.307	<0.001	1.459	1.237	1.719	<0.001	1.365	1.155	1.613	<0.001
-0.75	-0.982	0.374	0.272	-0.75	-0.784	0.456	0.085	1.970	1.697	2.286	<0.001	1.458	1.241	1.711	<0.001	1.363	1.162	1.600	<0.001

-0.5	-0.747	0.474	0.285	-0.75	-0.777	0.460	0.085	1.917	1.660	2.214	<0.001	1.415	1.213	1.650	<0.001	1.326	1.133	1.550	<0.001
-0.25	-0.512	0.599	0.301	-0.75	-0.763	0.466	0.085	1.885	1.632	2.175	<0.001	1.401	1.206	1.627	<0.001	1.317	1.131	1.531	<0.001
0	-0.282	0.754	0.319	-0.75	-0.751	0.472	0.086	1.840	1.584	2.138	<0.001	1.372	1.174	1.603	<0.001	1.289	1.102	1.507	0.002
-2	-2.160	0.115	0.230	-0.5	-0.613	0.542	0.088	1.962	1.706	2.257	<0.001	1.458	1.255	1.694	<0.001	1.358	1.168	1.579	<0.001
-1.75	-1.918	0.147	0.235	-0.5	-0.606	0.545	0.088	1.950	1.689	2.255	<0.001	1.451	1.247	1.689	<0.001	1.355	1.165	1.576	<0.001
-1.5	-1.673	0.188	0.242	-0.5	-0.614	0.541	0.088	1.944	1.684	2.248	<0.001	1.442	1.237	1.679	<0.001	1.344	1.153	1.568	<0.001
-1.25	-1.432	0.239	0.251	-0.5	-0.600	0.549	0.088	1.917	1.660	2.214	<0.001	1.420	1.221	1.650	<0.001	1.328	1.141	1.548	<0.001
-1	-1.206	0.299	0.261	-0.5	-0.607	0.545	0.088	1.925	1.657	2.237	<0.001	1.429	1.218	1.679	<0.001	1.339	1.141	1.571	<0.001
-0.75	-0.970	0.379	0.272	-0.5	-0.596	0.551	0.088	1.898	1.629	2.212	<0.001	1.409	1.201	1.655	<0.001	1.319	1.122	1.551	0.001
-0.5	-0.742	0.476	0.286	-0.5	-0.607	0.545	0.088	1.889	1.644	2.168	<0.001	1.408	1.214	1.632	<0.001	1.323	1.139	1.537	<0.001
-0.25	-0.524	0.592	0.300	-0.5	-0.605	0.546	0.088	1.850	1.597	2.143	<0.001	1.385	1.186	1.616	<0.001	1.300	1.111	1.519	0.001
0	-0.291	0.748	0.318	-0.5	-0.600	0.549	0.088	1.829	1.570	2.130	<0.001	1.370	1.169	1.606	<0.001	1.292	1.102	1.516	0.002
-2	-2.147	0.117	0.230	-0.25	-0.427	0.653	0.091	1.893	1.634	2.190	<0.001	1.418	1.217	1.652	<0.001	1.324	1.137	1.545	<0.001
-1.75	-1.925	0.146	0.235	-0.25	-0.428	0.652	0.091	1.863	1.616	2.145	<0.001	1.392	1.196	1.621	<0.001	1.300	1.115	1.516	0.001
-1.5	-1.681	0.186	0.242	-0.25	-0.437	0.646	0.091	1.885	1.627	2.184	<0.001	1.409	1.206	1.645	<0.001	1.315	1.125	1.539	0.001
-1.25	-1.433	0.239	0.251	-0.25	-0.431	0.650	0.091	1.861	1.598	2.168	<0.001	1.390	1.183	1.632	<0.001	1.300	1.104	1.530	0.002
-1	-1.205	0.300	0.261	-0.25	-0.418	0.658	0.091	1.857	1.624	2.125	<0.001	1.391	1.203	1.610	<0.001	1.302	1.123	1.510	<0.001
-0.75	-0.979	0.376	0.272	-0.25	-0.427	0.652	0.091	1.857	1.610	2.143	<0.001	1.390	1.186	1.627	<0.001	1.302	1.110	1.527	0.001
-0.5	-0.744	0.475	0.286	-0.25	-0.425	0.654	0.091	1.840	1.590	2.130	<0.001	1.380	1.186	1.605	<0.001	1.297	1.115	1.508	0.001
-0.25	-0.523	0.593	0.300	-0.25	-0.428	0.652	0.091	1.826	1.579	2.109	<0.001	1.374	1.182	1.600	<0.001	1.292	1.107	1.507	0.001
0	-0.301	0.740	0.317	-0.25	-0.405	0.667	0.092	1.813	1.559	2.106	<0.001	1.376	1.179	1.605	<0.001	1.297	1.111	1.513	0.001
-2	-2.176	0.114	0.229	0	-0.268	0.765	0.095	1.799	1.565	2.069	<0.001	1.354	1.163	1.575	<0.001	1.265	1.087	1.473	0.003
-1.75	-1.932	0.145	0.235	0	-0.268	0.765	0.095	1.806	1.565	2.081	<0.001	1.368	1.177	1.589	<0.001	1.278	1.100	1.484	0.001
-1.5	-1.672	0.188	0.243	0	-0.260	0.771	0.095	1.802	1.567	2.075	<0.001	1.361	1.170	1.581	<0.001	1.275	1.095	1.483	0.002
-1.25	-1.439	0.237	0.251	0	-0.251	0.778	0.095	1.811	1.571	2.088	<0.001	1.366	1.176	1.587	<0.001	1.279	1.100	1.487	0.001
-1	-1.201	0.301	0.261	0	-0.269	0.764	0.095	1.811	1.568	2.090	<0.001	1.365	1.171	1.589	<0.001	1.279	1.100	1.487	0.001
-0.75	-0.981	0.375	0.272	0	-0.260	0.771	0.095	1.818	1.576	2.096	<0.001	1.377	1.184	1.600	<0.001	1.290	1.112	1.499	0.001
-0.5	-0.738	0.478	0.286	0	-0.263	0.769	0.095	1.793	1.553	2.071	<0.001	1.362	1.168	1.590	<0.001	1.279	1.096	1.490	0.002
-0.25	-0.509	0.601	0.301	0	-0.259	0.772	0.095	1.790	1.537	2.083	<0.001	1.363	1.162	1.598	<0.001	1.285	1.092	1.513	0.003
0	-0.284	0.753	0.318	0	-0.251	0.778	0.095	1.759	1.527	2.028	<0.001	1.346	1.155	1.567	<0.001	1.269	1.088	1.478	0.002

Figure S1*:* Results of the complete-case (black) and multiple imputation (red) analyses for each religiosity exposure with blood donation as the outcome for mothers, comparing the predicted difference in blood donation for each model. The 'confounders only' scenario adjusts only for assumed confounders, while the 'confounders and/or mediators' scenario adjusts for both assumed confounders and variables which may be both confounders and mediators (see table 2 and figure 3). For odds ratios of these models, see figure 5.

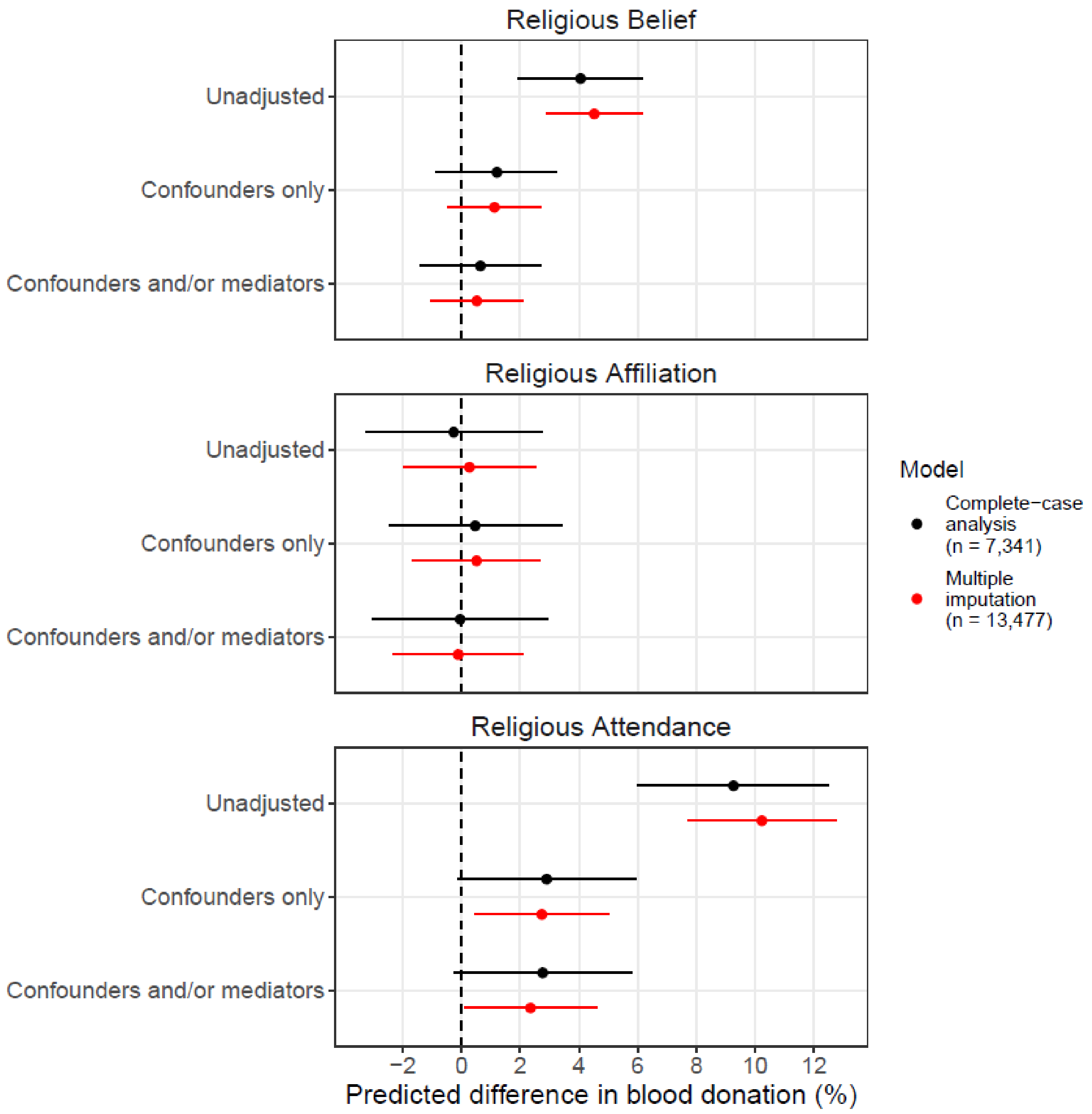

Figure S2*:* Results of the complete-case (black) and multiple imputation (red) analyses for each religiosity exposure with blood donation as the outcome for partners. The 'confounders only' scenario adjusts only for assumed confounders, while the 'confounders and/or mediators' scenario adjusts for both assumed confounders and variables which may be both confounders and mediators (see table 2 and figure 3). For differences in the probabilities of donating blood based on these models, see figure S3.

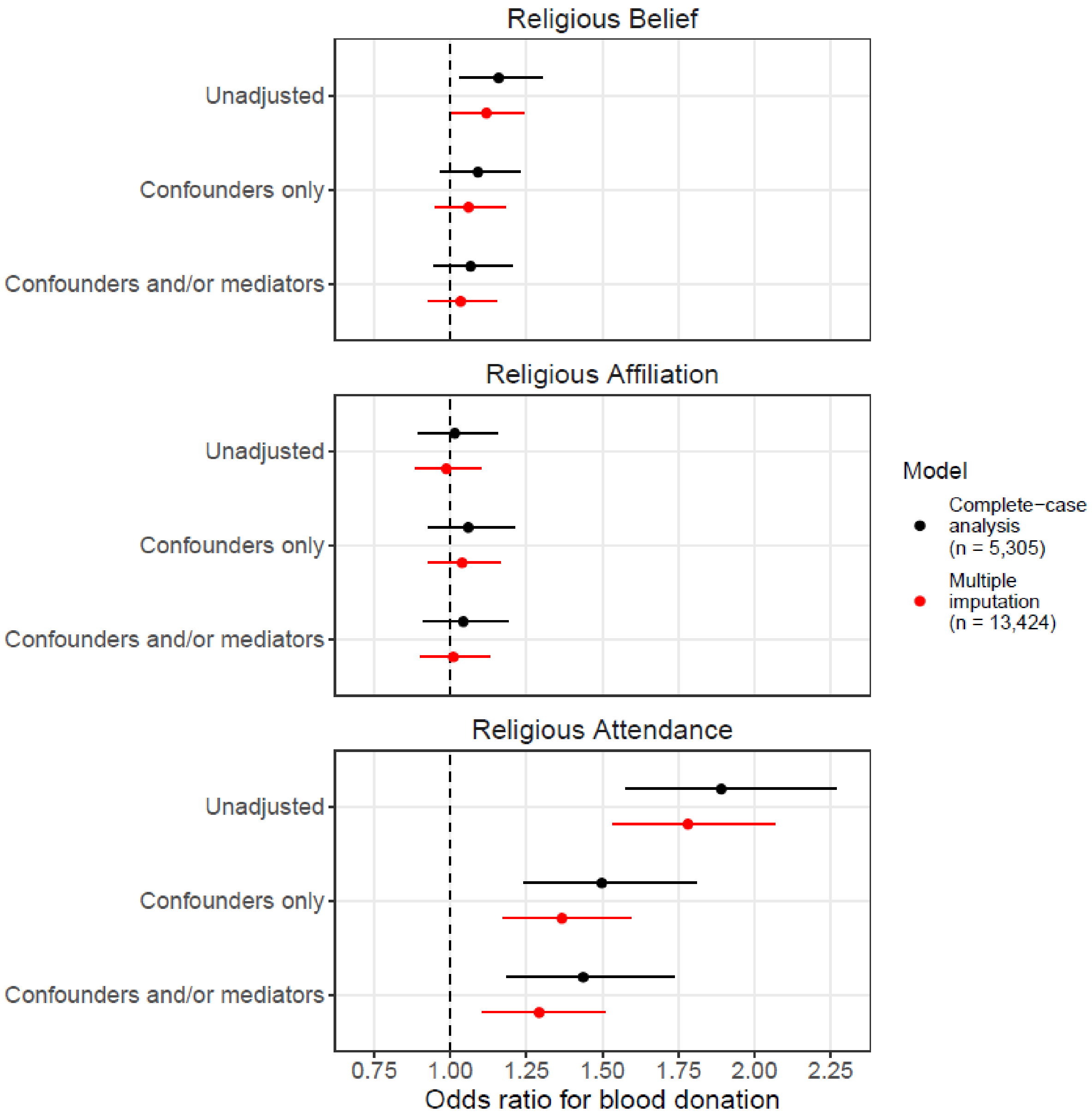

Figure S3*:* Results of the complete-case (black) and multiple imputation (red) analyses for each religiosity exposure with blood donation as the outcome for partners, comparing the predicted difference in blood donation for each model. The 'confounders only' scenario adjusts only for assumed confounders, while the 'confounders and/or mediators' scenario adjusts for both assumed confounders and variables which may be both confounders and mediators (see table 2 and figure 3). For odds ratios of these models, see figure S2.

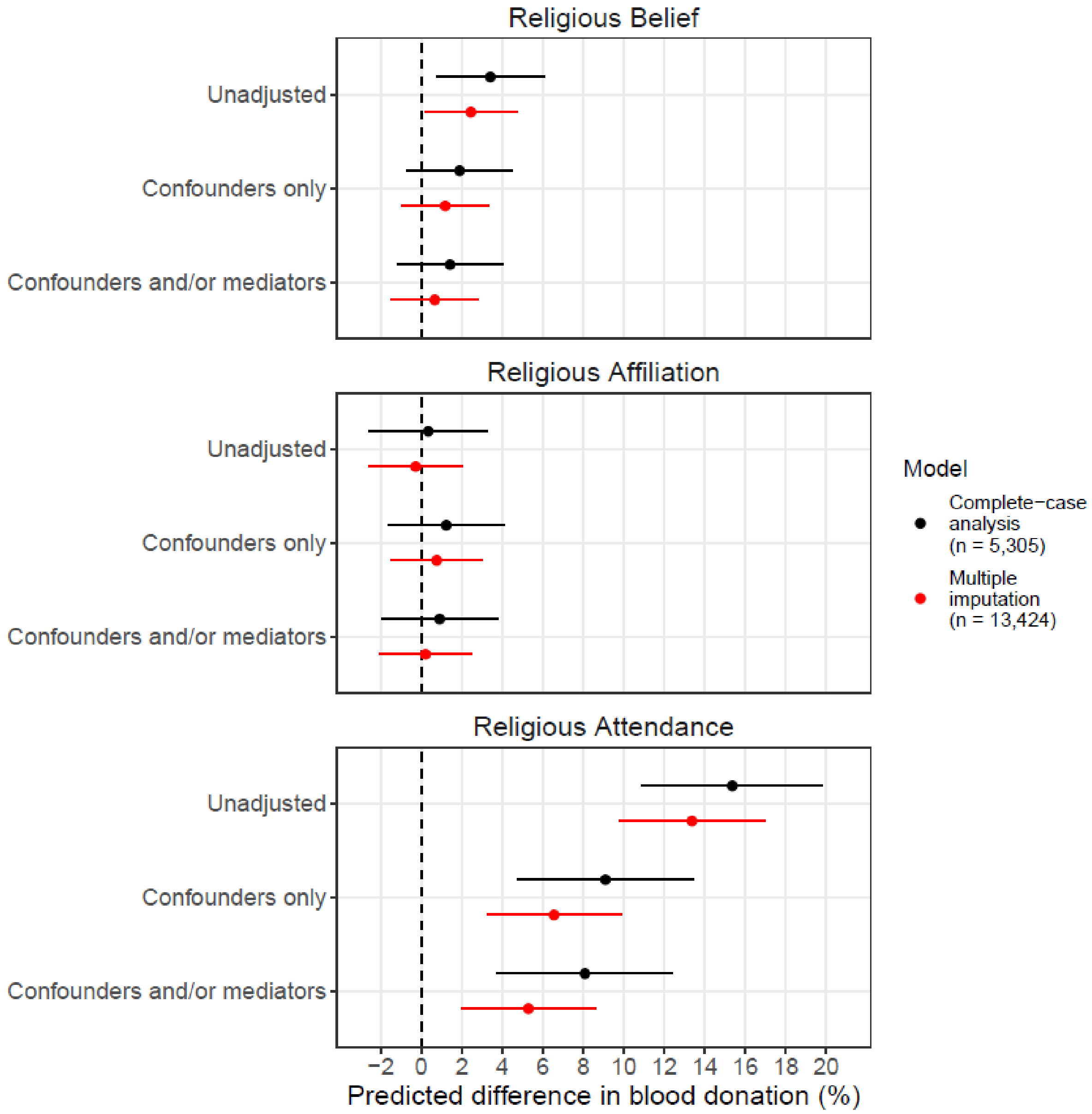

Figure S4*:* Results of the generalised sensitivity analysis in mothers to explain away the observed association between religious attendance and blood donation, when adjusted for 'confounders only' (odds ratio = 1.154). This analysis shows the association between a hypothetical unmeasured binary confounder and both the exposure (religious attendance) and the outcome (blood donation) necessary to reduce the observed association to target value; here, a null association (i.e., a *z*-statistic [or *t*-statistic] of 0.1, with a range between 0.0 and 0.2). Associations between the hypothetical confounder and exposure and outcome are measured in terms of partial correlation coefficients. The blue circles correspond to simulated parameters which meet the target value (i.e., a null association/*z*-statistic of 0.1), while the red line smooths through these points to denote the approximate levels of unmeasured confounding for a range of values necessary to reduce the observed association to the target. For instance, partial correlations of approximately 0.15 between the hypothetical unmeasured confounder and the exposure, and 0.10 between the hypothetical confounder and the outcome, would be sufficient to produce a null association between religious attendance and blood donation. The labels in black represent the partial correlations between some of the observed covariates and both the exposure and outcome, and are useful as a benchmark to compare the results against. For instance, the 'Degree' covariate is near the red line, meaning that an unmeasured confounder which has a similar association with both the exposure and outcome as degree-level education would be sufficient to remove the observed exposure-outcome association; this is perhaps possible, meaning that we should not have much confidence that the observed exposure-outcome association is robust to unmeasured confounding. Note, also, that this is quite an extreme scenario – reducing the observed association to null – and that ordinarily a shift in the observed association to a *t*- or *z*-statistic of 1.96 (i.e., no longer 'statistically significant'), or some other threshold, may be sufficient to question how robust the observed association is to unmeasured confounding; as the 95% confidence intervals of the observed association already cross the null, this type of analysis would not be feasible here. The results of this generalised sensitivity analysis also correspond well to those of the E-value approach, which indicate that relatively little unmeasured confounding is necessary to explain away the observed association.

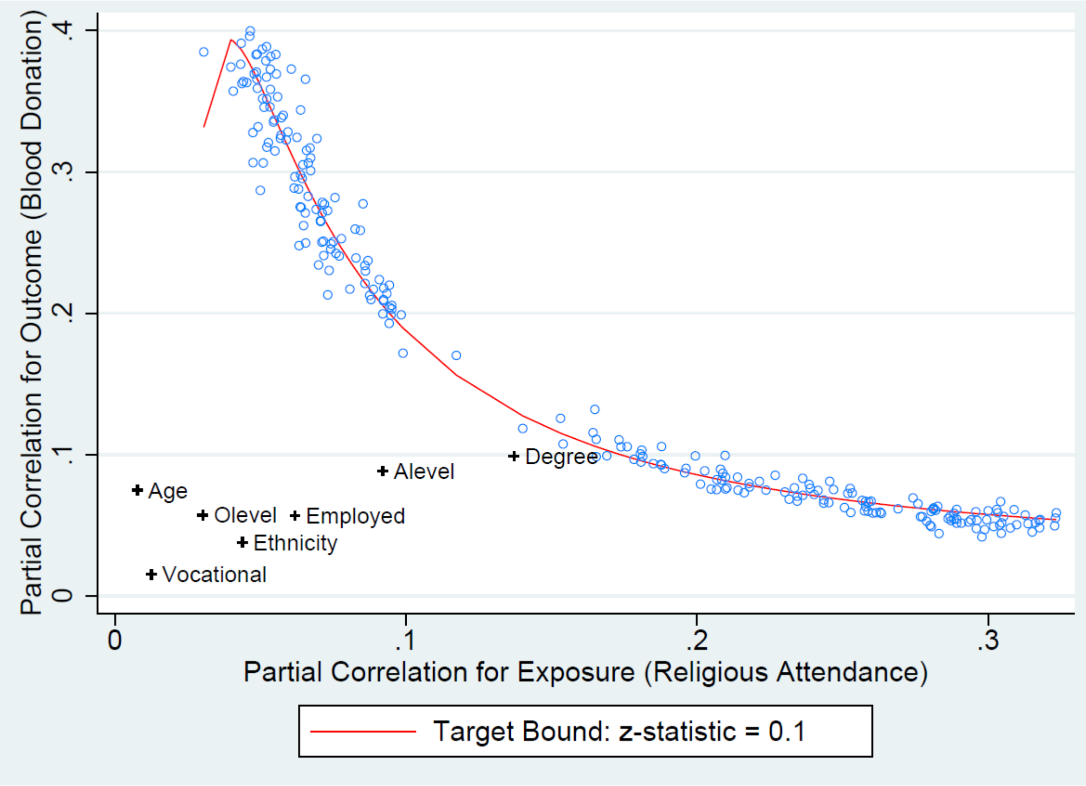

Figure S5*:* Results of the generalised sensitivity analysis in partners to explain away the observed association between religious attendance and blood donation, when adjusted for 'confounders only' (odds ratio = 1.498). This analysis shows the association between a hypothetical unmeasured binary confounder and both the exposure (religious attendance) and the outcome (blood donation) necessary to reduce the observed association to target value; here, a null association (i.e., a *z*-statistic of 0.1, with a range between 0.0 and 0.2). For a more detailed interpretation of this plot, see the legend to figure S4. Here, partial correlations of approximately 0.25 between the hypothetical unmeasured confounder and the exposure, and 0.20 between the hypothetical confounder and the outcome, would be sufficient to produce a null association between religious attendance and blood donation. This is a much larger effect than for the mother's data (figure S4), corroborated by the partial correlations between some of the observed covariates (in black) being more distant from the red boundary line. We can therefore have more confidence in this result, as greater levels of unmeasured confounding are necessary to reduce the observed association to null, also supporting the E-values results presented in text. However, this is the level of unmeasured confounding necessary for a strict null; in figure S6 below we repeat this analysis, this time using a *z*-statistic of 1.96 (i.e., no longer 'statistically significant', at an alpha level of 0.05) as our target.

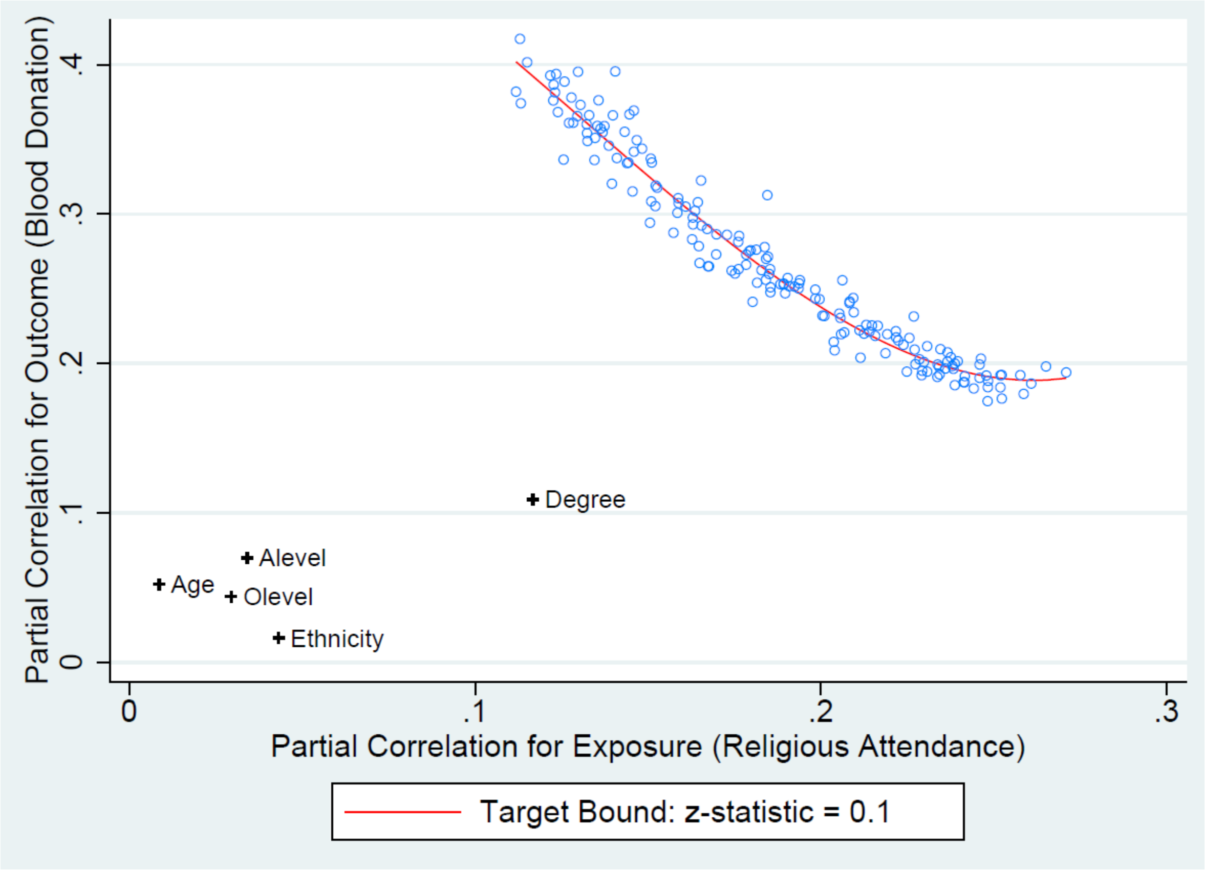

Figure S6*:* Results of the generalised sensitivity analysis in partners to explain away the observed association between religious attendance and blood donation, when adjusted for 'confounders only' (odds ratio = 1.498). This analysis shows the association between a hypothetical unmeasured binary confounder and both the exposure (religious attendance) and the outcome (blood donation) necessary to reduce the observed association to target value; here, a *z*-statistic of 1.96, meaning the association between the exposure and outcome is no longer 'statistically significant' at a standard 0.05 alpha level. For a more detailed interpretation of this plot, see the legend to figure S4. Here, partial correlations of approximately 0.15 between the hypothetical unmeasured confounder and both the exposure and the outcome would be sufficient to produce a 'non-significant' association between religious attendance and blood donation. This is much smaller than the effect needed to produce a strict null association from these data (figure S5). It is also only marginally larger than the observed association between the covariate 'Degree' and the exposure and outcome (benchmarked in black), indicating that an unmeasured confound of this magnitude could perhaps be plausible. The evidence for a potential causal association between religious attendance and blood donation is therefore stronger in partners compared to mothers, although cannot realistically be ruled out; this interpretation also only focuses on unmeasured confounding, and ignores other sources of bias such as selection bias.

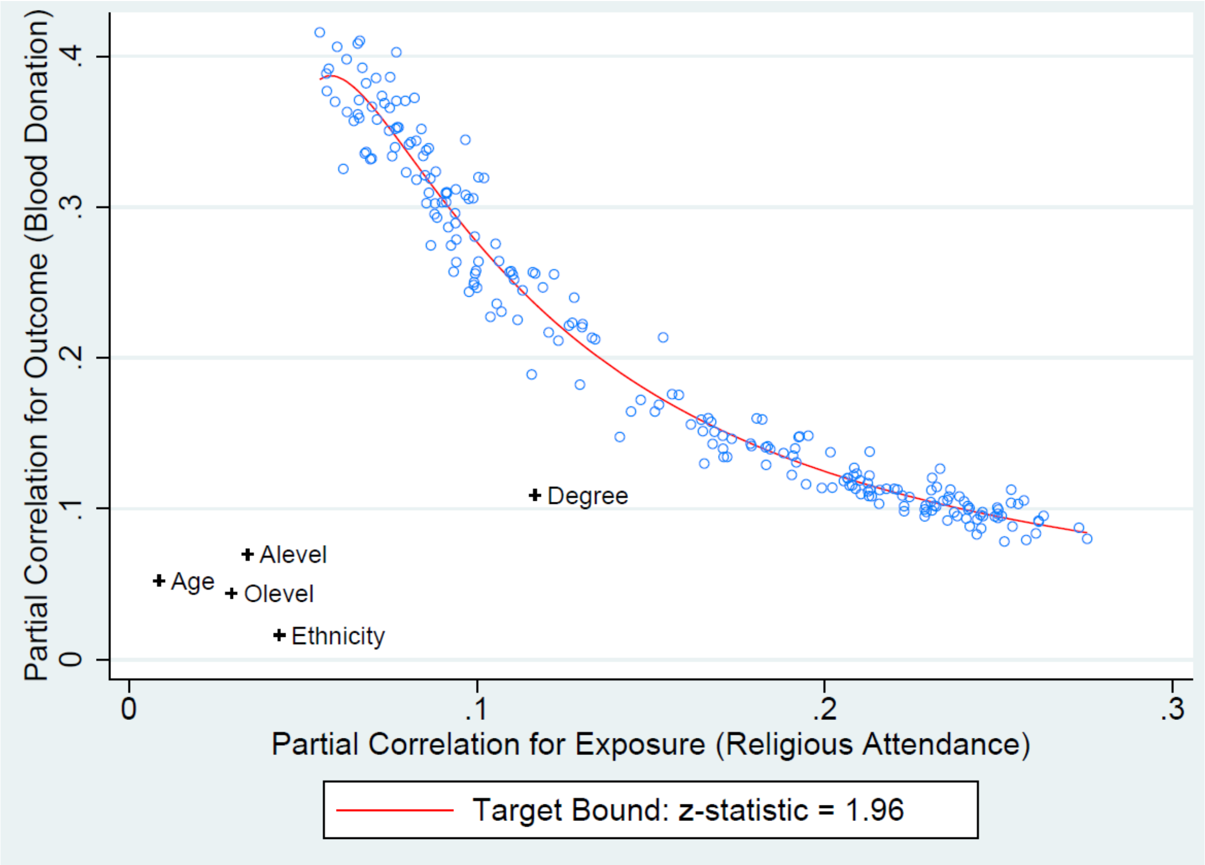

Figure S7: Results of the Not-At-Random Multiple Imputation analyses for mothers, assuming that just the exposure 'religious attendance' is Missing-Not-At-Random (that is, individuals with missing religious attendance data were less likely to attend a place of worship regularly; *n* = 13,477). This Not-At-Random Multiple Imputation sensitivity analysis compares the results of different 'Conditional Sensitivity Parameters', which are the extent to which those with missing data differ to those with observed data, conditional on all other variables in the imputation model. This is on the log-odds scale, which ranged from -2 to 0 in steps of 0.25 (x-axis). The odds ratio effect estimate of the exposure (regular religious attendance) on the outcome (blood donation) for these different conditional sensitivity parameters is given in blue, with lower and upper 95% confidence intervals in dashed green and red, respectively. Results are displayed for the 'confounders only' adjustment scenario, as the results of the 'confounders and/or mediators' scenario were practically identical. The vertical red line denotes a conditional sensitivity parameter of 0 (i.e., data Missing-At-Random). The horizontal red line denotes a null association (odds ratio = 1). To aid interpretation, these conditional sensitivity parameters have been converted to 'marginal sensitivity parameters' (that is, the marginal difference between those with vs without data, on the log-odds scale), 'ignorable missingness odds ratios' (that is, the marginal difference between those with vs without missing data, on the odds ratio scale) and prevalence estimates in the sample in table S9. For each conditional sensitivity parameter, we generated 50 imputations with a burn-in period of 10 iterations. Other than the addition of the sensitivity parameter, the imputation model was identical to that of the standard multiple imputation analysis. In this analysis, there is little variation in the effect estimates, suggesting that results are unlikely to be biased by selection if the exposure is Missing-Not-At-Random.

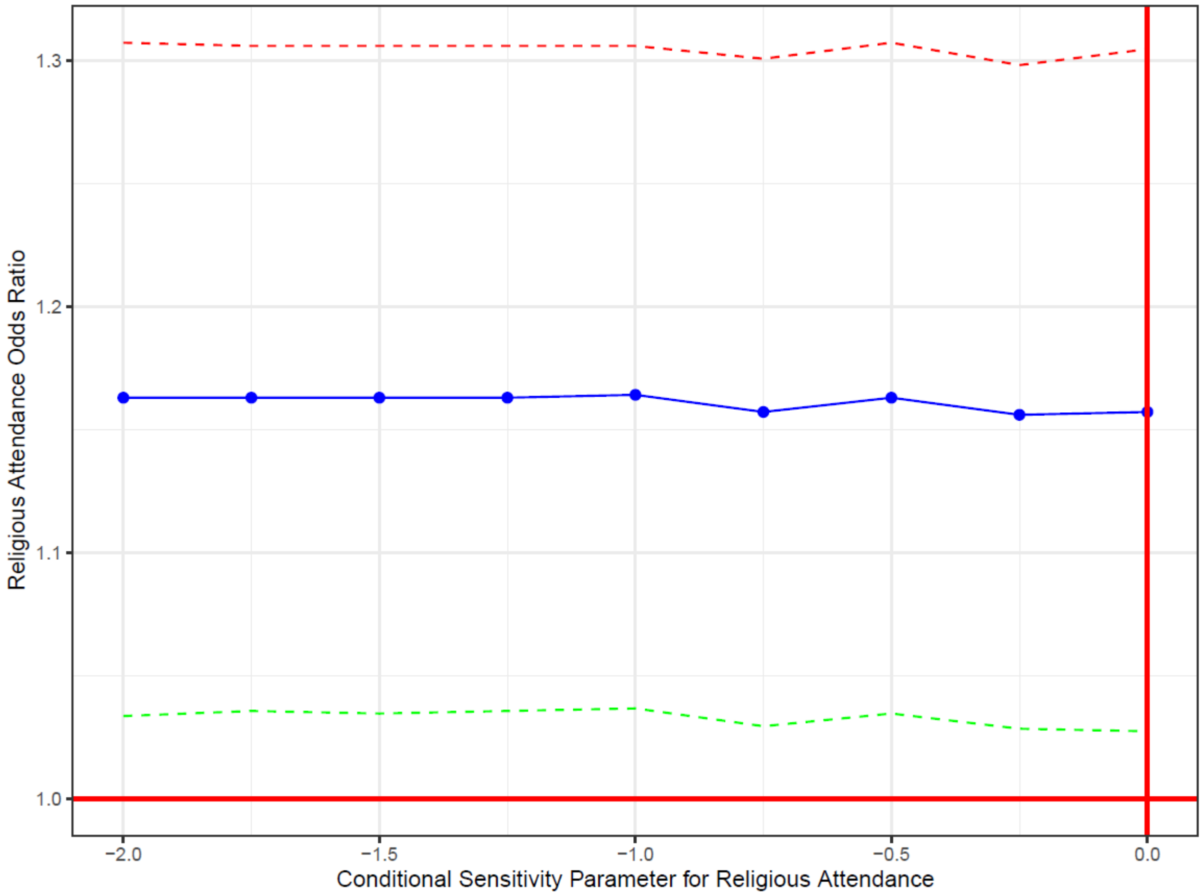

Figure S8: Results of the Not-At-Random Multiple Imputation analyses for mothers, assuming that just the outcome 'blood donation' is Missing-Not-At-Random (that is, individuals with missing blood donation data were less likely to donate blood; *n* = 13,477). This Not-At-Random Multiple Imputation sensitivity analysis compares the results of different 'Conditional Sensitivity Parameters', which are the extent to which those with missing data differ to those with observed data, conditional on all other variables in the imputation model. This is on the log-odds scale, which ranged from -2 to 0 in steps of 0.25 (x-axis). The odds ratio effect estimate of the exposure (regular religious attendance) on the outcome (blood donation) for these different conditional sensitivity parameters is given in blue, with lower and upper 95% confidence intervals in dashed green and red, respectively. Results are displayed for the 'confounders only' adjustment scenario, as the results of the 'confounders and/or mediators' scenario were practically identical. The vertical red line denotes a conditional sensitivity parameter of 0 (i.e., data Missing-At-Random). The horizontal red line denotes a null association (odds ratio = 1). To aid interpretation, these conditional sensitivity parameters have been converted to 'marginal sensitivity parameters' (that is, the marginal difference between those with vs without data, on the log-odds scale), 'ignorable missingness odds ratios' (that is, the marginal difference between those with vs without missing data, on the odds ratio scale) and prevalence estimates in the sample in table S10. For each conditional sensitivity parameter, we generated 50 imputations with a burn-in period of 10 iterations. Other than the addition of the sensitivity parameter, the imputation model was identical to that of the standard multiple imputation analysis. In this analysis, there is little variation in the effect estimates, suggesting that results are unlikely to be biased by selection if the outcome is Missing-Not-At-Random.

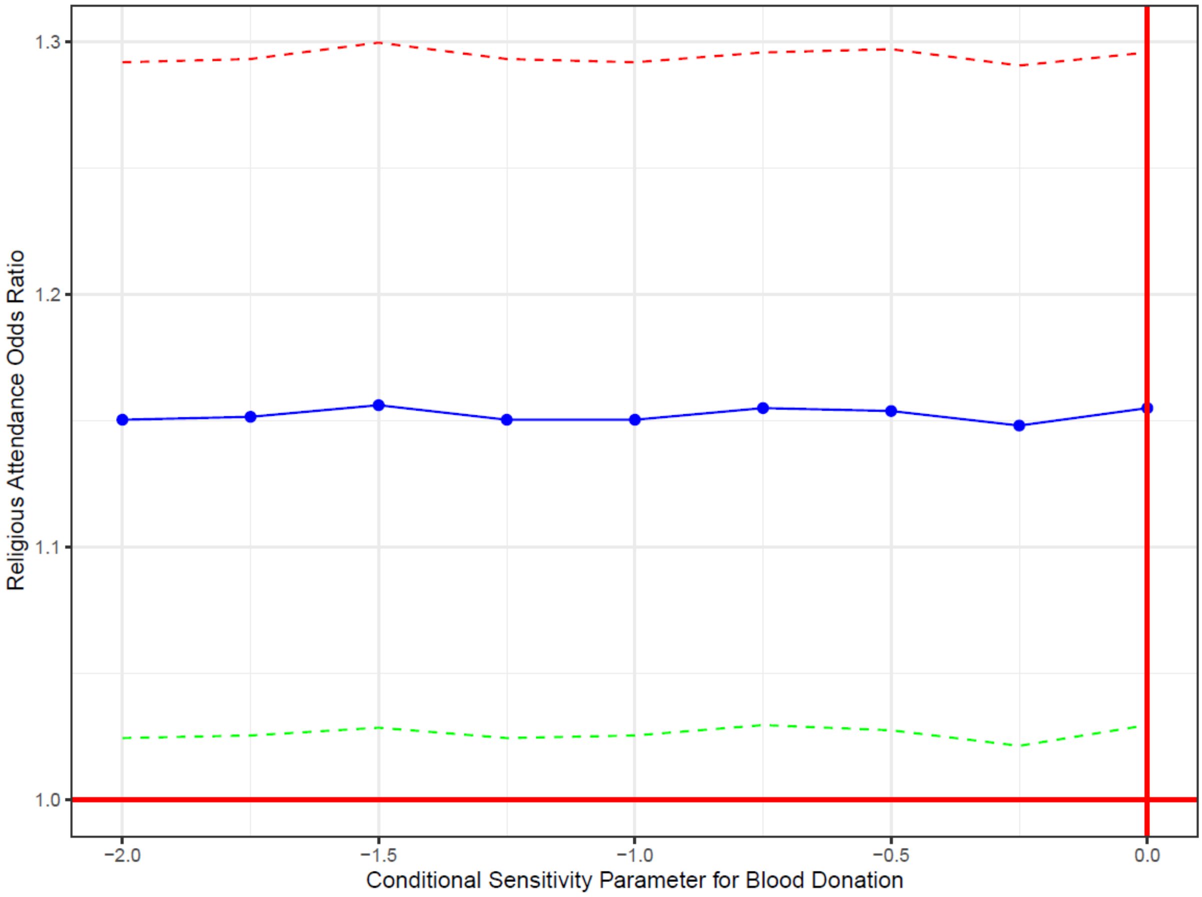

Figure S9: Results of the Not-At-Random Multiple Imputation analyses for mothers, assuming that both the exposure 'religious attendance' and the outcome 'blood donation' are Missing-Not-At-Random (that is, individuals with missing religious attendance data were less likely to attend a place of worship regularly *and* individuals with missing blood donation data were less likely to donate blood; *n* = 13,477). This Not-At-Random Multiple Imputation sensitivity analysis compares the results of different 'Conditional Sensitivity Parameters', which are the extent to which those with missing data differ to those with observed data, conditional on all other variables in the imputation model. This is on the log-odds scale, which ranged from -2 to 0 in steps of 0.25, looping over both the exposure and the outcome. Results are the odds ratio effect estimates of the exposure (regular religious attendance) on the outcome (blood donation) for the 'confounders only' adjustment scenario; only these results are displayed as the results of the 'confounders and/or mediators' scenario were practically identical. To aid interpretation, these conditional sensitivity parameters have been converted to 'marginal sensitivity parameters' (that is, the marginal difference between those with vs without data, on the log-odds scale), 'ignorable missingness odds ratios' (that is, the marginal difference between those with vs without missing data, on the odds ratio scale) and prevalence estimates in the sample in table S11. For each conditional sensitivity parameter, we generated 50 imputations with a burn-in period of 10 iterations. Other than the addition of the sensitivity parameters, the imputation model was identical to that of the standard multiple imputation analysis.

Figure 9a presents a heat-map of the odds ratio estimates for each combination of conditional sensitivity parameters for the exposure and outcome, with larger values a deeper shade of red. For instance, if the conditional sensitivity parameter was -2 for both the exposure and outcome, the odds ratio was 1.26; while if the conditional sensitivity parameter was 0 for both the exposure and outcome (i.e., neither variable Missing-Not-At-Random), the odds ratio was 1.16.

Figure 9b presents a heat-map of the lower 95% confidence intervals of the odds ratio estimates for each combination of conditional sensitivity parameters for the exposure and outcome, with larger values a deeper shade of red. For instance, if the conditional sensitivity parameter was -2 for both the exposure and outcome, the lower 95% confidence interval of the odds ratio was 1.13; while if the conditional sensitivity parameter was 0 for both the exposure and outcome (i.e., neither variable Missing-Not-At-Random), the lower 95% confidence interval of the odds ratio was 1.03.

Figure 9c presents three plots with the blood donation conditional sensitivity parameter on the x-axis, with each plot a different conditional sensitivity parameter for religious attendance (at -2, -1 and 0, respectively). The odds ratio effect estimate of the exposure (regular religious attendance) on the outcome (blood donation) for these different conditional sensitivity parameters is given in blue, with lower and upper 95% confidence intervals in dashed green and red, respectively.

In this analysis, if both the exposure and outcome are Missing-Not-At-Random, then the true effect estimate is larger than the observed effect estimate in the complete-case and standard multiple imputation analyses; this is because, if both the exposure and outcome are negatively associated with selection, then this will bias the effect estimate downwards, so accounting for this results in a larger effect estimate here. These differences are relatively minor however, likely because the amount of missing data in these variables is quite small (approx. 12% in both). For instance, although the odds ratio when both the exposure and outcome conditional sensitivity parameter are both -2 is larger than when the blood donation conditional sensitivity parameter is -2 and the religious attendance parameter is 0, overall the results are broadly similar and the confidence intervals overlap.

Figure 9a: Heat-map of the odds ratio estimates for each combination of conditional sensitivity parameters for the exposure and outcome, with larger values a deeper shade of red.

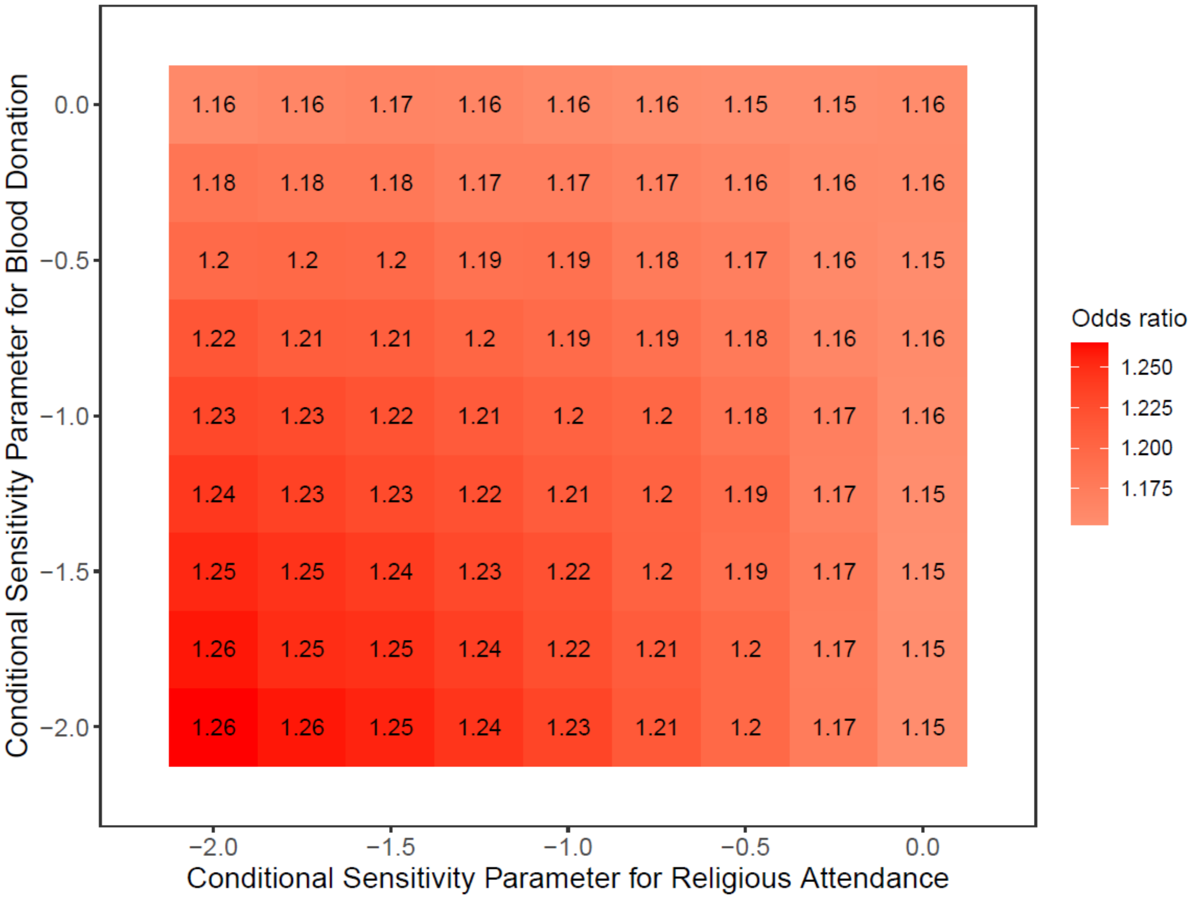

Figure 9b: heat-map of the lower 95% confidence intervals (CI) of the odds ratio (OR) estimates for each combination of conditional sensitivity parameters for the exposure and outcome, with larger values a deeper shade of red.

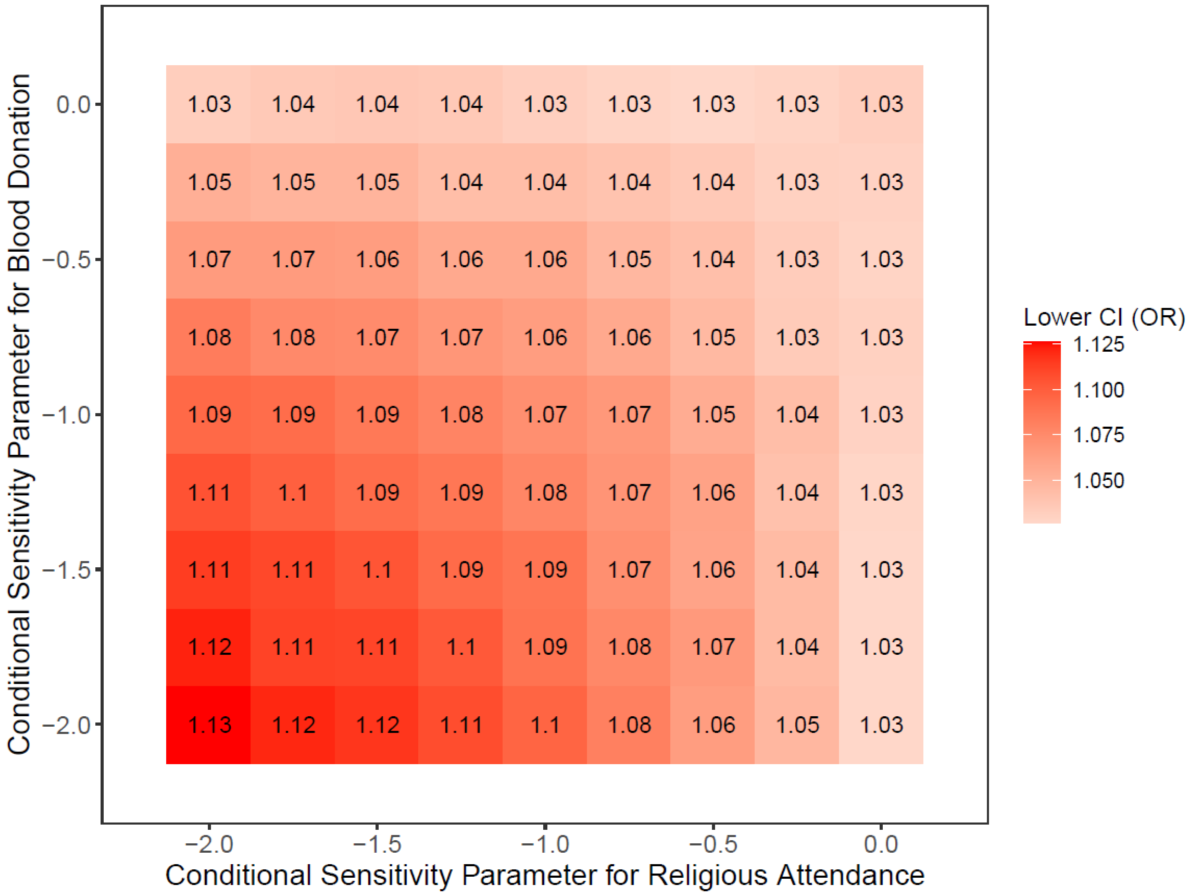

Figure 9c: Three plots with the blood donation conditional sensitivity parameter on the x-axis, with each plot a different conditional sensitivity parameter for religious attendance (at -2, -1 and 0, respectively). The odds ratio effect estimate of the exposure (regular religious attendance) on the outcome (blood donation) for these different conditional sensitivity parameters is given in blue, with lower and upper 95% confidence intervals in dashed green and red, respectively. The vertical red lines denote a conditional sensitivity parameter of 0 for the blood donation variable (i.e., data Missing-At-Random). The horizontal red lines denote a null association (odds ratio = 1).

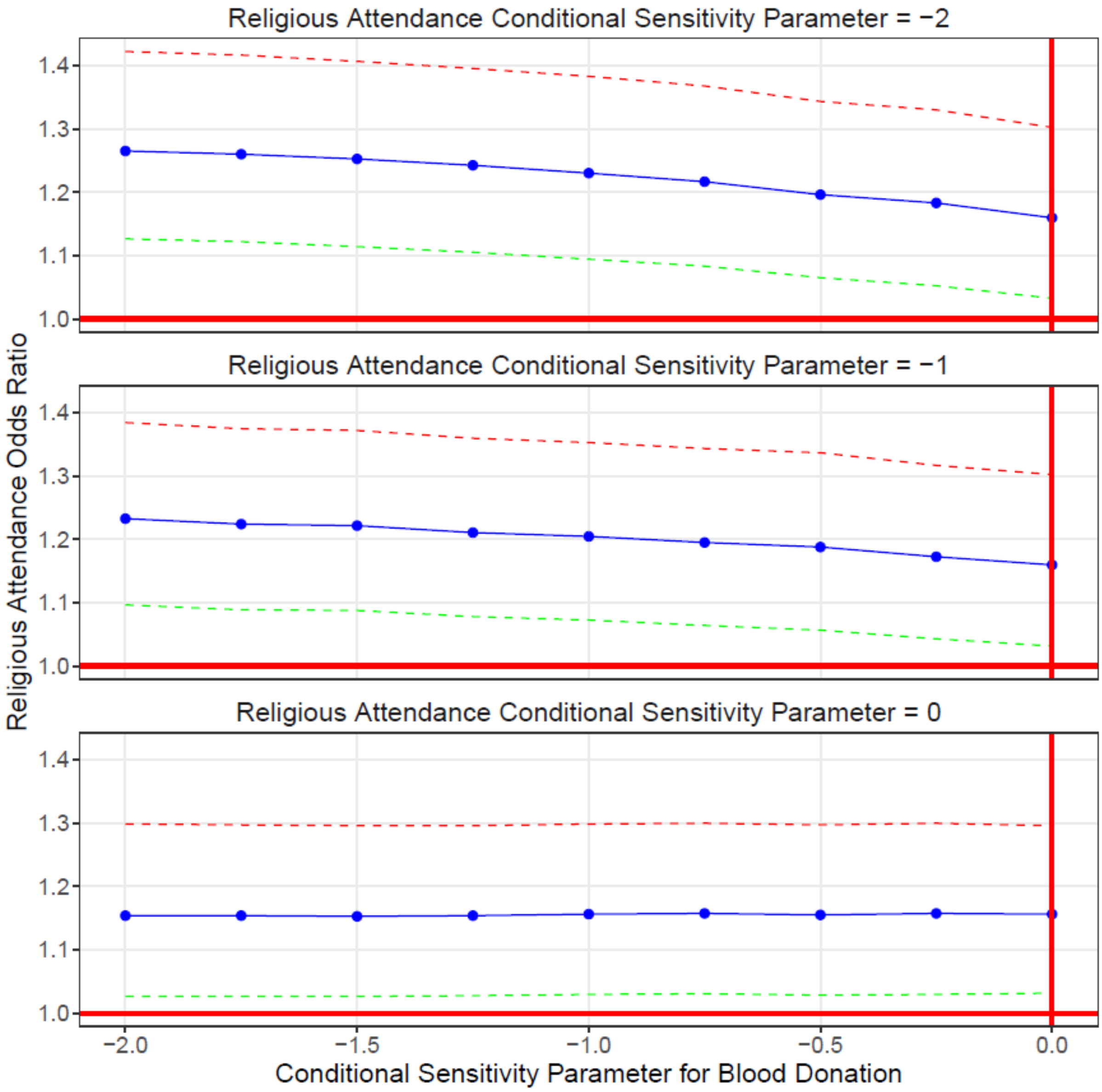

Figure S10: Results of the Not-At-Random Multiple Imputation analyses for partners, assuming that just the exposure 'religious attendance' is Missing-Not-At-Random (that is, individuals with missing religious attendance data were less likely to attend a place of worship regularly; *n* = 13,424). This Not-At-Random Multiple Imputation sensitivity analysis compares the results of different 'Conditional Sensitivity Parameters', which are the extent to which those with missing data differ to those with observed data, conditional on all other variables in the imputation model. This is on the log-odds scale, which ranged from -2 to 0 in steps of 0.25 (x-axis). The odds ratio effect estimate of the exposure (regular religious attendance) on the outcome (blood donation) for these different conditional sensitivity parameters is given in blue, with lower and upper 95% confidence intervals in dashed green and red, respectively. Results are displayed for the 'confounders only' adjustment scenario, as the results of the 'confounders and/or mediators' scenario were practically identical. The vertical red line denotes a conditional sensitivity parameter of 0 (i.e., data Missing-At-Random). The horizontal red line denotes a null association (odds ratio = 1). To aid interpretation, these conditional sensitivity parameters have been converted to 'marginal sensitivity parameters' (that is, the marginal difference between those with vs without data, on the log-odds scale), 'ignorable missingness odds ratios' (that is, the marginal difference between those with vs without missing data, on the odds ratio scale) and prevalence estimates in the sample in table S12. For each conditional sensitivity parameter, we generated 50 imputations with a burn-in period of 10 iterations. Other than the addition of the sensitivity parameter, the imputation model was identical to that of the standard multiple imputation analysis. In this analysis, there is little variation in the effect estimates, suggesting that results are unlikely to be biased by selection if the exposure is Missing-Not-At-Random.

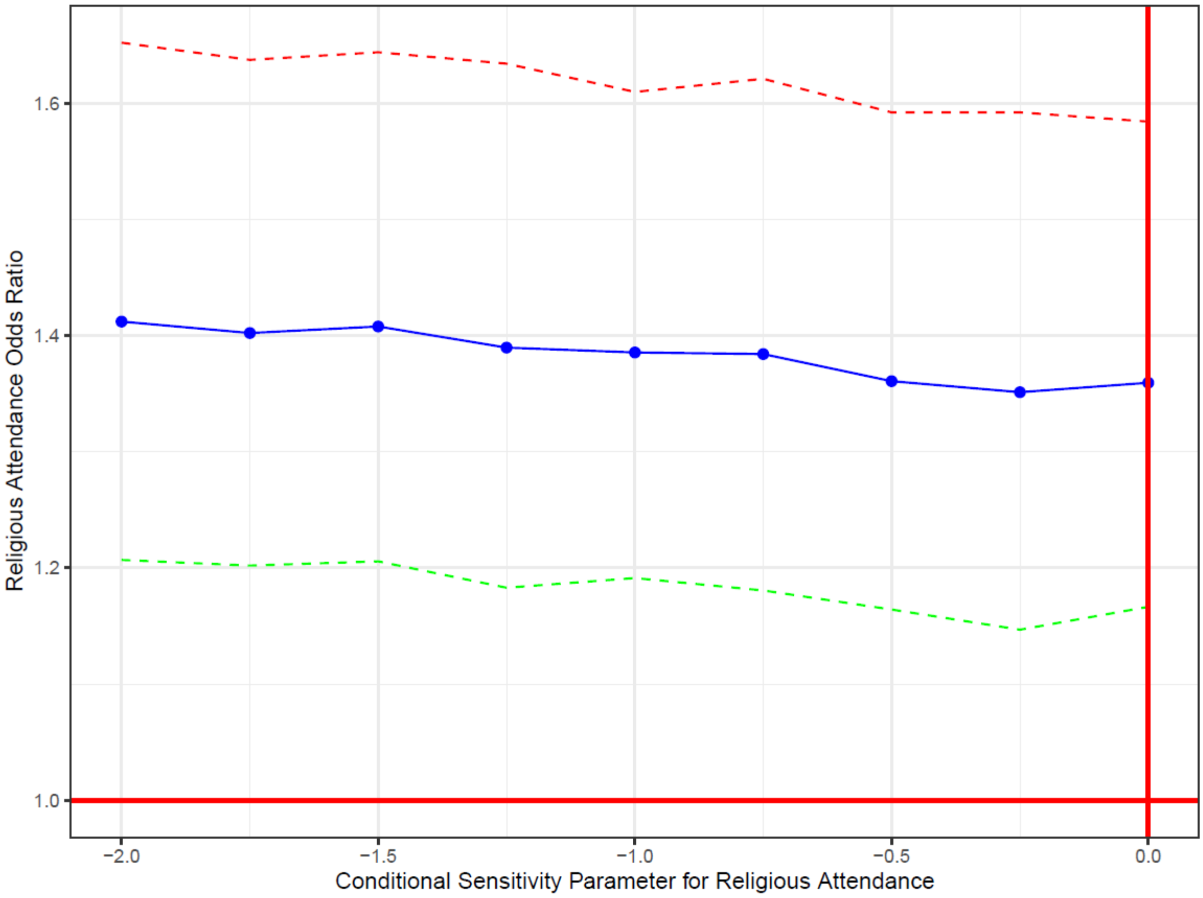

Figure S11: Results of the Not-At-Random Multiple Imputation analyses for partners, assuming that just the outcome 'blood donation' is Missing-Not-At-Random (that is, individuals with missing blood donation data were less likely to donate blood; *n* = 13,424). This Not-At-Random Multiple Imputation sensitivity analysis compares the results of different 'Conditional Sensitivity Parameters', which are the extent to which those with missing data differ to those with observed data, conditional on all other variables in the imputation model. This is on the log-odds scale, which ranged from -2 to 0 in steps of 0.25 (x-axis). The odds ratio effect estimate of the exposure (regular religious attendance) on the outcome (blood donation) for these different conditional sensitivity parameters is given in blue, with lower and upper 95% confidence intervals in dashed green and red, respectively. Results are displayed for the 'confounders only' adjustment scenario, as the results of the 'confounders and/or mediators' scenario were practically identical. The vertical red line denotes a conditional sensitivity parameter of 0 (i.e., data Missing-At-Random). The horizontal red line denotes a null association (odds ratio = 1). To aid interpretation, these conditional sensitivity parameters have been converted to 'marginal sensitivity parameters' (that is, the marginal difference between those with vs without data, on the log-odds scale), 'ignorable missingness odds ratios' (that is, the marginal difference between those with vs without missing data, on the odds ratio scale) and prevalence estimates in the sample in table S13. For each conditional sensitivity parameter, we generated 50 imputations with a burn-in period of 10 iterations. Other than the addition of the sensitivity parameter, the imputation model was identical to that of the standard multiple imputation analysis. In this analysis, there is little variation in the effect estimates, suggesting that results are unlikely to be biased by selection if the outcome is Missing-Not-At-Random.

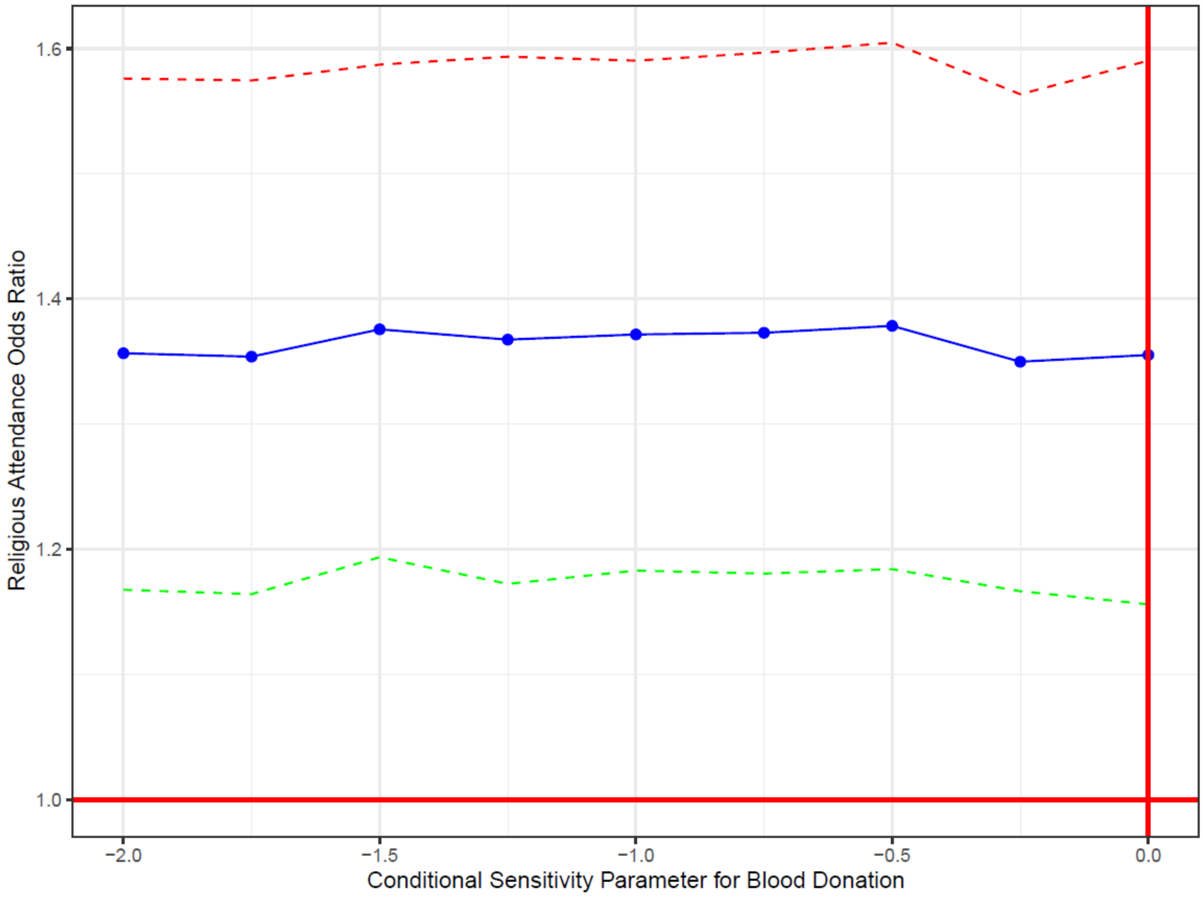

Figure S12: Results of the Not-At-Random Multiple Imputation analyses for partners, assuming that both the exposure 'religious attendance' and the outcome 'blood donation' are Missing-Not-At-Random (that is, individuals with missing religious attendance data were less likely to attend a place of worship regularly *and* individuals with missing blood donation data were less likely to donate blood; *n* = 13,424). This Not-At-Random Multiple Imputation sensitivity analysis compares the results of different 'Conditional Sensitivity Parameters', which are the extent to which those with missing data differ to those with observed data, conditional on all other variables in the imputation model. This is on the log-odds scale, which ranged from -2 to 0 in steps of 0.25, looping over both the exposure and the outcome. Results are the odds ratio effect estimates of the exposure (regular religious attendance) on the outcome (blood donation) for the 'confounders only' adjustment scenario; only these results are displayed as the results of the 'confounders and/or mediators' scenario were practically identical. To aid interpretation, these conditional sensitivity parameters have been converted to 'marginal sensitivity parameters' (that is, the marginal difference between those with vs without data, on the log-odds scale), 'ignorable missingness odds ratios' (that is, the marginal difference between those with vs without missing data, on the odds ratio scale) and prevalence estimates in the sample in table S14. For each conditional sensitivity parameter, we generated 50 imputations with a burn-in period of 10 iterations. Other than the addition of the sensitivity parameters, the imputation model was identical to that of the standard multiple imputation analysis.

Figure 12a presents a heat-map of the odds ratio estimates for each combination of conditional sensitivity parameters for the exposure and outcome, with larger values a deeper shade of red. For instance, if the conditional sensitivity parameter was -2 for both the exposure and outcome, the odds ratio was 1.67; while if the conditional sensitivity parameter was 0 for both the exposure and outcome (i.e., neither variable Missing-Not-At-Random), the odds ratio was 1.35.

Figure 12b presents a heat-map of the lower 95% confidence intervals of the odds ratio estimates for each combination of conditional sensitivity parameters for the exposure and outcome, with larger values a deeper shade of red. For instance, if the conditional sensitivity parameter was -2 for both the exposure and outcome, the lower 95% confidence interval of the odds ratio was 1.44; while if the conditional sensitivity parameter was 0 for both the exposure and outcome (i.e., neither variable Missing-Not-At-Random), the lower 95% confidence interval of the odds ratio was 1.15.

Figure 12c presents three plots with the blood donation conditional sensitivity parameter on the x-axis, with each plot a different conditional sensitivity parameter for religious attendance (at -2, -1 and 0, respectively). The odds ratio effect estimate of the exposure (regular religious attendance) on the outcome (blood donation) for these different conditional sensitivity parameters is given in blue, with lower and upper 95% confidence intervals in dashed green and red, respectively.

In this analysis, if both the exposure and outcome are Missing-Not-At-Random, then the true effect estimate is larger than the observed effect estimate in the complete-case and standard multiple imputation analyses; this is because, if both the exposure and outcome are negatively associated with selection, then this will bias the effect estimate downwards, so accounting for this results in a larger effect estimate here. Compared to the mother's results, the potential impact of data being Missing-Not-At-Random are more pronounced, because the amount of missing data in these variables is much larger (approx. 30% missing for religious attendance and 40% missing for blood donation). For instance, the odds ratio when both the exposure and outcome conditional sensitivity parameter are both -2 (odds ratio = 1.67) is much larger than when the blood donation conditional sensitivity parameter is -2 and the religious attendance parameter is 0 (odds ratio = 1.35), and the 95% confidence intervals do not overlap these odds ratio estimates.

Figure 12a: Heat-map of the odds ratio estimates for each combination of conditional sensitivity parameters for the exposure and outcome, with larger values a deeper shade of red.

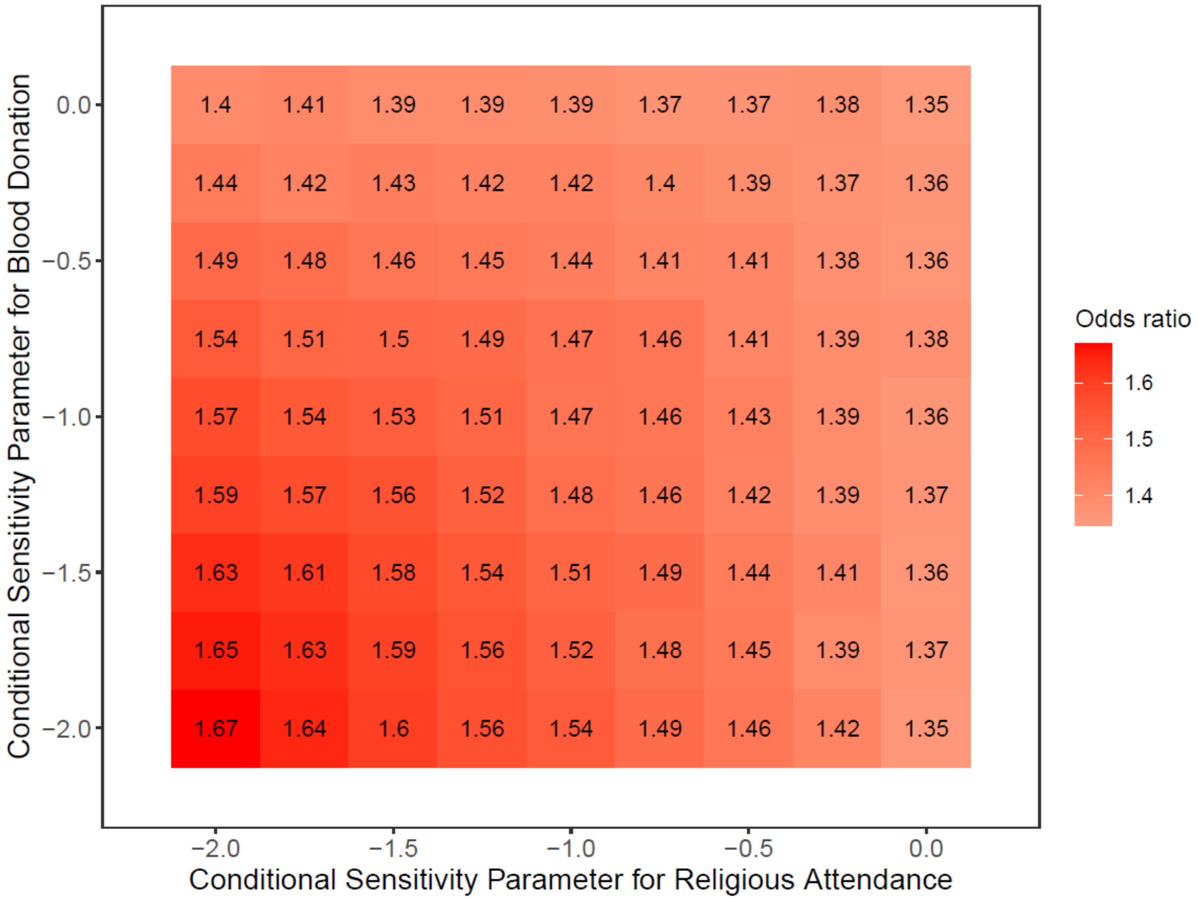

Figure 12b: heat-map of the lower 95% confidence intervals (CI) of the odds ratio (OR) estimates for each combination of conditional sensitivity parameters for the exposure and outcome, with larger values a deeper shade of red.

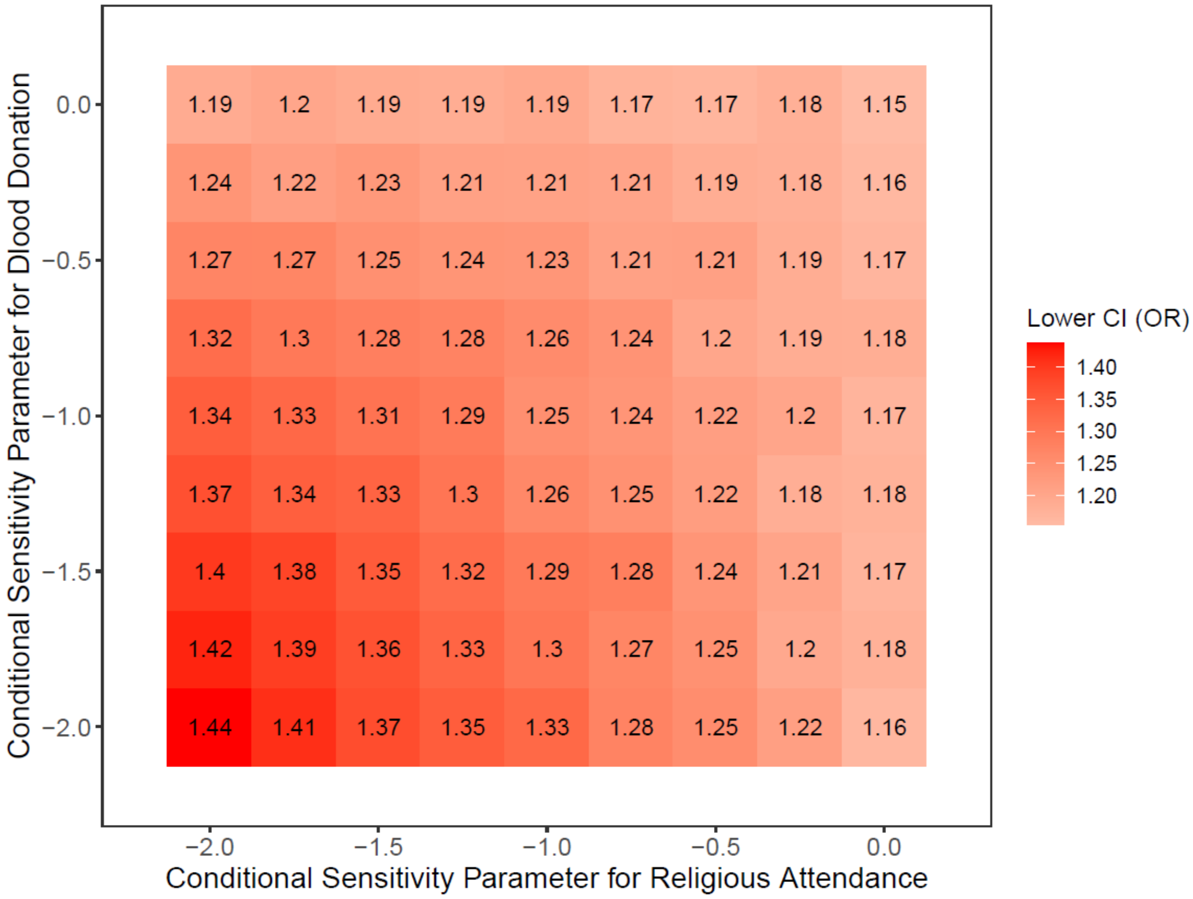

Figure 12c: Three plots with the blood donation conditional sensitivity parameter on the x-axis, with each plot a different conditional sensitivity parameter for religious attendance (at -2, -1 and 0, respectively). The odds ratio effect estimate of the exposure (regular religious attendance) on the outcome (blood donation) for these different conditional sensitivity parameters is given in blue, with lower and upper 95% confidence intervals in dashed green and red, respectively. The vertical red lines denote a conditional sensitivity parameter of 0 for the blood donation variable (i.e., data Missing-At-Random). The horizontal red lines denote a null association (odds ratio = 1).

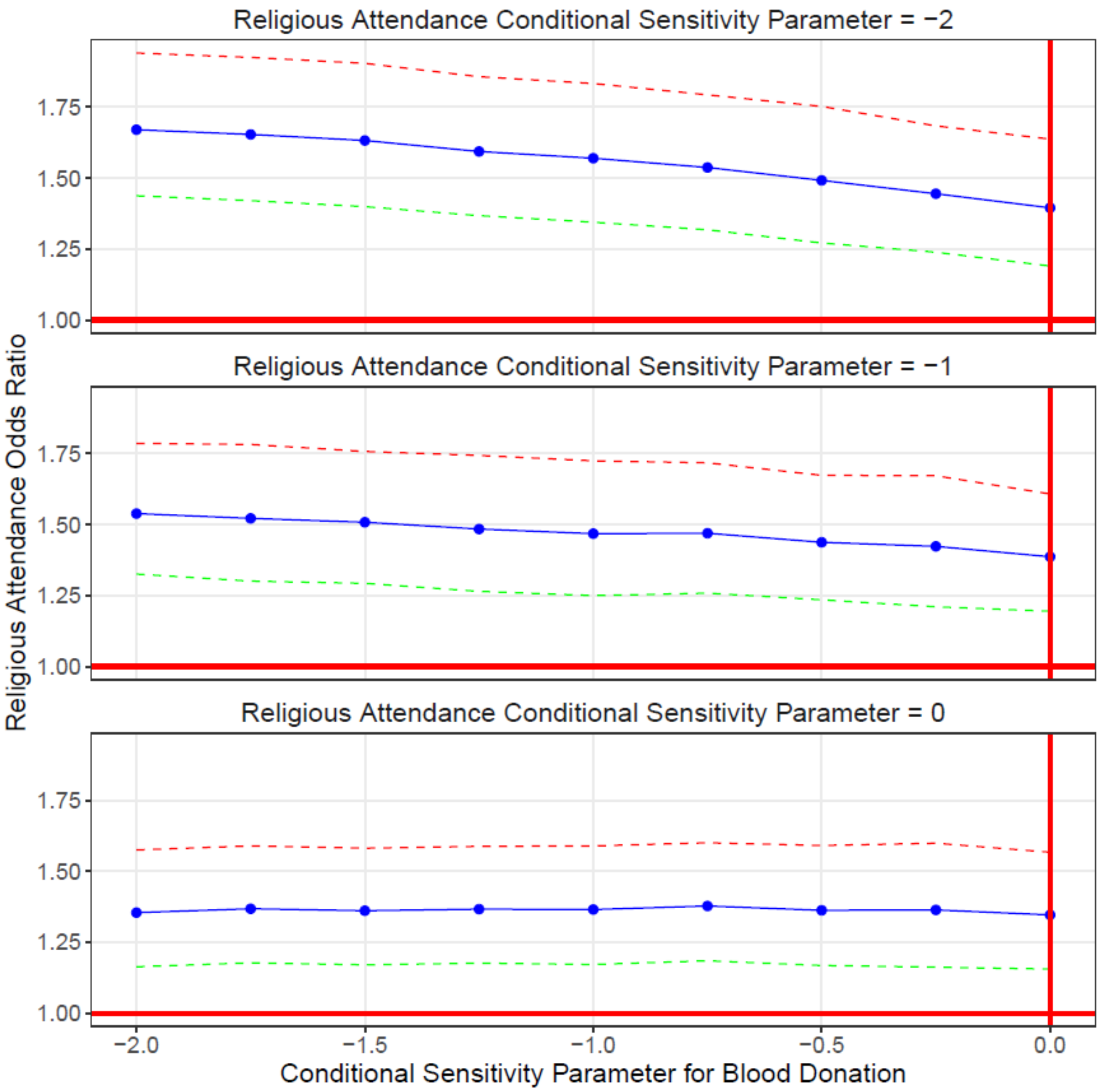

Supplement: Supplementary file 1 [file S2513843X23000178sup001.pdf]
